# De Novo Sequencing of Synthetic *Bis*-cysteine Peptide Macrocycles Enabled by “Chemical Linearization” of Compound Mixtures

**DOI:** 10.1021/acs.analchem.3c01742

**Published:** 2023-09-19

**Authors:** Zhi’ang Chen, Yi Wee Lim, Jin Yong Neo, Rachel Shu Ting Chan, Li Quan Koh, Tsz Ying Yuen, Yee Hwee Lim, Charles W. Johannes, Zachary P. Gates

**Affiliations:** † Institute of Molecular and Cell Biology (IMCB), Agency for Science, Technology and Research (A*STAR), 61 Biopolis Drive, Proteos, Singapore 138673, Republic of Singapore; ‡ Institute of Sustainability for Chemicals, Energy and Environment (ISCE^2^), Agency for Science, Technology and Research (A*STAR), 8 Biomedical Grove, #07-01 Neuros, Singapore 138665, Republic of Singapore

## Abstract

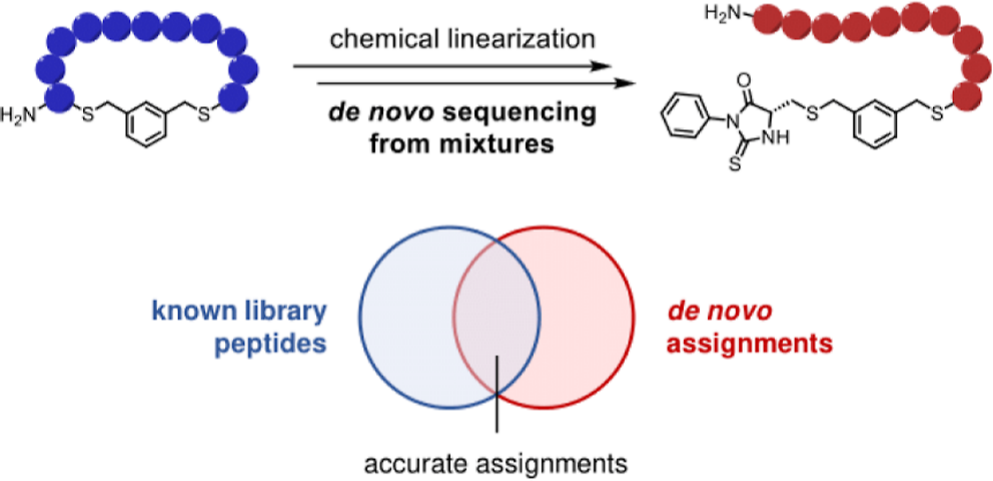

A “chemical linearization” approach was applied to synthetic peptide macrocycles to enable their de novo sequencing from mixtures using nanoliquid chromatography–tandem mass spectrometry (nLC–MS/MS). This approach—previously applied to individual macrocycles but not to mixtures—involves cleavage of the peptide backbone at a defined position to give a product capable of generating sequence-determining fragment ions. Here, we first established the compatibility of “chemical linearization” by Edman degradation with a prominent macrocycle scaffold based on *bis*-Cys peptides cross-linked with the *m*-xylene linker, which are of major significance in therapeutics discovery. Then, using macrocycle libraries of known sequence composition, the ability to recover accurate de novo assignments to linearized products was critically tested using performance metrics unique to mixtures. Significantly, we show that linearized macrocycles can be sequenced with lower recall compared to linear peptides but with similar accuracy, which establishes the potential of using “chemical linearization” with synthetic libraries and selection procedures that yield compound mixtures. Sodiated precursor ions were identified as a significant source of high-scoring but inaccurate assignments, with potential implications for improving automated de novo sequencing more generally.

## Introduction

Mass spectrometry (MS) coupled to liquid chromatography is ideally suited for the analysis of compound mixtures, with applications across scientific disciplines including combinatorial chemistry.^1^ Peptide macrocycles derived from combinatorial libraries are of major interest in therapeutics discovery,^2–4^ and the ability to sequence peptide macrocycles from mixtures would be enabling (for example, in ligand discovery by “affinity selection-mass spectrometry” (AS-MS)^5,6^). In particular, methods applicable to thioether-linked macrocycles comprising >10 amino acid residues—arguably the most significant family with respect to ligand discovery^4^—would be valuable. Synthetic libraries have proven valuable tools for benchmarking peptide identification by tandem MS in proteomics,^7–9^ and analogous tools are needed with respect to peptide macrocycles.

Sequencing macrocycles using MS is a long-standing challenge,^10^ and recent approaches based on both database searching^11^ and novel de novo algorithms^12,13^ applicable to cyclic and branch-cyclic peptides have been described. An alternative strategy involves chemical cleavage of the peptide backbone at a defined position,^14–25^ to generate linear peptides with fragmentation behaviors that are more predictable and less complex. This “chemical linearization” approach is attractive because it is, in principle, compatible with existing de novo sequencing algorithms. However, prior work has focused on individual substrates only, and based on these precedents, it is not obvious whether an extension to complex mixtures would be feasible. Additionally, prior work has focused largely on “head-to-tail” macrocycles of small ring size (<8 amino acids),^14–25^ and there remains a significant gap with respect to thioether-linked macrocycles of relevance to ligand discovery.^2–4^

**Figure 1:**
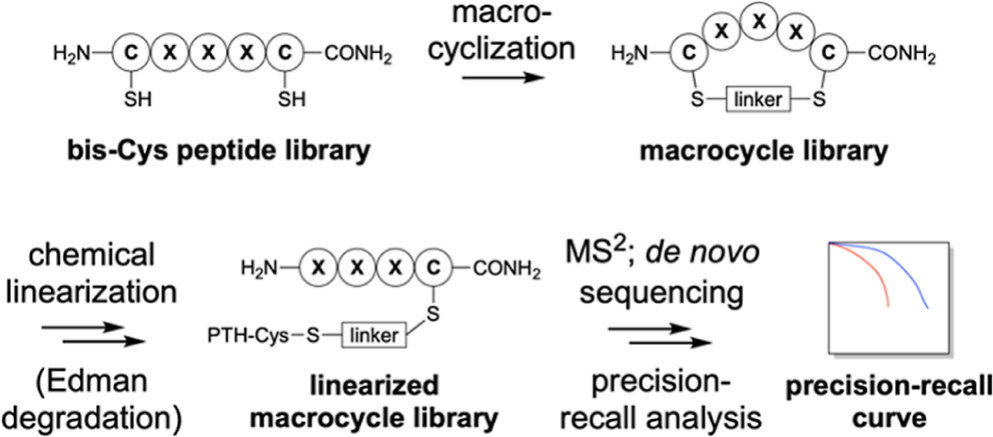
Experimental workflow established here to evaluate “chemical linearization” as an approach to de novo MS/MS sequencing of synthetic macrocycle mixtures. Chemical structure of “Cys-PTH” is shown in Figures 2 and S6A.

Here, we describe a framework for benchmarking chemical linearization as an approach to MS-based sequencing of peptide macrocycles using synthetic mixtures comprising hundreds of unique compounds. The framework involves first establishing the efficiency of the relevant chemical transformations using individual model compounds. Subsequently, with confidence in the underlying chemical steps, the same conditions are applied to chemical libraries of known sequences based on the model peptides. By constraining these libraries to compound numbers compatible with nLC–MS/MS analysis, an entire library can then be sequenced, and candidate de novo assignments can be evaluated by comparison to the known library peptides (Figure 1). Evaluating chemical linearization on compound mixtures enables the use of performance metrics that are not applicable to individual compounds, thus allowing comparison with de novo sequencing of linear peptides in a fashion not otherwise possible.

We applied this framework to sequence mixtures of thioether-linked macrocycles derived from *bis*-Cys peptides using chemical linearization by Edman degradation.^22,26^ The results across three libraries vary by context but in general suggest that linearized macrocycles can be sequenced with similar accuracy to linear peptides, albeit with lower recall (the fraction of total peptides in a mixture that are accurately sequenced). Recall is shown to vary with peptide ion abundances, which suggests a plausible link between peptide recall and the chemical linearization yield. Finally, some common errors in de novo sequencing by the commercial software PEAKS are discussed. Erroneous assignments made to sodiated precursor ions were identified as a significant category missed by typical benchmarking and which may be of broader relevance outside the context of macrocycles.

## Experimental Section

### Macrocyclization of *Bis*-Cys Peptides

Lyophilized *bis*-Cys peptide (e.g., Zpep(“SH”), 5 mg, 4.0 μmol) was dissolved in aqueous ammonium bicarbonate buffer (6.85 mL; 20 mM ammonium bicarbonate, pH 7.8; degassed by sparging with argon), and dithiothreitol was added (160 μL of a 100 mM solution in ammonium bicarbonate buffer). After 5 min, α,α′-dibromo-*m*-xylene was added (1 mL of a 21 mM solution in degassed acetonitrile), and the resulting solution was kept for 30 min before washing with cold diethyl ether (3 × 8 mL; from the Pure Process Technology solvent purification system). The aqueous phase was diluted 4-fold into 6 M guanidinium chloride, 100 mM phosphate, pH 6.9 buffer, and the product was isolated by solid-phase extraction using a Maxi-Clean 300 mg C_18_ cartridge (S*Pure, Singapore). Lyophilization afforded the macrocycle as a white solid (e.g., Zpep(“S-**mxy**-S”), 5.60 mg, 4.15 μmol, 104% yield). Peptide libraries were handled analogously for this and subsequent procedures, using the average molecular weight of each library to calculate the molar quantities of the total peptide.

### Phenyl Isothiocyanate Conjugation of *Bis*-Cys Macrocycles

Lyophilized peptide macrocycle (e.g., Zpep(“S-**mxy**-S”), 1.44 mg, 1.07 μmol) was dissolved in a medium comprising 3:2 pyridine/aqueous *N*-methylmorpholine buffer (0.75 mL total; the aqueous component was 1.3 M *N*-methylmorpholine, 250 mM trifluoroacetate, pH 8.1). The resulting solution was treated with 3.33% (v/v) phenyl isothiocyanate (PITC) in acetonitrile (0.32 mL) for 10 min, then diluted with 0.1% aqueous trifluoroacetic acid (9.6 mL) and centrifuged (4000 rpm, 15 min). The supernatant was washed with diethyl ether (3 × 10.7 mL), and the product was isolated by solid-phase extraction (as above). Lyophilization afforded the PITC conjugate as a white solid (e.g., PITC-Zpep(“S-**mxy**-S”), 0.75 mg, 0.50 μmol, 47% yield).

### Edman Linearization of PITC-Conjugated Macrocycles

Lyophilized PITC conjugate (e.g., PITC-Zpep(“S-**mxy**-S”), 0.75 mg, 0.50 μmol) was dissolved in trifluoroacetic acid (75 μL) and kept for 10 min. Deionized water was added (0.3 mL), and the resulting solution was heated to 70 °C for 10 min in a temperature-controlled water bath. Aqueous 6 M guanidinium chloride, 100 mM phosphate, pH 7 buffer was then added (1.13 mL), and the product was isolated by solid-phase extraction using a Mobicol “F” column (MoBiTec, Gottingen, Germany) packed with Oasis HLB resin (∼30 mg, 30 μm; Waters, Milford, MA, USA). Lyophilization afforded the linearized macrocycle as a white solid (e.g., linearized Zpep(“S-**mxy**-S”), 0.66 mg, 0.44 μmol, 88% yield).

### nLC–MS/MS Analysis of Peptide Libraries

Lyophilized library (e.g., linearized Zpep(“S-**mxy**-S”), 0.14 mg, 0.11 μmol total, and 0.21 nmol/peptide) was dissolved in 50/50 acetonitrile/water (21.7 μL; MS-grade solvents containing 0.1% trifluoroacetic acid). The resulting solution (5.0 mM total library; 9.8 μM/peptide) was diluted serially into the mobile phase (2.5% MS-grade acetonitrile, 97.5% MS-grade water, and 0.05% acetic acid) containing Pierce Peptide Retention Time Calibration Mixture (2 fmol/peptide/μL) to final concentrations of 10, 5.1, or 1.0 μM total library (20, 10, or 2 nM/peptide, respectively). 5 μL portions of these solutions (containing 100, 50, or 10 fmol/peptide) were analyzed using an Easy-nLC 1200 nanoliquid chromatography system equipped with a Pepmap RSLC C18 analytical column (2 μm, 100 Å, 75 μm × 50 cm) and an Acclaim PepMap 100 trap column (3 μm, 100 Å, 75 μm × 2 cm) connected to an Orbitrap Fusion Lumos mass spectrometer (Thermo Fisher Scientific, Waltham, MA, USA). Full details of the instrumental methods are provided in the Supporting Information.

## Results and Discussion

### Preparation of Model Macrocycles for Use as “Chemical Linearization” Substrates

Thioether-forming chemistries exhibit broad substrate scope and high selectivity,^27,28^ and are widely used for the preparation of macrocycle libraries in mRNA^29,30^ and phage display.^31^ Therefore, because of their relevance to ligand discovery and to ensure that the peptide libraries were productively cyclized, we decided to focus on thioether macrocycles accessed from *bis*-Cys precursors. To this end, three specific linkers were considered for the preparation of model substrate macrocycles: α,α′-dibromo-*m*-xylene (**mxy**),^32^ pentafluorophenyl sulfide (**pps**),^33^ and diiodomethane.^34^

**Figure 2:**
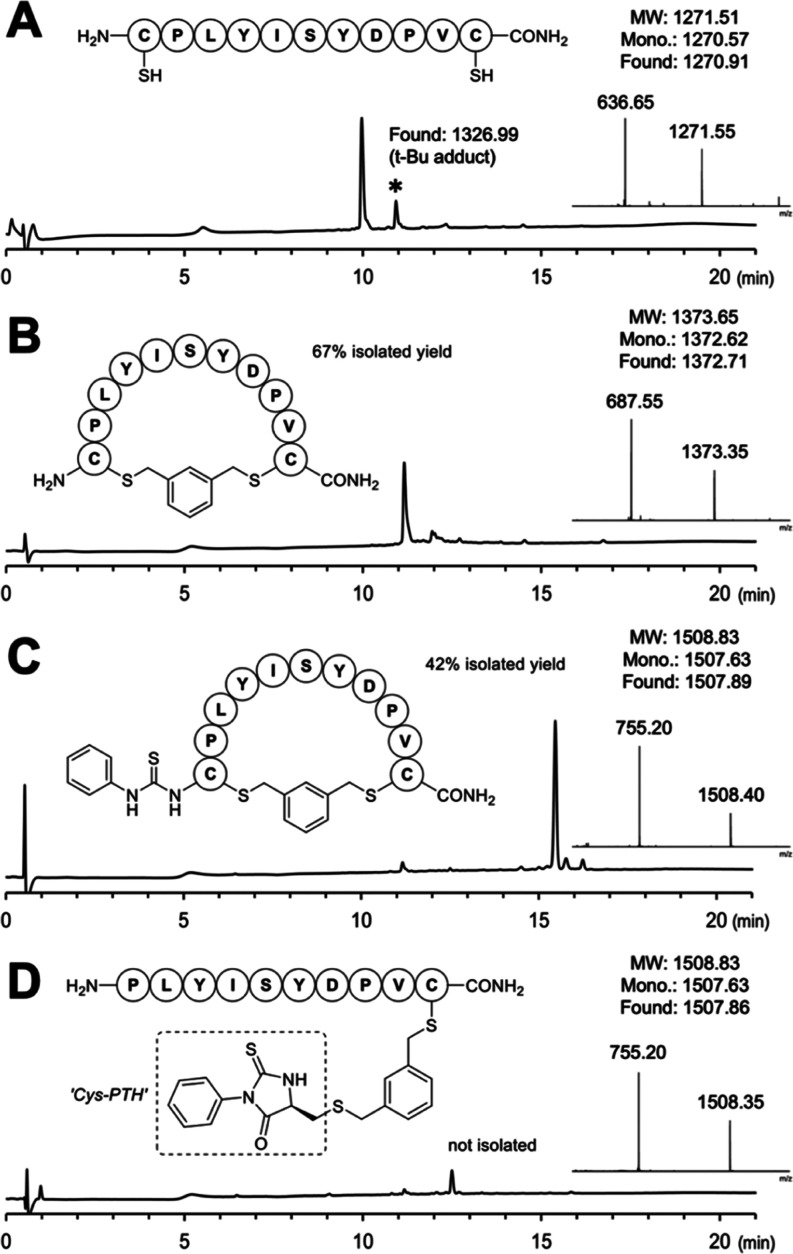
The model peptide “KRpep” undergoes clean conversion from *bis*-Cys peptide to linearized macrocycle product over a three-step macrocyclization/chemical linearization sequence. (A–D) LC–MS analysis of the *bis*-Cys peptide starting material and each of the three reaction products. UV chromatograms are shown (equivalent *y*-axis range throughout; 5 μg nominal sample loadings), with inlaid mass spectra integrated across the principal UV component. Crude products were isolated by solid-phase extraction after each step and used without further purification. Analogous data for **mxy** macrocycles derived from “Zpep” and “PMI” are shown in Figures S3 and S5.

The efficiency of macrocyclization by these reagents was first studied using two model peptides: “KRpep” (sequence: CPLYISYDPVC), a truncated form of the K-Ras inhibitory peptide KRpep-2d,^35^ which was previously used for the evaluation of de novo peptide sequencing,^36^ and “Zpep” (sequence: CFTNGLLYESC), adapted from a peptide used to establish the substrate scope of perfluorarylation by reagents including **pps**.^33^ These experiments revealed that diiodomethane macrocyclization was not robust under a variety of conditions, with negligible quantities of product formed even after 18 h (however, the desired macrocycle was obtained as major product when the N-terminus was protected; see Figure S1). Conversely, **mxy** and **pps** macrocyclization proceeded cleanly and rapidly with nearly quantitative conversion (Figure 2; Figures S2–S4). As such, only **mxy** and **pps** macrocycles were carried forward.

### Linearization of Model Substrates Using Edman Degradation

Edman degradation is a well-established method to cleave the peptide bond adjacent to an alpha-amino acid with a free N-terminus,^37^ and was recently used to enable sequencing of individual macrocycles following one-bead one-compound library screening.^22,26^ Therefore, we selected Edman degradation as a linearization method that could be applied with confidence to libraries and could hence act as a starting point for extending chemical linearization to compound mixtures. Subjecting *bis*-Cys macrocycles to Edman degradation should yield linear products, provided one of the two Cys residues is N-terminal (Figures 1 and 2).

The Edman degradation reaction sequence here involved two steps, with isolation by solid-phase extraction after each step. First, the model macrocycles were treated with PITC in a reaction medium consisting of pyridine, acetonitrile, and a pH 8.1 aqueous buffer comprising trifluoroacetic acid and *N*-methylmorpholine.^38^ These conditions gave quantitative conjugation of PITC to the peptide N-terminus for each of the four model peptides, as reflected by LC–MS reaction monitoring (Figure 2; Figures S2–S4). For Zpep(“S-**pps**-S”), a low isolated yield was obtained (Figure S2), and therefore only three of four PITC macrocycles were carried forward.

In the second step, the PITC macrocycles were treated with neat trifluoroacetic acid, followed by dilution with water to 20% trifluoroacetic acid and heating (70 °C, 10 min).^39^ For each of the two PITC-**mxy** macrocycles, LC–MS analysis reflected quantitative conversion to a reaction product of distinct retention time but identical mass (within experimental uncertainty), consistent with the expected ring-opening of the macrocycle (Figure 2; Figure S3). In contrast, the **pps** macrocycle PITC-KRpep(“S-**pps**-S”) gave a mixture of products for which the desired product was a minor component and present for only one of two experimental replicates (Figure S4). Based on these results, we concluded that of the macrocycles tested, only those based on the **mxy** linker were compatible with Edman degradation, and we focused on these macrocycles moving forward. To extend these conclusions, the compatibility of **mxy** macrocycles with Edman degradation was verified in a third system of larger ring size (“PMI”; sequence: CTSFAEYWNLLSPC), based on an MDM2-binding peptide from phage display^40^ (Figure S5).

### Preparation, Linearization, and nLC–MS/MS Analysis of Macrocycle Libraries

Having established chemical linearization for model **mxy** macrocycles, the reaction sequence was applied to macrocycle libraries of known composition to determine whether de novo sequencing of the resulting product mixtures returned the known library peptides. For this purpose, libraries of “KRpep” and “Zpep” were prepared, comprising all combinations of Ala substitutions at the non-Cys positions. A third library was prepared based on “PMI”, where three non-Cys residues were fixed such that each library comprised the same number of peptides (**C**TS**F**AEY**W**NL**L**SP**C**; fixed residues in bold; the Ala residue was varied as either Ala or Gly). These libraries were accessed conveniently by “split-mix” synthesis and comprised 512 unique peptides each in total (9 variable positions; wild type or Ala/Gly at each variable position; 2^9^ combinations)—a number sufficiently low such that the entire library could be analyzed by nLC–MS/MS without any anticipated loss in coverage.

After solid-phase synthesis, the libraries were simultaneously deprotected and cleaved from the resin, then carried through a 3-step reaction sequence consisting of macrocyclization (1 step), followed by chemical linearization (2 sequential steps). In parallel, controls were prepared by treating the *bis*-Cys libraries with 2-bromoacetamide to give linear acetamide-capped libraries that would report on their amenability to de novo sequencing, independent of chemical linearization. All reactions were conducted on a similar scale to the model peptides with respect to total peptide content (e.g., ∼0.5 μmol total; 1 nmol/peptide; see the Supporting Information) so that they could be handled analogously. After isolation of the final reaction products, stock solutions were made and diluted for Orbitrap-MS analysis at nominal sample loadings of 10, 50, and 100 fmol/peptide, as well as 200 fmol/peptide for the linearized “PMI” macrocycles (see the Experimental Section).

### De Novo Sequencing and Data Analysis

Orbitrap-MS data was processed via PEAKS Studio for de novo sequencing,^41^ incorporating the “S-**mxy**-S-Cys-PTH” moiety as a fixed post-translational modification of the C-term Cys residue. The diagnostic fragment ion corresponding to this modified Cys was present in many of the raw MS^2^ spectra from the linearized libraries, consistent with the covalent structure assigned to the linearized macrocycles (Figure S6). Likewise, the analogous *y*_1_ fragment corresponding to acetamide-modified Cys was present in the spectra from the linear acetamide libraries.

**Figure 3:**
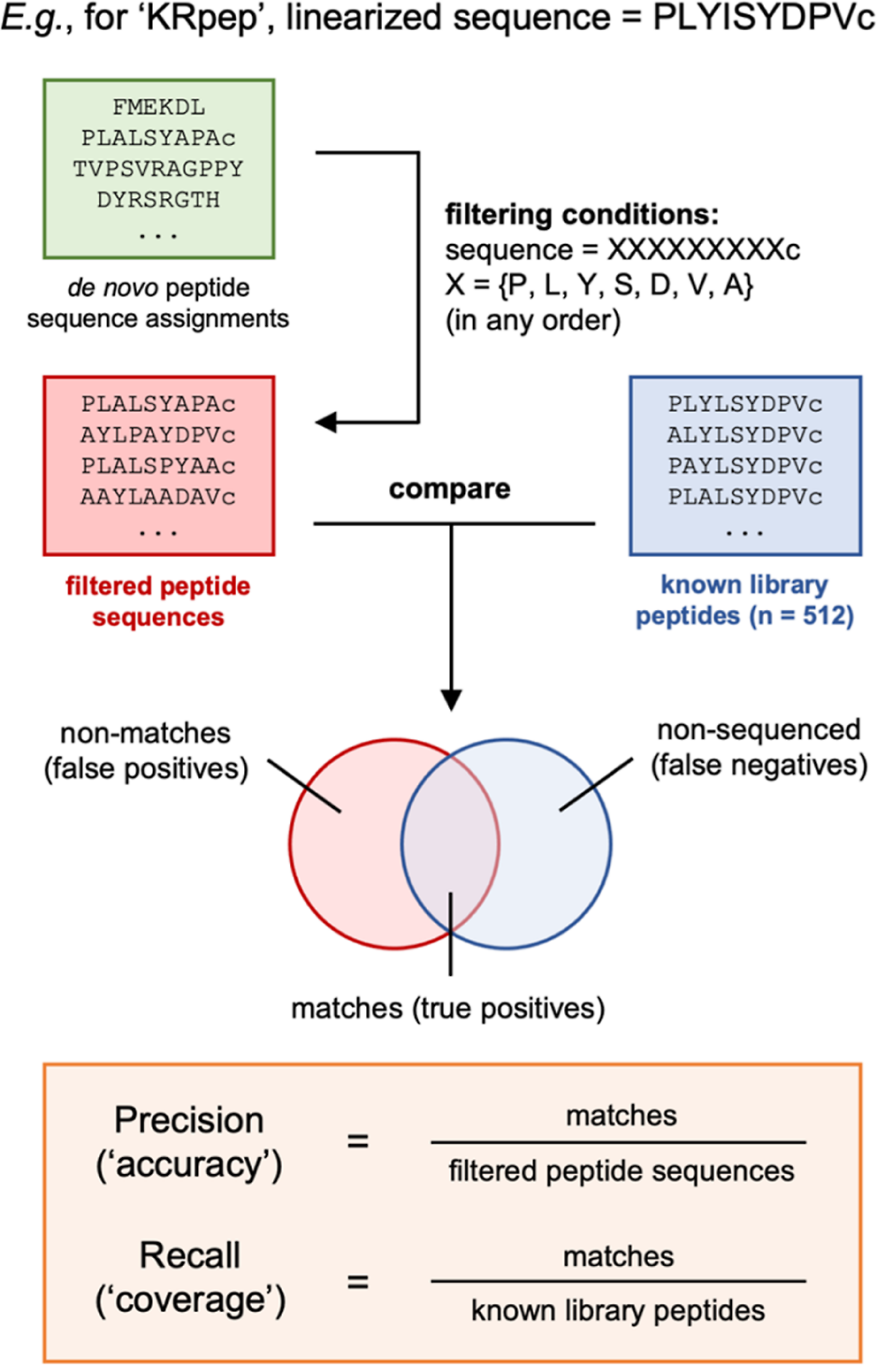
Schematic illustration of the data analysis workflow used to evaluate de novo sequence assignments by comparison with the known library peptides. As above, “c” corresponds to the modified Cys structure shown in Figures 2 and S6A.

To evaluate both the accuracy of candidate de novo assignments and the fraction of the library that was sequenced, the raw PEAKS output was compared to the known library peptides as described^36^ (Figure 3). First, the raw de novo output was filtered to remove assignments of incorrect length, that contained amino acids not part of the library design, or that lacked the correct terminal residues (N- and C-terminal Cys(“S-acetamide”) for the control libraries and C-terminal Cys(“S-**mxy**-S-Cys-PTH”) for the linearized macrocycles). The filtered peptide sequences were then compared against known library peptides to identify matches (true positives), nonmatches (sequence candidates not part of the known library peptides, false positives), and nonsequenced peptides (known library peptides not part of sequence candidates, false negatives). These comparisons were quantified in precision–recall curves, where precision and recall are plotted after the removal of de novo assignments below a varied PEAKS score threshold.^42,43^ By comparing precision–recall curves for the linearized **mxy** macrocycles and linear acetamide libraries, the relative ability to sequence linearized macrocycles could be assessed.

**Figure 4:**
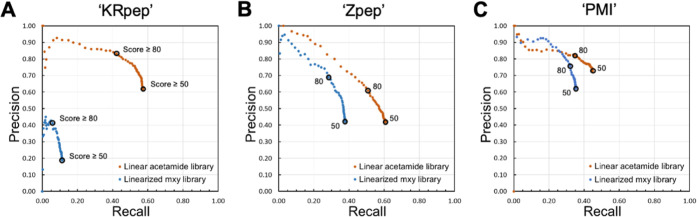
Libraries of linearized macrocycles can be sequenced with similar accuracy to linear peptides. Precision–recall curves for pairs of the linearized macrocycle (“S-**mxy**-S”; blue) and linear acetamide (“S-acetamide”; orange) libraries based on (A) “KRpep”; (B) “Zpep”; and (C) “PMI”. For each sample, the precision and recall values at PEAKS score thresholds of ≥50 and ≥80 are indicated. The nLC–MS/MS data were acquired at sample loadings of 100 fmol/peptide, in triplicate with the exception of the linearized PMI macrocycles, acquired at 200 fmol/peptide to enable comparison at the most comparable ion abundances (see below); precision and recall values were calculated as the average over three technical replicates.

Overall, the three linear acetamide libraries yielded broadly similar precision–recall curves, with “KRpep” reproducing a published result^36^ and both “Zpep” and “PMI” extending these results to new systems (Figure 4; orange traces). In contrast, outcomes for linearized macrocycles varied dramatically between libraries. For “Zpep” and “PMI”, linearized **mxy** libraries were generally sequenced with comparable precision to linear acetamide libraries across a range of score thresholds, with some loss in recall (∼40% and ∼35% for linearized macrocycles vs ∼60 and ∼45% for linear libraries based on “Zpep” and “PMI”, respectively, at PEAKS score ≥50; Figure 4B,C). For example, at a score threshold = 80 (a recommended starting point for analyzing unknown mixtures),^36^ the precision achieved for linearized macrocycles exceeded that of linear acetamide peptides in the “Zpep” system and was within 10% for “PMI”. However, for “KRpep”, major losses in both precision and recall were apparent (Figure 4A).

We endeavored to understand at the MS^2^ level why many “KRpep” linearized macrocycle assignments were erroneous. Inspection of individual spectra revealed that in many cases, the low *b*-ions/high *y*-ions necessary to define the correct N-terminal region were absent or of low intensity (particularly *b*_1_ and *y*_9_). As a result, many assignments contained errors at the peptide N-terminus, as evident from a WebLogo plot of amino acid positional frequencies (Figure S7). To account for this effect, we calculated precision–recall curves using datasets comprising the 8 C-terminal residues only (Figure S8). For these subsequences (128 total), precision improved to the level of linear controls—analogous to the “Zpep” and “PMI” libraries—with lower recall across the range of score thresholds. Thus, when this fragmentation issue observed for “KRpep” was accounted for, results similar to “PMI” and “Zpep” were achieved.

Our conclusions were confirmed using a complementary approach to evaluating de novo assignment accuracy. In this approach (Figure S9, “Method 2”), we took advantage of the fact that for each library, some proportion of peptides have a unique mass, distinct from all others in the library (64 of 512 for “KRpep” and 144 of 512 for “Zpep” and “PMI”). De novo assignments to these “unique mass peptides” were evaluated based on whether they matched the unique sequence encoded by their precursor ion mass. Results obtained by this approach were in broad agreement with those above (Figure S10), thus confirming that our original method did not overestimate either precision or recall (e.g., if erroneous de novo assignments incidentally matched library peptides).

Together, the results across three systems demonstrate that with respect to accuracy (here, “precision”), “chemical linearization” can yield de novo assignments of similar quality to those obtained for linear peptides. However, with respect to coverage (the fraction of total peptides in the mixture that are sequenced; “recall”), there is a gap—with linearized macrocycles returning ∼65% of the recall achieved for linear peptides (∼60% for “Zpep”, ∼80% for “PMI”, and ∼50% for “KRpep” subsequences). As discussed below, peptide recall correlated with both nLC–MS/MS sample loading and precursor ion abundances, which suggests that the lower recall of linearized macrocycles may be due to reaction yields inherent to the combined macrocyclization/linearization sequence.

### Effect of Sample Loading

Across all libraries, higher sample loadings correlated with improved recall with little effect on precision (Figure S11). For the linear acetamide libraries, matches were obtained at all loadings examined (10, 50, and 100 fmol/peptide), with a major gain in recall between 10 and 50 fmol/peptide. In contrast, for the linearized macrocycles, matches were obtained at higher loadings only (50 and 100 fmol/peptide for “KRpep” and “Zpep” and 100 and 200 fmol/peptide for “PMI”).

Since sample loading was a significant determinant of recall within individual samples and because recall of acetamide peptides and linearized macrocycles differed for equivalent nominal loadings, the results suggest that linearized macrocycles are present in lower abundance at a given nominal loading compared to acetamide peptides. This interpretation is supported by the magnitude of peptide ion abundances, which were consistently lower for linearized macrocycles at equivalent nominal loadings (Figure S12). As discussed above, abundance could be affected by either recovery losses over multiple handling steps or nonquantitative yields for any of the chemical transformations and may vary between individual peptides.

### Common Errors in De Novo Assignments and the Role of Sodium (Na) Adducts

Typically, de novo sequencing is evaluated using only those MS^2^ spectra with high-confidence “peptide spectrum matches” (PSMs) from database searching. By using this approach, it is straightforward to detect and classify errors by comparing candidate de novo assignments with the associated PSM.^44–46^ In contrast, the main approach used here (Figure 3) and described elsewhere^36,43^ estimates accuracy at the level of sequenced peptides. By this “PSM-agnostic” approach, individual spectra are not linked one-to-one with “ground truth” peptides, but spectra beyond those that would receive PSMs are potentially considered. As a result, the approach may sample de novo errors that are relevant to actual use cases (i.e., that are present in the de novo output obtained from raw MS datasets) but would be missed by typical benchmarking.

To gain insight into errors common among high-scoring de novo assignments for the “KRpep” and “Zpep” libraries, we examined the 10 top-scoring assignments that were of correct length but did not match a library peptide (all PEAKS scores >90). A significant fraction of these misassignments resulted from precursor ions other than protonated library peptides. For example, a large number were likely sodium (Na) adduct precursors based on their coelution with precursors of the expected *m*/*z* difference (10.991 Da/e, for doubly charged precursors; 16 of 40 misassignments; 40% of the total; Figure S13A). Of the remaining misassignments (24 of 40), a significant number were made to precursors that did not correspond to expected peptide masses (8 of 40; 20% of the total). Misassignments classified elsewhere^44,45^ as either “inversion” (misordering of otherwise correct residues; 14 of 40; 35%), “substitution” (inclusion of incorrect amino acid residues which are isobaric with the true sequence, e.g., “Ala + Tyr” vs “Ser + Phe”; 1 of 40; 2.5%), or a combination of “inversions” and “substitutions” (1 of 40; 2.5%) were also present (Figure S14).

**Figure 5:**
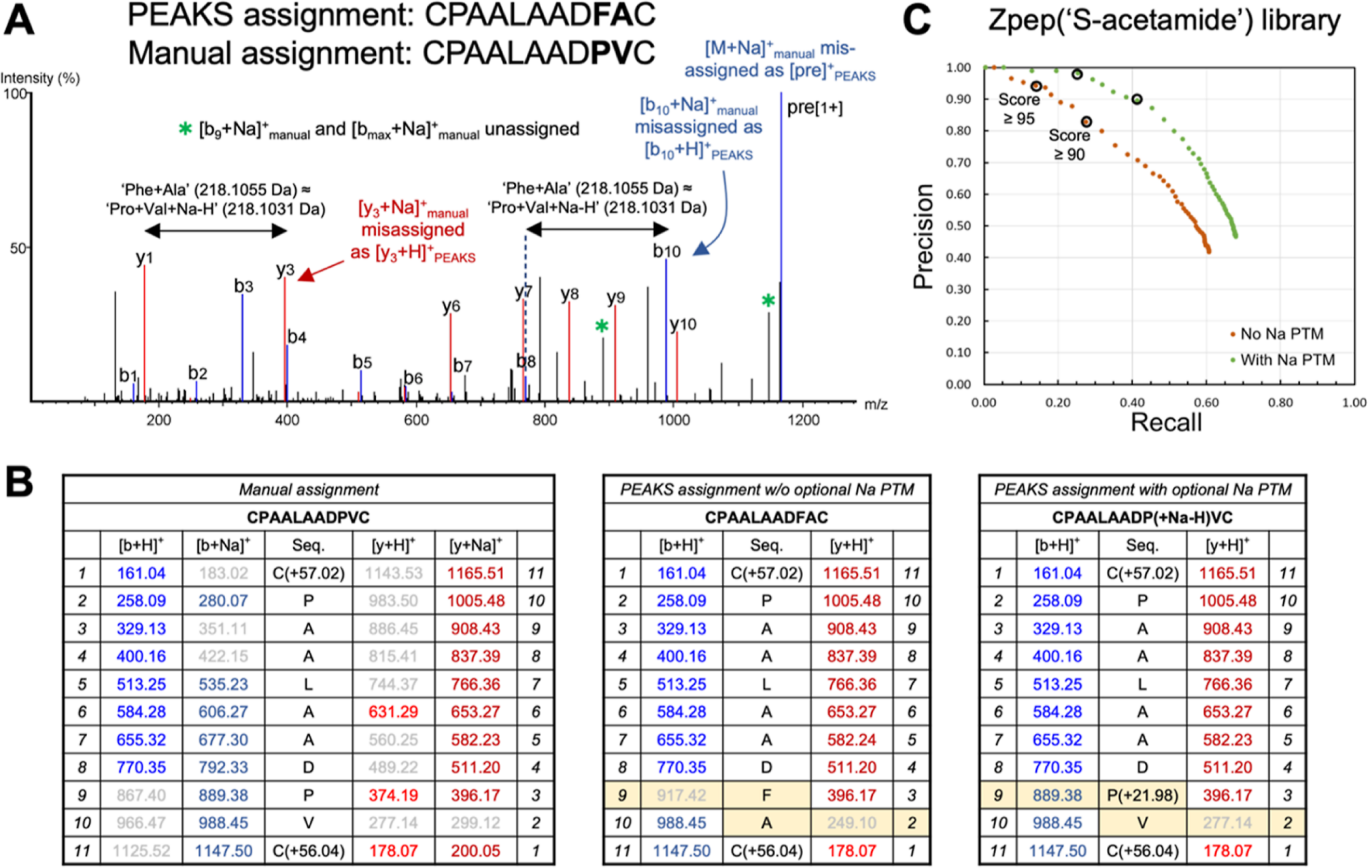
MS^2^spectra of sodiated peptides can be misinterpreted due to near-isobaric ambiguities involving Na and their misassignments corrected by formal post-translational modification. (A) An MS^2^ spectrum collected on the Na adduct of a KRpep(“S-acetamide”) peptide, indicating a “[*y*_3_ + Na]_manual_^+^” fragment that was misassigned as “[*y*_3_ + H]_PEAKS_^+^”; the difference “[*y*_3_ + Na]_manual_^+^ – [*y*_1_ + H]_manual_^+^” corresponds to the erroneous dipeptide “Phe–Ala”. Because *y*_3_ fragments and above were all present as “[*y*_*n*_ + Na]_manual_^+^”, their mass differences correspond to the correct amino acid sequence, even though each was misassigned as “[*y*_*n*_ + H]_PEAKS_^+^”. Likewise, in the *b* ion series, the “Phe–Ala” dipeptide was accommodated by misassignment of “[*b*_10_ + Na]_manual_^+^” as “[*b*_10_ + H]_PEAKS_^+^”; *b*_8_ fragments and below were all present as [*b*_*n*_ + H]^+^. (B) Fragment ion tables, illustrating how an optional “Na PTM” (see main text) leads to the correct assignment of the *b*_9_ fragment ion (889.38; right-most table) and inference of the correct sequence. Fragment ions shown in gray were absent, and Na adducts are in darker shading. (C) Precision–recall curves for the Zpep(“S-acetamide”) library, illustrating the effect of allowing an optional “Na PTM” during de novo sequencing (+21.9819 Da, corresponding to “Na minus proton”).

Because Na adducts contributed a significant number of misassignments, we examined the nature of their spectra and how they were interpreted incorrectly despite high confidence scores (Figure 5). These misassignments result from the combination of two factors: first, the existence of near-isobaric ambiguities involving Na (e.g., “Phe + Ala” ≈ “Pro + Val + Na – H”; “Tyr + Ala + Ala” ≈ “Pro + Ser + Val + Na – H”; “Tyr + Ala + Ser” ≈ “Gly + Leu + Glu + Na – H”; and “Thr + Tyr” ≈ “Leu + Glu + Na – H”; all have mass difference = 0.0024 Da) and second, the tendency of individual fragment ions derived from Na adduct precursors to occur largely as a single adduct form, with proton adducts predominant for lighter fragments and Na adducts for heavier fragments (Figure S13C), echoing previous findings on lithiated precursors.^47^ Where the predominant adduct form changes, a mass difference of “+Na – H” results that is interpreted as an isobaric substitution (Figure 5A). Prior work on de novo sequencing applied to synthetic libraries noted the occurrence of Na adducts in filtered datasets but did not delineate their origin at the MS^2^ spectrum level.^43^

In some cases, misassignments to sodiated precursors were corrected using an optional post-translational modification, which accounts for the mass difference between Na and proton adducts (+21.98; “Na PTM”). By incorporating this mass difference in the region where a fragment ladder changes adduct forms (e.g., at Pro9, such that fragment *b*_9_ incorporates the mass difference; Figures 5B and S13B,C), isobaric substitutions could be avoided and the correct sequences inferred.

To study the potential of this tactic on a larger scale, we performed de novo sequencing of each library, either with or without the use of an optional “Na PTM” (allowed at any residue type, “variable @[X]”). Precision–recall curves for the resulting filtered peptides showed general improvements in both precision and recall, with the clearest effect for the Zpep(“S-acetamide”) library, where recall improved by ∼50% at higher score thresholds (Figure 5C and Figure S15). A clear effect was also seen for KRpep(“S-acetamide”)—where precision improved by ∼20% across a range of score thresholds—after relaxing filtration of the raw de novo output (Figure S16). Evidently, in this case, misassignments to Na adducts contained residues absent from the library (e.g., Phe, Figure 5a), which were removed by filtration prior to precision–recall analysis in the standard workflow. Further studies are needed to generalize these findings for a wider range of sample types. Importantly, the main conclusions regarding chemical linearization were unaffected since similar precision was achieved for linear acetamide and linearized macrocycle libraries regardless of “Na PTM” usage (Figures S15 and S16; for “PMI” and “Zpep”, comparing precisions at score threshold = 80, as above).

## Conclusions

The results presented here establish that chemical linearization of synthetic macrocyclic peptides can be applied to compound mixtures, which enables their de novo sequencing with fidelity comparable to that of linear peptides using established software. This is a significant advance and suggests that a variety of other chemical linearization methods used with individual macrocycles may be similarly effective for this purpose.^14–25^ In addition, the work extends the chemical linearization approach to Cys-based, thioether-linked macrocycles of the type used in phage and mRNA display for novel ligand discovery, which are of emerging significance as therapeutics^48^ and comprise a larger number of varied positions than typical synthetic libraries where chemical linearization has been employed.^14–25^

Edman degradation as a chemical linearization method is attractive in that it is, in principle, compatible with any “side chain-side chain” macrocycle with a free N-terminus and where one of two cross-linked residues is N-terminal. Edman degradation has already been used for chemical linearization of triazole-cross-linked macrocycles in the context of “one-bead one-compound” libraries,^22,26^ and should be compatible with a variety of other synthetic macrocycles, including hydrocarbon-stapled peptides, which are also of therapeutic interest. However, as a method applied to compound mixtures in solution, there are important limitations. For example, Edman degradation involves multiple chemical steps with different solvent requirements, which are not easily miniaturized in solution. In addition, nucleophilic side chains are modified by the Edman reagent, and although prior work has established these as compatible with MS/MS sequencing,^49^ we chose to exclude nucleophilic side chains from our libraries initially. An ideal method would involve a single step without intermediate product isolation, be fully compatible with unprotected peptides, and operate in aqueous buffers at high dilution.

Our specific interest is in methods that could be applied to synthetic libraries following AS-MS,^5,6^ to enable its use with peptide macrocycles. The reaction scale used here (100 pmol–1 nmol/peptide) is relevant to AS-MS applied to “focused” libraries of several thousand compounds (∼5 nmol/peptide),^5^ but would require further reduction for use with higher-diversity libraries (10 fmol/peptide).^6^ Even focused libraries offer considerable opportunity to accelerate the use of nonproteogenic amino acids in peptide engineering,^50^ and our work establishes the potential of chemical linearization for enabling the use of macrocycle libraries with AS-MS toward this goal.

Beyond this specific application, the framework employed here—based on critically testing a sequencing methodology using synthetic libraries of known composition—should prove valuable for benchmarking approaches to macrocycle sequencing more generally. Empirical benchmarking provides the confidence needed to analyze mixtures of unknown composition, and synthetic libraries combined with precision–recall analysis at the level of sequenced peptides^36,43^ are a valuable tool in this regard. A wide variety of macrocycles are accessible synthetically,^51^ and could prove useful for benchmarking sequencing approaches beyond chemical linearization (for example, novel de novo algorithms applicable to cyclic peptides^13^). Compared to standard benchmarking approaches for de novo sequencing, which evaluate assignments to a subset of experimental spectra with peptide-spectrum matches from database searching,^44–46^ the approach here provides a more realistic assessment of the performance expected in practice by considering assignments to a wider variety of spectra. As illustrated for the case of Na adducts, these spectra can give rise to significant categories of misassignments and provide opportunities for improving de novo sequencing for a variety of applications moving forward.

## Supporting Information Available

Experimental procedures; LC–MS characterization of model compounds; and supporting figures illustrating: preparation of model macrocycles and their chemical linearization; structures of diagnostic fragment ions derived from linearized macrocycles; WebLogo sequence plots for all libraries; analysis of KRpep subsequences; complementary approach to evaluating de novo assignment accuracy; effect of sample loading on sequencing outcomes; variation of peptide ion abundance with sample loading; use of “post-translational modification” to correct misassignment of a Na adduct spectrum; high-scoring misassignments from four libraries; and effect of an optional “post-translational modification” on sequencing outcomes for all libraries (PDF)
