# Supplementary material for: De Novo Sequencing of Synthetic Bis-cysteine Peptide Macrocycles Enabled by “Chemical Linearization” of Compound Mixtures
Source: Anal Chem. 2023 Sep 19;95(40):14870–8. doi: 10.1021/acs.analchem.3c01742 (PMC10569172; doi:10.1021/acs.analchem.3c01742)
Supplement: Supplementary file 1 — ac3c01742_si_001.pdf [file ac3c01742_si_001.pdf]

Supporting Information

De novo sequencing of synthetic *bis*-cysteine peptide macrocycles enabled by 'chemical linearization' of compound mixtures

Zhi'ang Chen[1,2,3], Yi Wee Lim[2], Jin Yong Neo[2,4], Rachel Shu Ting Chan[2,5], Li Quan Koh[1,2], Tsz Ying Yuen[2,4], Yee Hwee Lim[2], Charles W. Johannes[1,6], and Zachary P. Gates*[,1,2]

*Correspondence to: Zachary_Gates@imcb.a-star.edu.sg

[1]Institute of Molecular and Cell Biology (IMCB), Agency for Science, Technology and Research (A*STAR), 61 Biopolis Drive, Proteos, Singapore 138673, Republic of Singapore

[2]Institute of Sustainability for Chemicals, Energy and Environment (ISCE[2]), Agency for Science, Technology and Research (A*STAR), 8 Biomedical Grove, #07-01 Neuros, Singapore 138665, Republic of Singapore

[3]Current affiliation: Tri-Institutional PhD Program in Chemical Biology, Weill Cornell Medicine, The Rockefeller University, Memorial Sloan Kettering Cancer Center, New York, NY 10065, United States

[4]Current affiliation: Illumina, Inc., 7 North Coast Avenue, Singapore 737664

[5]Current affiliation: Department of Chemistry, The University of Chicago, Chicago, IL 60637, United States

[6]Current affiliation: FOG Pharmaceuticals, Inc., 30 Acorn Park Drive, Cambridge, MA 02140, United States

Table of Contents

Supplementary Figures 4

Experimental..20

‘Split-mix’ synthesis of ‘shotgun Ala-scan’ libraries 20

Solid phase extraction 23

Large-scale solid phase extraction..23

Small-scale solid phase extraction..23

Liquid chromatography-mass spectrometry analysis 24

Nano liquid chromatography-tandem mass spectrometry using Orbitrap Fusion Lumos................25

De novo sequencing and data refinement..27

Acetamide capping of KRpep(‘SH’)..28

Acetamide capping of KRpep(‘SH’) library 29

α,α’-Dibromo-*m*-xylene cyclization of KRpep(‘SH’) 30

α,α’-Dibromo-*m*-xylene cyclization of KRpep(‘SH’) library..31

PITC installation on KRpep(‘S-mxy-S’)..32

PITC installation on KRpep(‘S-mxy-S’) library..33

Linearization of PITC-KRpep(‘S-mxy-S’)..34

Linearization of PITC-KRpep(‘S-mxy-S’) library 35

Pentafluorophenyl sulfide cyclization of KRpep(‘SH’)..36

Pentafluorophenyl sulfide cyclization of KRpep(‘SH’) library..37

PITC installation on KRpep(‘S-pps-S’) 38

PITC installation on KRpep(‘S-pps-S’) library..39

Attempted linearization of PITC-KRpep(‘S-pps-S’)..40

Attempted linearization of PITC-KRpep(‘S-pps-S’) library..41

Attempted diiodomethane cyclization of KRpep(‘SH’) (original conditions)....................................42

Attempted diiodomethane cyclization of KRpep(‘SH’) (adapted mxy cyclisation conditions) 44

Attempted diiodomethane cyclization of KRpep(‘SH’) (A*STAR conditions)..................................46

Acetamide capping of Zpep(‘SH’)..48

Acetamide capping of Zpep(‘SH’) library..49

α,α’-Dibromo-*m*-xylene cyclization of Zpep(‘SH’) 50

α,α’-Dibromo-*m*-xylene cyclization of Zpep(‘SH’) library 51

PITC installation on Zpep(‘S-mxy-S’) 52

PITC installation on Zpep(‘S-mxy-S’) library..53

Edman linearization of PITC-Zpep(‘S-mxy-S’)..54

Edman linearization of PITC-Zpep(‘S-mxy-S’) library..55

Pentafluorophenyl sulfide cyclization of Zpep(‘SH’) 56

Pentafluorophenyl sulfide cyclization of Zpep(‘SH’) library..57

PITC installation on Zpep(‘S-pps-S’) 58

PITC installation on Zpep('S-pps-S') library 59
Attempted diiodomethane cyclization of Zpep('SH') (original conditions)..60
Attempted diiodomethane cyclization of Zpep('SH') (adapted mxy cyclisation conditions)..62
Attempted diiodomethane cyclization of Zpep('SH') (A*STAR conditions)..64
N-terminus acetylation of 'KRpep' peptidyl-resin..66
N-terminus acetylation of 'Zpep' peptidyl-resin..67
Attempted diiodomethane cyclization of Ac-KRpep('SH') (adapted mxy cyclisation conditions)......68
Attempted diiodomethane cyclization of Ac-Zpep('SH') (adapted mxy cyclisation conditions).........70
Diiodomethane cyclization of Ac-KRpep('SH') (A*STAR conditions) 72
Diiodomethane cyclization of Ac-Zpep('SH') (A*STAR conditions) 74
Acetamide capping of PMI('SH')..76
Acetamide capping of PMI('SH') library..77
α,α'-Dibromo-*m*-xylene cyclization of PMI('SH') 78
α,α'-Dibromo-*m*-xylene cyclization of PMI('SH') library 79
PITC installation on PMI('S-mxy-S') 80
PITC installation on PMI('S-mxy-S') library 81
Edman linearization of PITC-PMI('S-mxy-S') 82
Edman linearization of PITC-PMI('S-mxy-S') library..83

Supplementary Figures

A

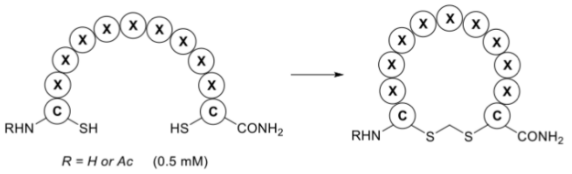

Original conditions: TCEP.HCl (0.75 mM), K_2CO_3 (1.5 mM), H_2O, 10 min; then Et_3N (2.5 mM), CH_2I_2 (1.5 mM), THF, rt, 18 h

Adapted **mxy** cyclization conditions: DTT (2 mM), CH_2I_2 (2.625 mM); ACN : 20 mM NH_4HCO_3 pH 7.8 buffer (1:7), degassed, rt, 18 h

A*STAR conditions: DTT (2 mM), CH_2I_2 (18 mM); ACN : 6 M GdmCl, 200 mM phosphate, pH 9 buffer (1:1), degassed, rt, 18 h

B

Condition	'KRpep'	'Zpep'	Ac-'KRpep'	Ac-'Zpep'
Original	╳	╳	—	—
Adapted **mxy**	╳	╳	╳	╳
A*STAR	╳	╳	◯	◯

— = not tested

╳ = product not present

◯ = product present

C **D**

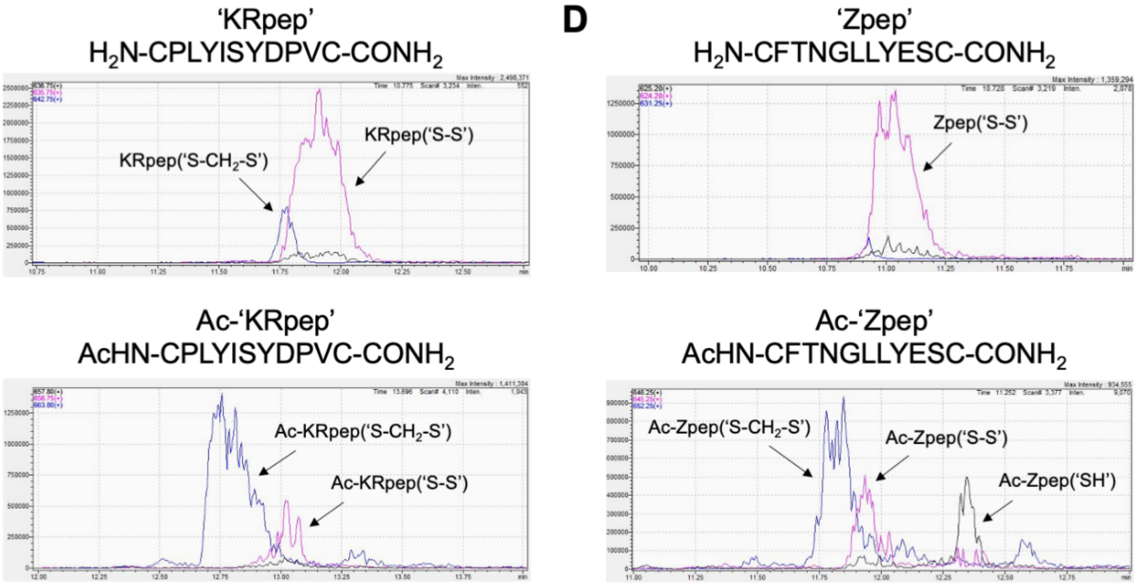

Figure S1. Reaction of KRpep('SH') and Zpep('SH') with diiodomethane did not result in any significant quantities of CH_2 macrocycle formed under all conditions tested. Only N-terminal acetylated peptides Ac-KRpep('SH') and Ac-Zpep('SH') yielded the CH_2 macrocycle as the major product under A*STAR conditions. **A)** Reaction scheme for the diiodomethane cyclization of KRpep('SH') and Zpep('SH') under original, adapted **mxy** cyclization, and A*STAR conditions. **B)** Outcomes of diiodomethane cyclization of stated peptides under specific conditions (╳ = unsuccessful; — = not tested; ◯ = partially successful). Extracted ion chromatogram traces of the reaction mixtures after reacting diiodomethane with **C)** KRpep('SH') (top) and Ac-KRpep('SH') (bottom); and **D)** Zpep('SH') (top) and Ac-Zpep('SH') (bottom) under A*STAR conditions.

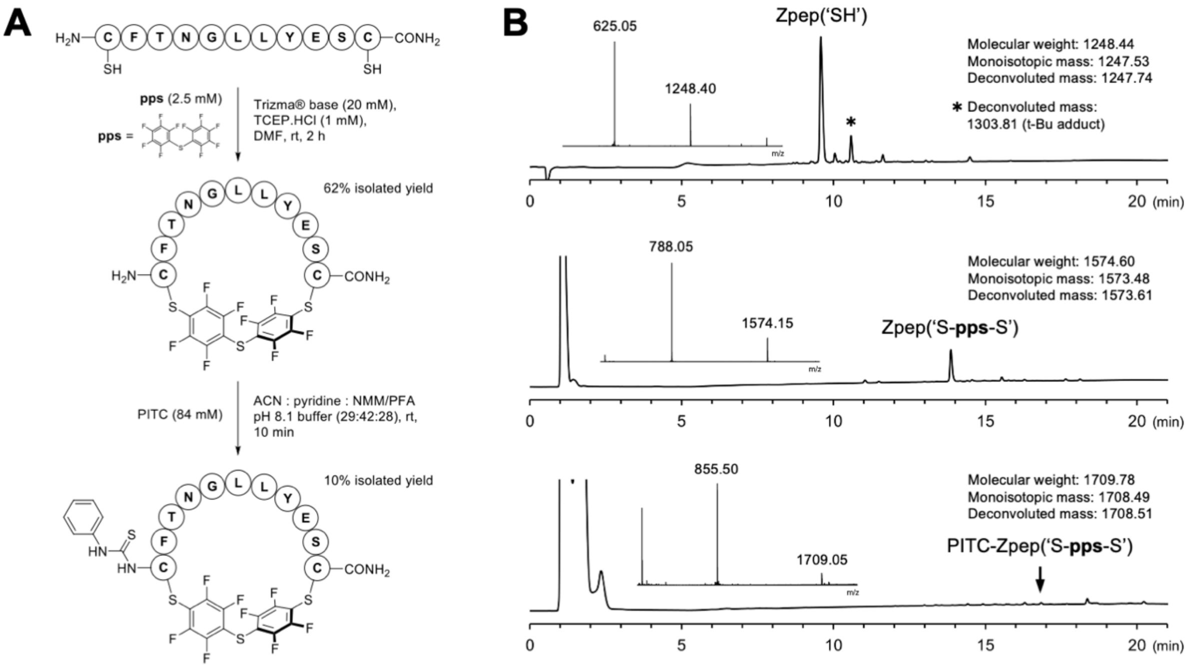

Figure S2. The model peptide Zpep('SH') successfully undergoes pps cyclization. However, PITC installation on Zpep('S-**pps**-S') was too low-yielding to proceed to conduct acid-catalyzed linearization of the product. **A)** Schematic of **pps** cyclization and subsequent PITC installation on Zpep('SH'). **B)** LC-MS analysis of the *bis*-Cys peptide starting material, and each of two reaction products. UV chromatograms are shown (equivalent y-axis range throughout; 5 µg nominal sample loadings), with inlaid mass spectra integrated across the principal UV component. Crude products were isolated by solid phase extraction after each step and used without further purification.

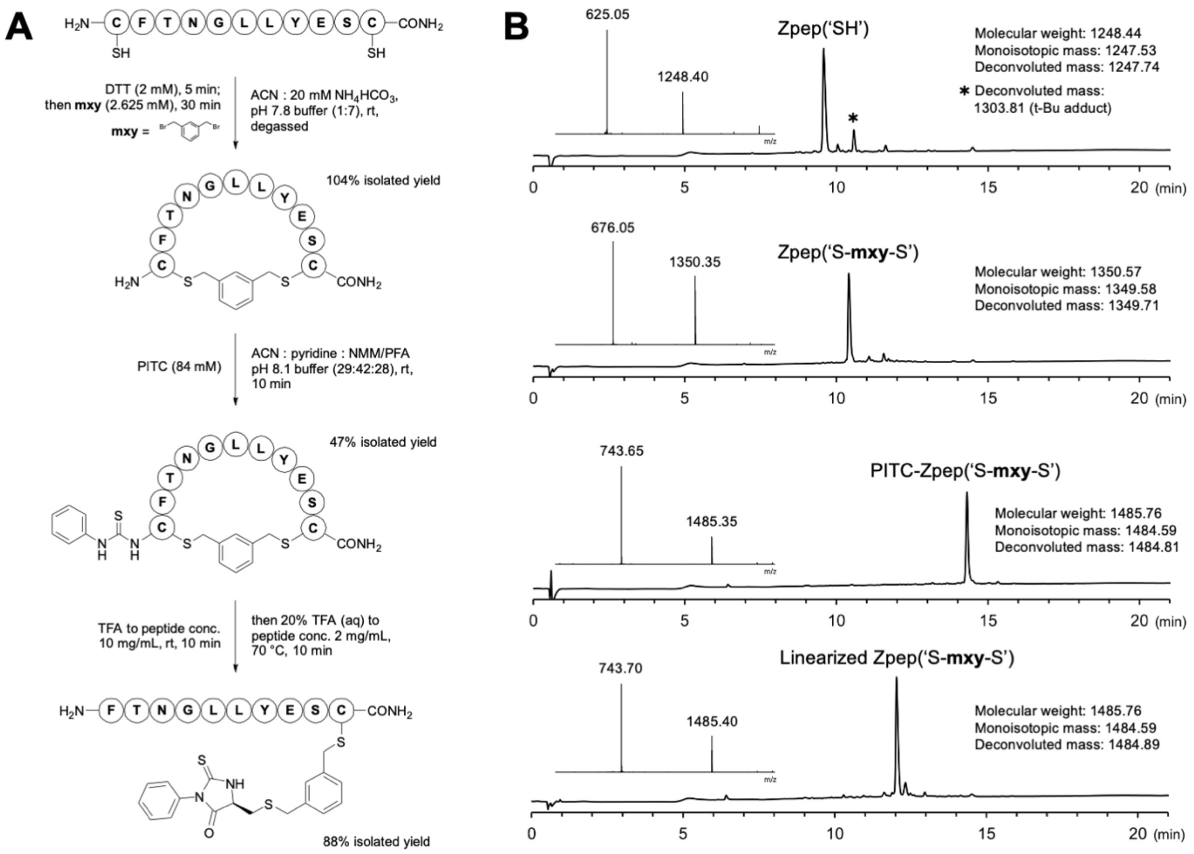

Figure S3. The model peptide Zpep('SH') undergoes clean conversion from *bis*-Cys peptide to linearized macrocycle product over a three-step macrocyclization/chemical linearization sequence. A) Schematic illustrating each of three steps in the reaction sequence. **B)** LC-MS analysis of the *bis*-Cys peptide starting material, and each of three reaction products. UV chromatograms are shown (equivalent y-axis range throughout; 5 µg nominal sample loadings), with inlaid mass spectra integrated across the principal UV component. Crude products were isolated by solid phase extraction after each step and used without further purification.

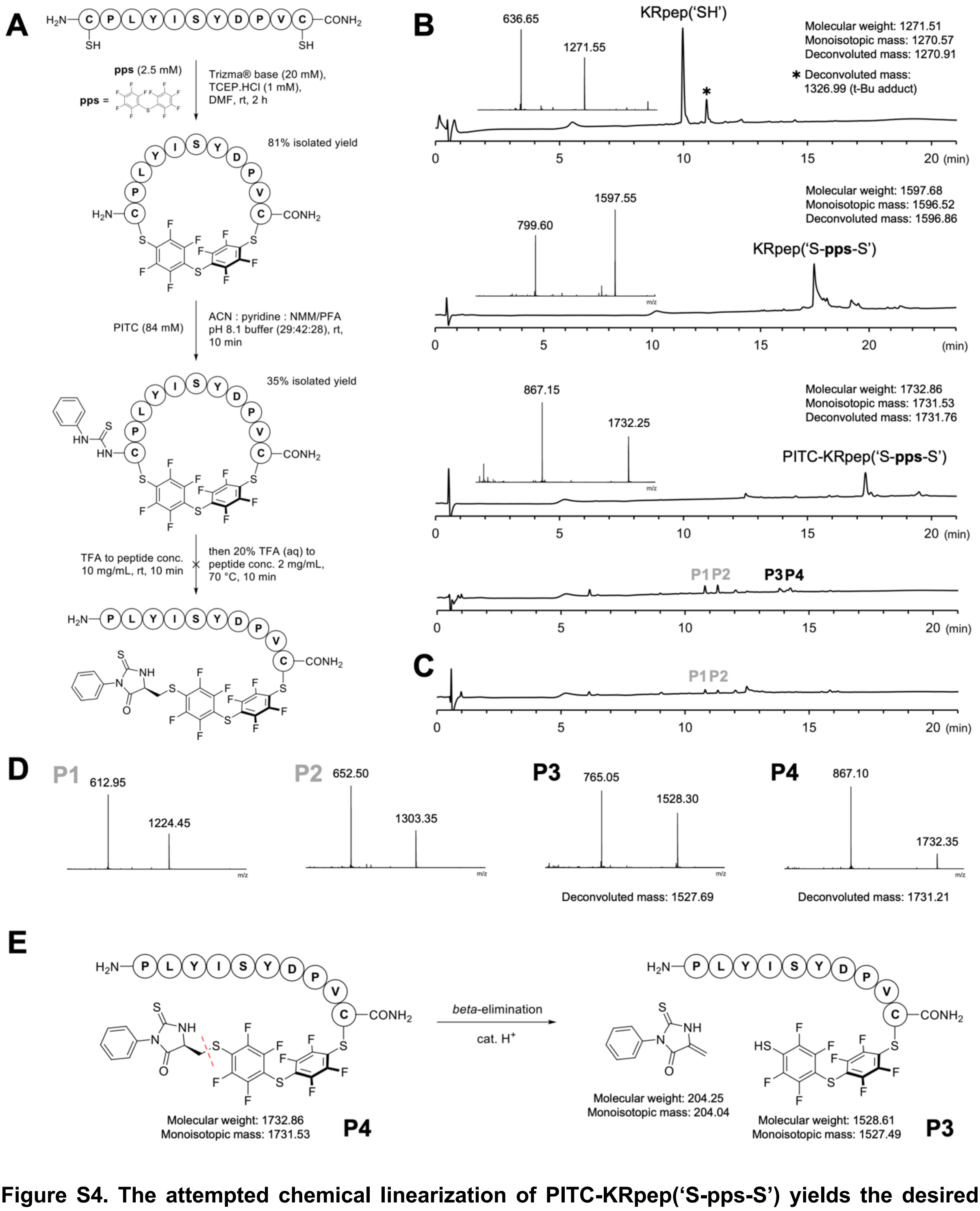

Figure S4. The attempted chemical linearization of PITC-KRpep('S-pps-S') yields the desired linearized macrocycle as a minor product. A) Schematic illustrating each of three steps in the macrocyclization/chemical linearization reaction sequence. **B)** LC-MS analysis of the *bis*-Cys peptide starting material, and each of three reaction products. UV chromatograms are shown (equivalent y-axis range throughout; 5 µg nominal sample loadings), with inlaid mass spectra integrated across the principal UV component. Crude products were isolated by solid phase extraction after each step and used without further purification. Peaks P1 and P2 could not be identified. **C)** UV chromatogram of the reaction product of a replicate of the chemical linearization step. **D)** Mass spectra integrated across each of the peaks P1-P4 in B). **E)** Proposed mechanism of fragmentation of linearized macrocycle (P4) to the side product (P3).

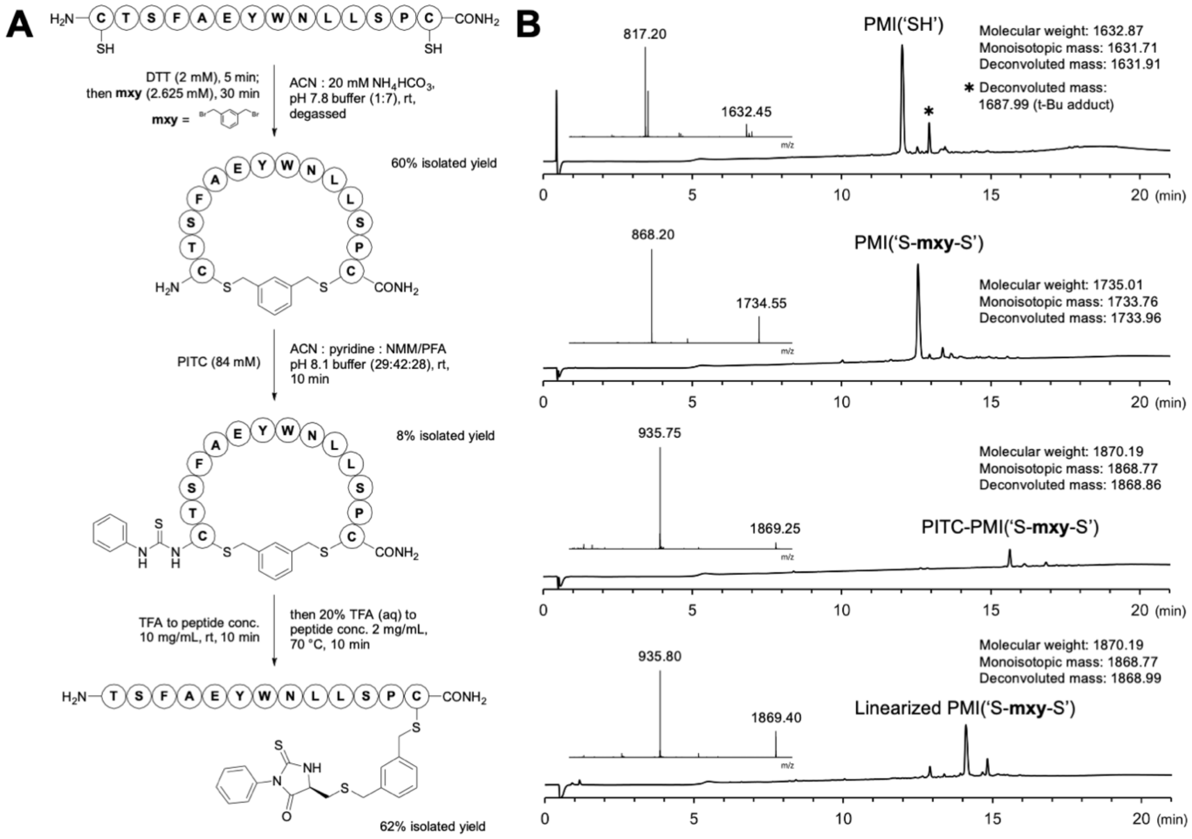

Figure S5. The model peptide PMI('SH') undergoes clean conversion from *bis*-Cys peptide to linearized macrocycle product over a three-step macrocyclization/chemical linearization sequence. A) Schematic illustrating each of three steps in the reaction sequence. **B)** LC-MS analysis of the *bis*-Cys peptide starting material, and each of three reaction products. UV chromatograms are shown (equivalent y-axis range throughout; 5 µg nominal sample loadings), with inlaid mass spectra integrated across the principal UV component. Crude products were isolated by solid phase extraction after each step and used without further purification.

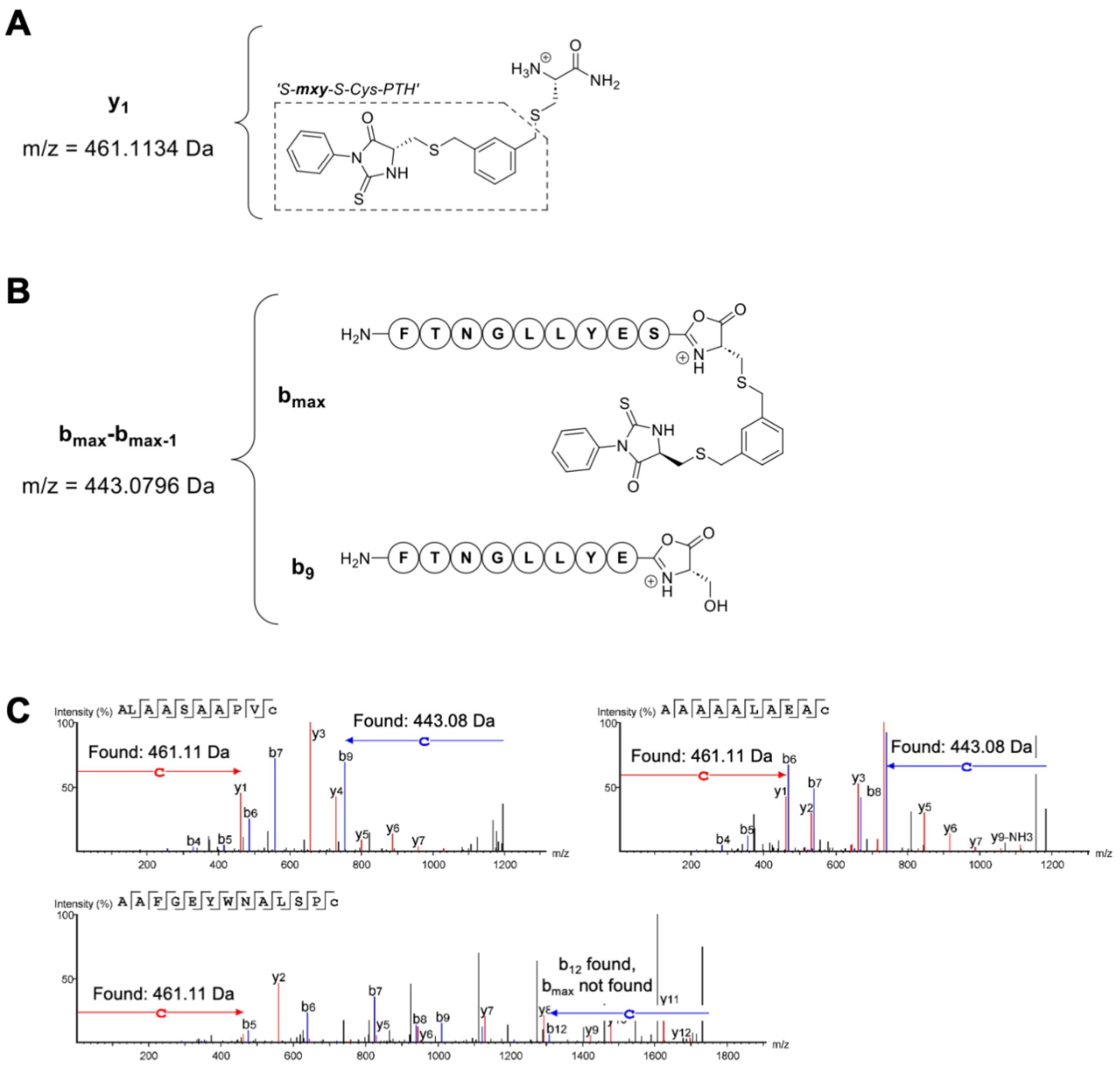

Figure S6. Diagnostic fragment ions and fragment ion mass differences support the assigned covalent structure of linearized macrocycles. A) and B) Structures of the y_1 and b_{max}/b_{max-1} fragment ions characteristic of linearized **mxy** macrocycles based on a Cys-(Xaa)$_n$-Cys scaffold. **C)** Representative MS^2 spectra of linearized **mxy** macrocycles from libraries based on 'KRpep' (upper left), 'Zpep' (upper right), and 'PMI' (below). Red arrows indicate the y_1 fragment ion; blue arrows indicate the mass difference between the b_{max} and b_{max-1} fragment ions. Here, 'c' corresponds to the modified Cys structure shown in A).

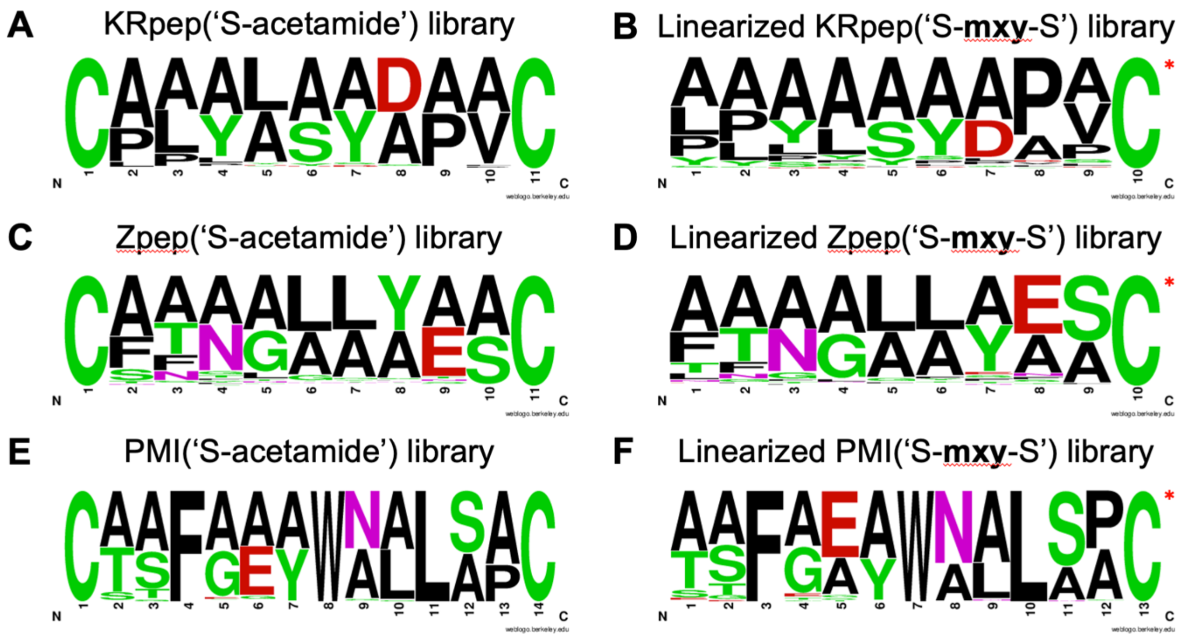

Figure S7. The N-termini of linearized KRpep('S-mxy-S') macrocycles are particularly poorly sequenced. Amino acid residue frequency plots for the *de novo* outputs of libraries based on: **A)** KRpep('S-acetamide'); **B)** linearized KRpep('S-**mxy**-S'); **C)** Zpep('S-acetamide'); **D)** linearized Zpep('S-**mxy**-S'); **E)** PMI('S-acetamide'); and **F)** linearized PMI('S-**mxy**-S'), and filtered for compliance with the set of amino acids used. LC-MS data were acquired at sample loadings of 100 fmol/peptide for A-E) and 200 fmol/peptide for F), in triplicate; precision and recall values were calculated as the average over three technical replicates. Here, 'C*' refers to the modified Cys structure shown in Figure S6.

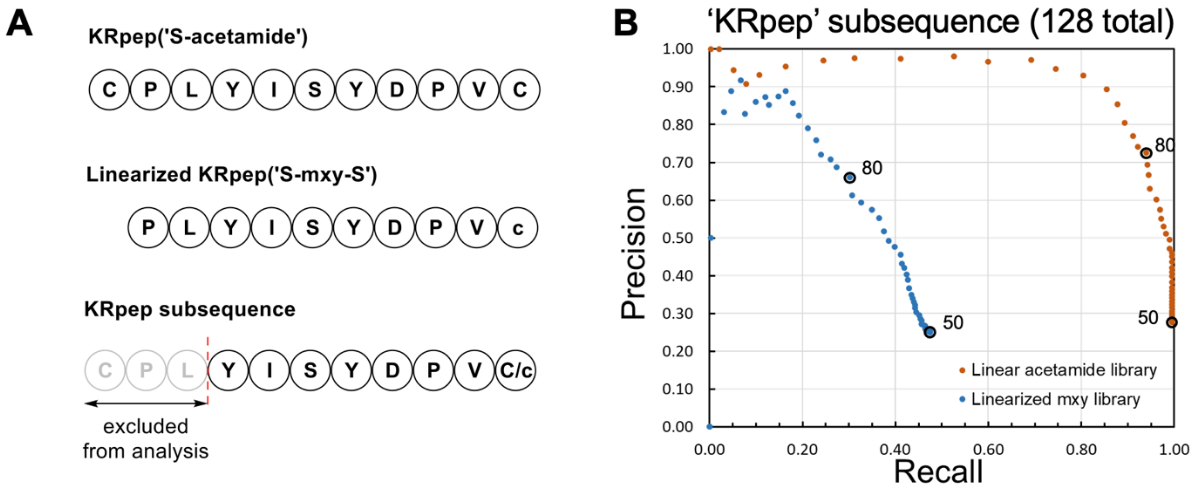

Figure S8. After accounting for the high proportion of misassigned dipeptides at the N-termini of linearized 'KRpep' macrocycles, linearized macrocycle and linear acetamide libraries yielded comparable precision across the range of score thresholds, similarly to 'Zpep' and 'PMI'. A) Schematic illustrating how sequencing data from the 'KRpep' libraries was re-analyzed, considering the C-terminal 8 residues only. **B)** Precision-recall curves for 'KRpep' C-terminal subsequences.

A

Method 1	Method 2
All filtered peptide sequences are considered	A subset of the filtered peptide sequences are considered: those whose mass implies a single sequence
Assignments are considered accurate if they match any library peptide	Assignments are considered accurate if they match the implied peptide

B

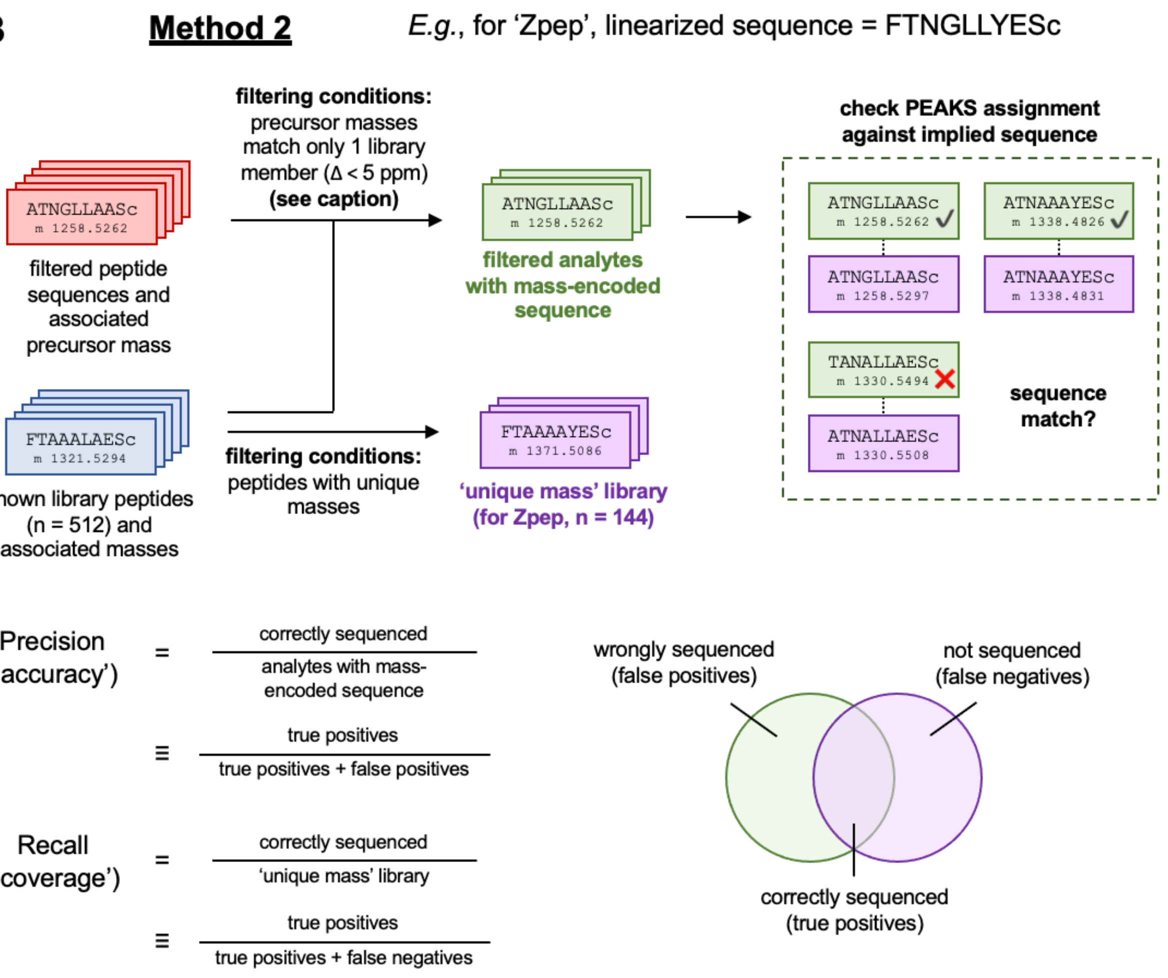

Figure S9. An orthogonal method to evaluate *de novo* sequencing, 'Method 2', was used to additionally confirm the findings based on 'Method 1'. A) A summary of differences between both methods. **B)** Schematic illustration of the data analysis workflow for Method 2, which was used to evaluate *de novo* sequence assignments by comparison to the implied peptide.

Note: The calculated precursor masses associated with the filtered peptide sequences were searched against all known library peptides. By selecting analytes with masses that match 1 library peptide only, this procedure necessarily returns matches from the 'unique mass' library.

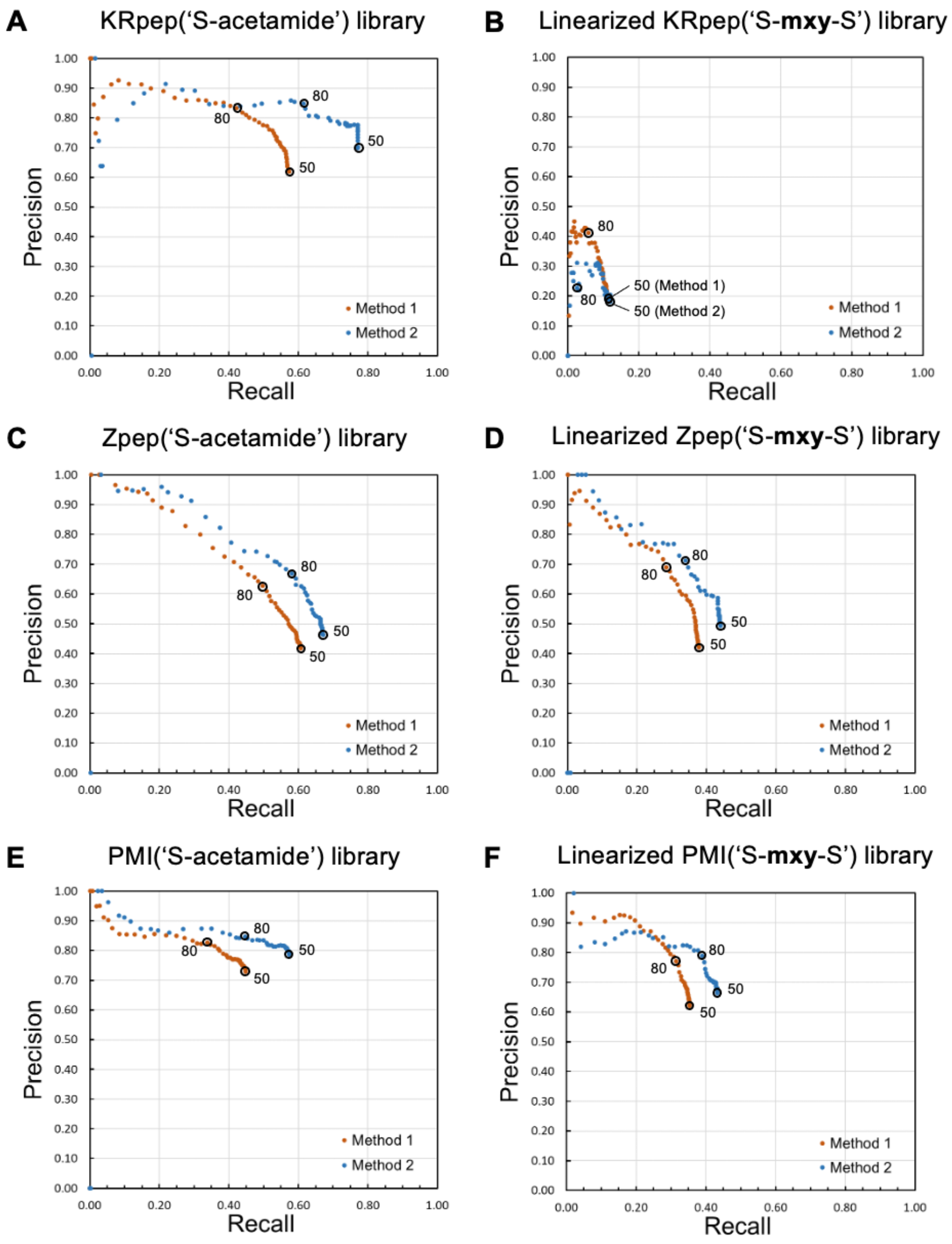

Figure S10. The orthogonal 'Method 2' analysis of *de novo* sequencing outputs corroborates findings based on 'Method 1'. For each library, the precision-recall curves generated based on the two methods were broadly similar. Precision-recall curves from 'Method 1' (orange) and 'Method 2' (blue) for libraries based on: **A)** KRpep('S-acetamide'); **B)** linearized KRpep('S-**mxy**-S'); **C)** Zpep('S-acetamide'); **D)** linearized Zpep('S-**mxy**-S'); **E)** PMI('S-acetamide'); and **F)** linearized PMI('S-**mxy**-S'). LC-MS data were acquired at sample loadings of 100 fmol/peptide for **A-E)** and 200 fmol/peptide for **F)**, in triplicate; precision and recall values were calculated as the average over three technical replicates.

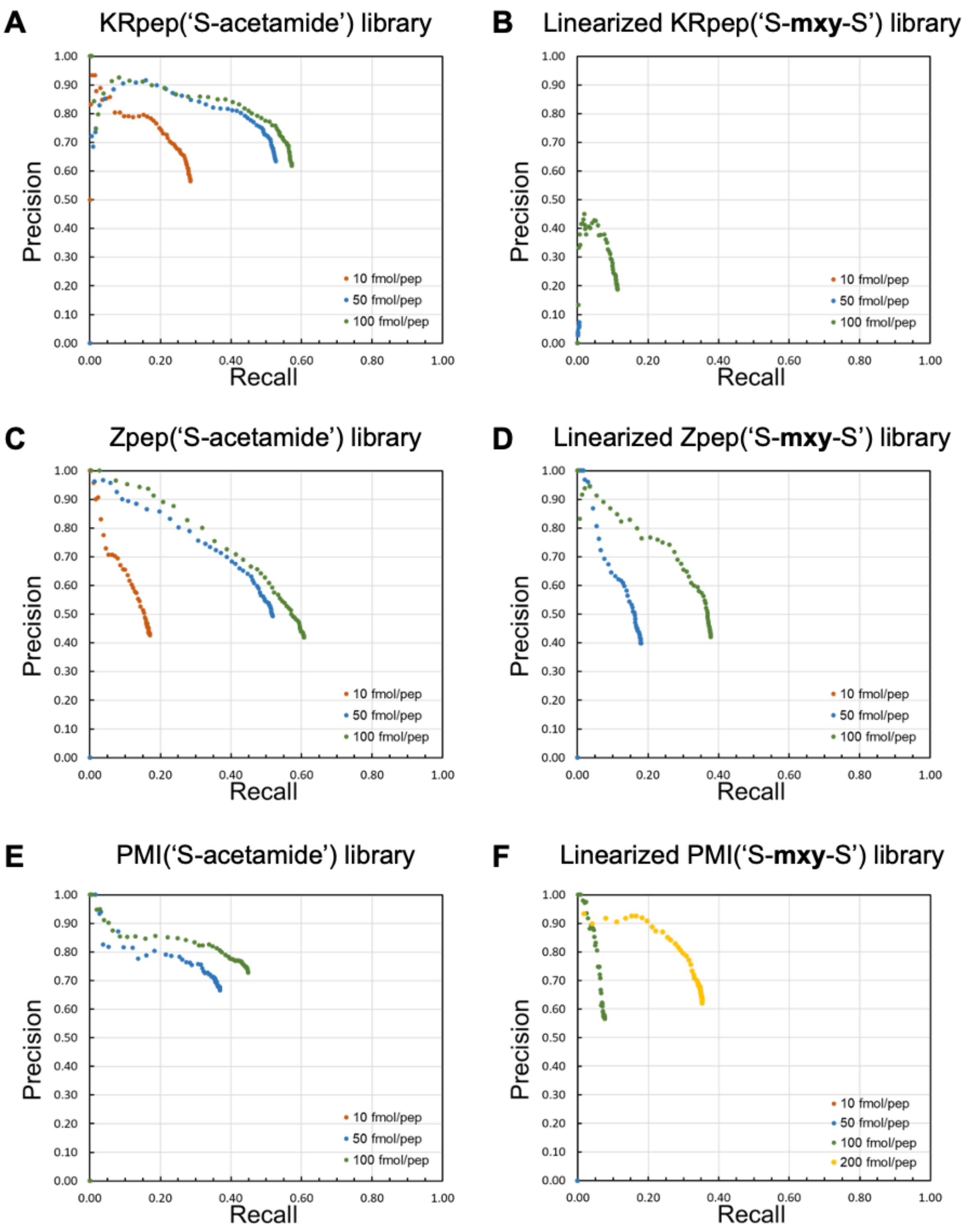

Figure S11. Increasing nLC-MS/MS sample loading leads to improved recall. Precision-recall curves at different sample loadings of 10, 50, and 100 fmol/peptide for libraries based on: **A)** KRpep('S-acetamide'); **B)** linearized KRpep('S-**mxy**-S'); **C)** Zpep('S-acetamide'); **D)** linearized Zpep('S-**mxy**-S'); **E)** PMI('S-acetamide'); and **F)** linearized PMI('S-**mxy**-S'), which also included a 200 fmol/peptide loading. Sample loadings were calculated by dividing the quantity of total peptide by the library diversity (512 peptides). LC-MS data was acquired in triplicate; precision and recall values were calculated as the average over three technical replicates, except for the 10 fmol/peptide loading for **E)** and 10 & 50 fmol/peptide loadings for **F)**, for which no replicates were conducted.

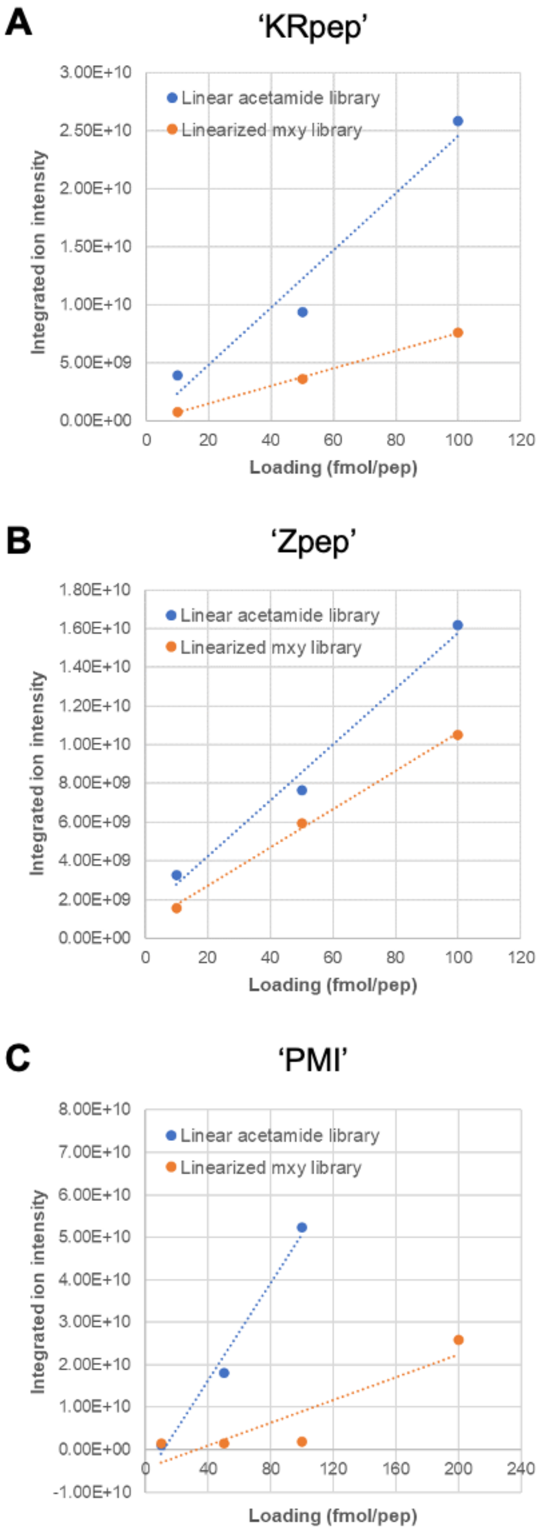

Figure S12. Integrated ion intensity increases with (nominal) peptide loading, and linearized mxy libraries have consistently lower ion intensities than corresponding linear acetamide libraries at each (nominal) peptide loading. Graphs illustrating how integrated ion intensities vary across different loadings for linear acetamide (blue) and linearized **mxy** (orange) libraries based on **A)** 'KRpep'; **B)** 'Zpep'; and **C)** 'PMI'. Integrated ion intensities were determined by integration of EIC chromatograms on the mass range of each library, over the range of retention times where filtered peptides were detected.

A

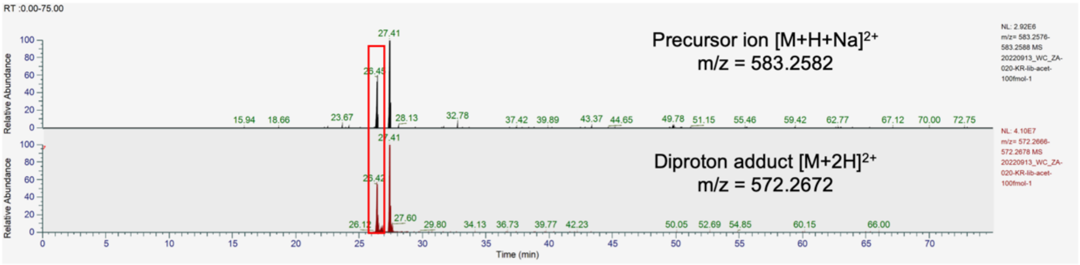

B

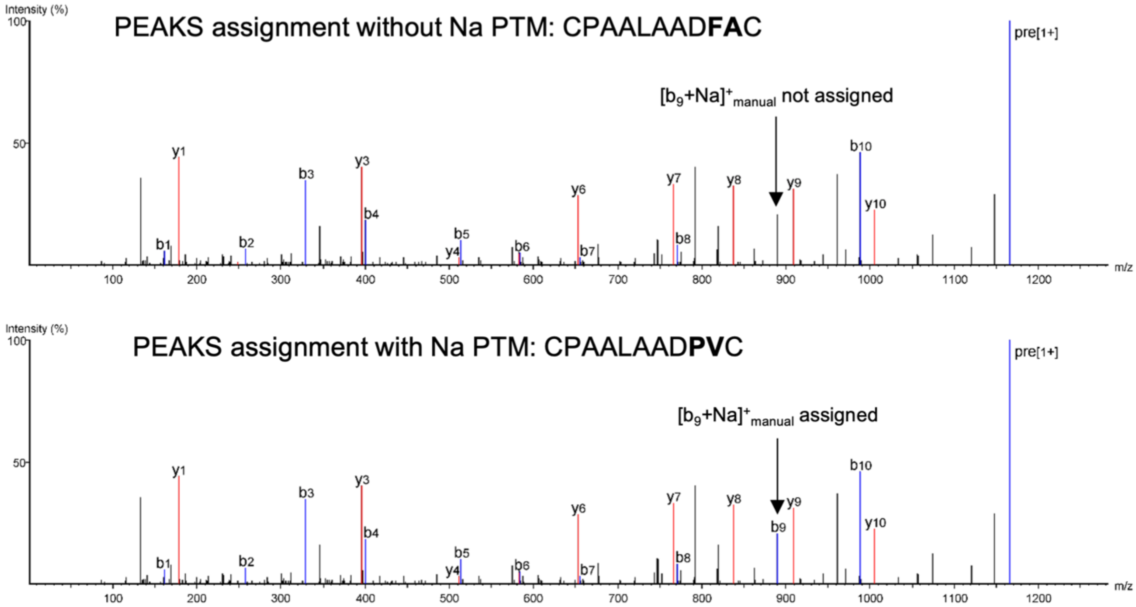

C

Manual assignment						
CPAALAADPVC						
	$[b+H]^+$	$[b+Na]^+$	Seq.	$[y+H]^+$	$[y+Na]^+$	
1	161.04	183.02	C(+57.02)	1143.53	1165.51	*11*
2	258.09	280.07	P	983.50	1005.48	*10*
3	329.13	351.11	A	886.45	908.43	*9*
4	400.16	422.15	A	815.41	837.39	*8*
5	513.25	535.23	L	744.37	766.36	*7*
6	584.28	606.27	A	631.29	653.27	*6*
7	655.32	677.30	A	560.25	582.23	*5*
8	770.35	792.33	D	489.22	511.20	*4*
9	867.40	889.38	P	374.19	396.17	*3*
10	966.47	988.45	V	277.14	299.12	*2*
11	1125.52	1147.50	C(+56.04)	178.07	200.05	*1*

PEAKS assignment w/o optional Na PTM				
CPAALAADFAC				
	$[b+H]^+$	Seq.	$[y+H]^+$	
1	161.04	C(+57.02)	1165.51	*11*
2	258.09	P	1005.48	*10*
3	329.13	A	908.43	*9*
4	400.16	A	837.39	*8*
5	513.25	L	766.36	*7*
6	584.28	A	653.27	*6*
7	655.32	A	582.24	*5*
8	770.35	D	511.20	*4*
9	917.42	F	396.17	*3*
10	988.45	A	249.10	*2*
11	1147.50	C(+56.04)	178.07	*1*

PEAKS assignment with optional Na PTM				
CPAALAADP(+Na-H)VC				
	$[b+H]^+$	Seq.	$[y+H]^+$	
1	161.04	C(+57.02)	1165.51	*11*
2	258.09	P	1005.48	*10*
3	329.13	A	908.43	*9*
4	400.16	A	837.39	*8*
5	513.25	L	766.36	*7*
6	584.28	A	653.27	*6*
7	655.32	A	582.23	*5*
8	770.35	D	511.20	*4*
9	889.38	P(+21.98)	396.17	*3*
10	988.45	V	277.14	*2*
11	1147.50	C(+56.04)	178.07	*1*

Figure S13. Incorporating an optional Na post-translational modification during *de novo* peptide sequencing enables accurate sequencing of Na adducts. A) Extracted ion chromatograms, showing the co-elution of $[M+2H]^{2+}$ and $[M+Na+H]^{2+}$ precursor ions of a KRpep('S-acetamide') peptide (red box). **B)** The MS^2 spectrum collected on the sodium adduct from A), with peaks assigned to fragment ions with (below) or without (above) considering an optional Na PTM. **C)** Fragment ion tables for the manual assignment (left), as well as the PEAKS assignments without (center) and with (right) the Na PTM, to the example spectrum. Fragment ion masses shown in grey were absent. When an optional Na PTM is allowed, the $[b_9+Na]^+$ fragment ion (m/z 889.38) can be assigned, allowing 'CPAALAADFAC' and 'CPAALAADP(+Na-H)VC' to be distinguished despite the near-isobaric ambiguities arising from 'Phe+Ala' ~ 'Pro+Val+Na-H'. Where the ion ladder 'crosses over' between proton and Na adducts in the PEAKS assignments ion tables, this is denoted by a change in shade, in accordance with the manual assignment ion table.

KRpep('S-acetamide') library (CPLYISYDPVC)

PEAKS assignment (score)	Probable Na adduct?	Non-library precursor mass?	Pure 'Inversion'?	Pure 'Substitution'?	Combined Inversion/ Substitution
CPAALAADFAC (98)	✓				
CALALSAAFAC (97)	✓				
CPAYASAAFAC (94)	✓				
CPLYLAAAFAC (97)	✓	✓			
CALALAYAFAC (96)	✓	✓			
CAAYLAAAFAC (96)	✓	✓			
CAADEDEDPAC (96)		✓			
CLAALAADAVC (97)			✓		
CAAYLAADFAC (96)				✓	
CPAAAYSDFAC (97)					✓

Linearized KRpep('S-**mxy**-S') library (PLYISYDPVC)

PEAKS assignment (score)	Probable Na adduct?	Non-library precursor mass?	Pure 'Inversion'?	Pure 'Substitution'?	Combined Inversion/ Substitution
ALALSAAAFC (96)	✓				
LAAAAYAPAC (96)	✓				
PAYAAAAFAC (97)	✓	✓			
PAYASYAFFW (98)		✓			
PAAASADFFW (96)		✓			
PLYASADWFF (96)		✓			
LAALSAAPVC (97)			✓		
LAALSAAPAC (96)			✓		
LAALSADPAC (96)			✓		
LAALSADPVC (96)			✓		

Zpep('S-acetamide') library (CFTNGLLYESC)

PEAKS assignment (score)	Probable Na adduct?	Non-library precursor mass?	Pure 'Inversion'?	Pure 'Substitution'?	Combined Inversion/ Substitution
CFANAATYASC (97)	✓				
CFANALTYAAC (97)	✓				
CAANYLAYASC (96)	✓				
CFAAGLTAYAC (93)	✓				
CSNNGLLAASC (96)		✓			
CTFAAAAAESC (95)			✓		
CTAAALAYESC (94)			✓		
CAEYLLGACSN (93)			✓		
CTANGLAAESC (93)			✓		
CTFAALAYASC (93)			✓		

Linearized Zpep('S-**mxy**-S') library (FTNGLLYESC)

PEAKS assignment (score)	Probable Na adduct?	Non-library precursor mass?	Pure 'Inversion'?	Pure 'Substitution'?	Combined Inversion/ Substitution
FANGLTYAAC (95)	✓				
FANAATYASC (93)	✓				
FANALTYSAC (91)	✓				
ATQALYEEEP (95)		✓			
FTNALGYMAC (94)		✓			
TAAAAAEEEP (92)		✓			
AAAGAAFASC (93)			✓		
AFAGAAYESC (93)			✓		
AFAALAAESC (92)			✓		
AANALLYACS (92)			✓		
**Total: 40**	**Total: 16**	**Total: 12** (8 of which cannot be attributed to Na adducts)	**Total: 14**	**Total: 1**	**Total: 1**

Figure S14. Summary of high-scoring misassignments from each four libraries (see main text, 'Common errors in *de novo* assignments and role of sodium (Na) adducts'). Amino acid assignments not possible according to the library design are shown in red. 'Probable Na adducts' were deduced based on their co-elution with an analyte of 21.98 Da lighter mass (determined by inspection of extracted ion chromatograms).

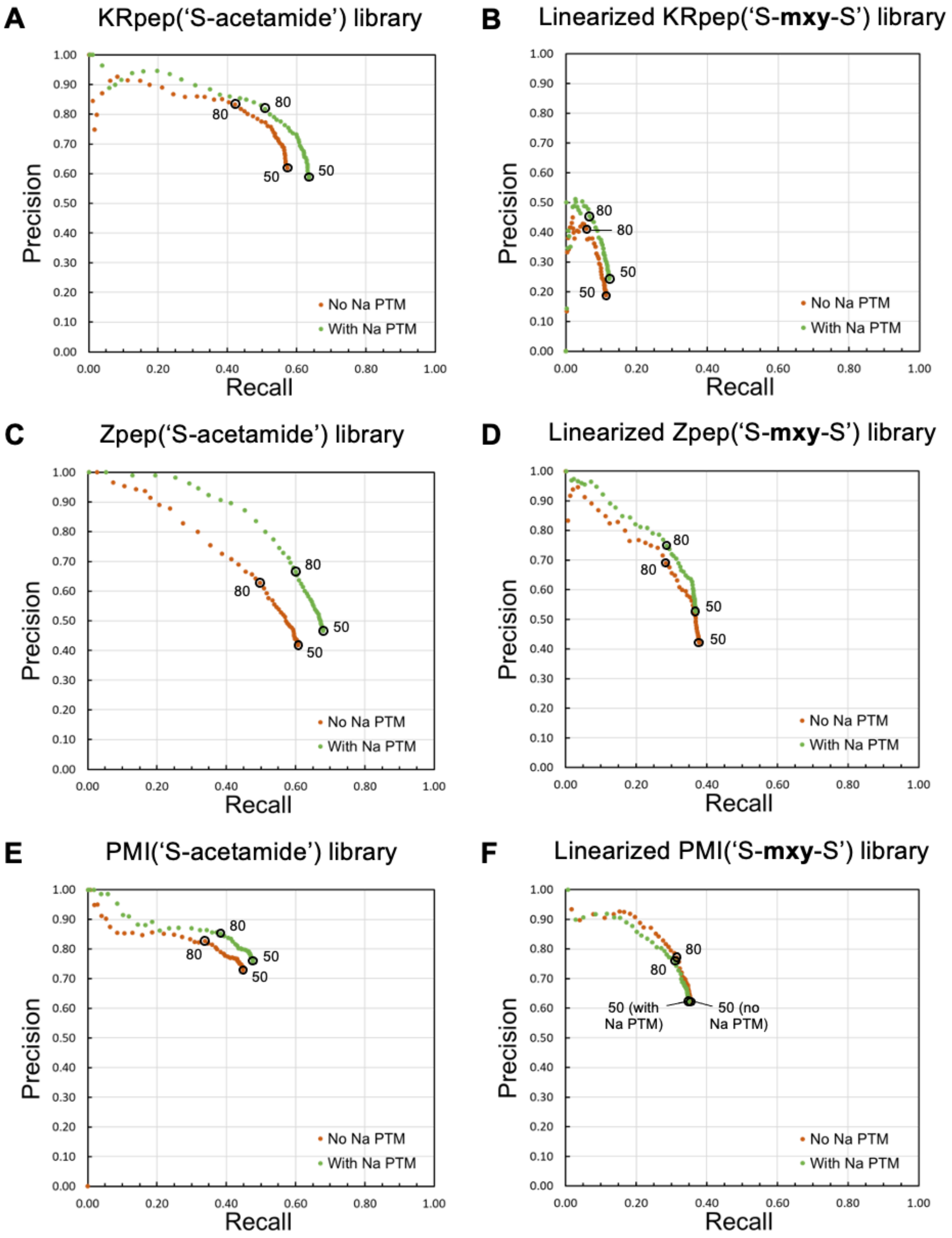

Figure S15. The inclusion of an optional Na post-translational modification in *de novo* sequencing generally improves precision and/or recall. The 'Na PTM' definition was as follows: mass = 21.9819; variable modification at any residue type ('@[X]'). Precision-recall curves for the libraries based on: **A)** KRpep('S-acetamide'); **B)** linearized KRpep('S-**mxy**-S'); **C)** Zpep('S-acetamide'); **D)** linearized Zpep('S-**mxy**-S'); **E)** PMI('S-acetamide'); and **F)** linearized PMI('S-**mxy**-S'), with (green) or without (orange) the incorporation of an optional Na PTM during *de novo* sequencing. LC-MS data were acquired at sample loadings of 100 fmol/peptide for **A-E)** and 200 fmol/peptide for **F)**, in triplicate; precision and recall values were calculated as the average over three technical replicates.

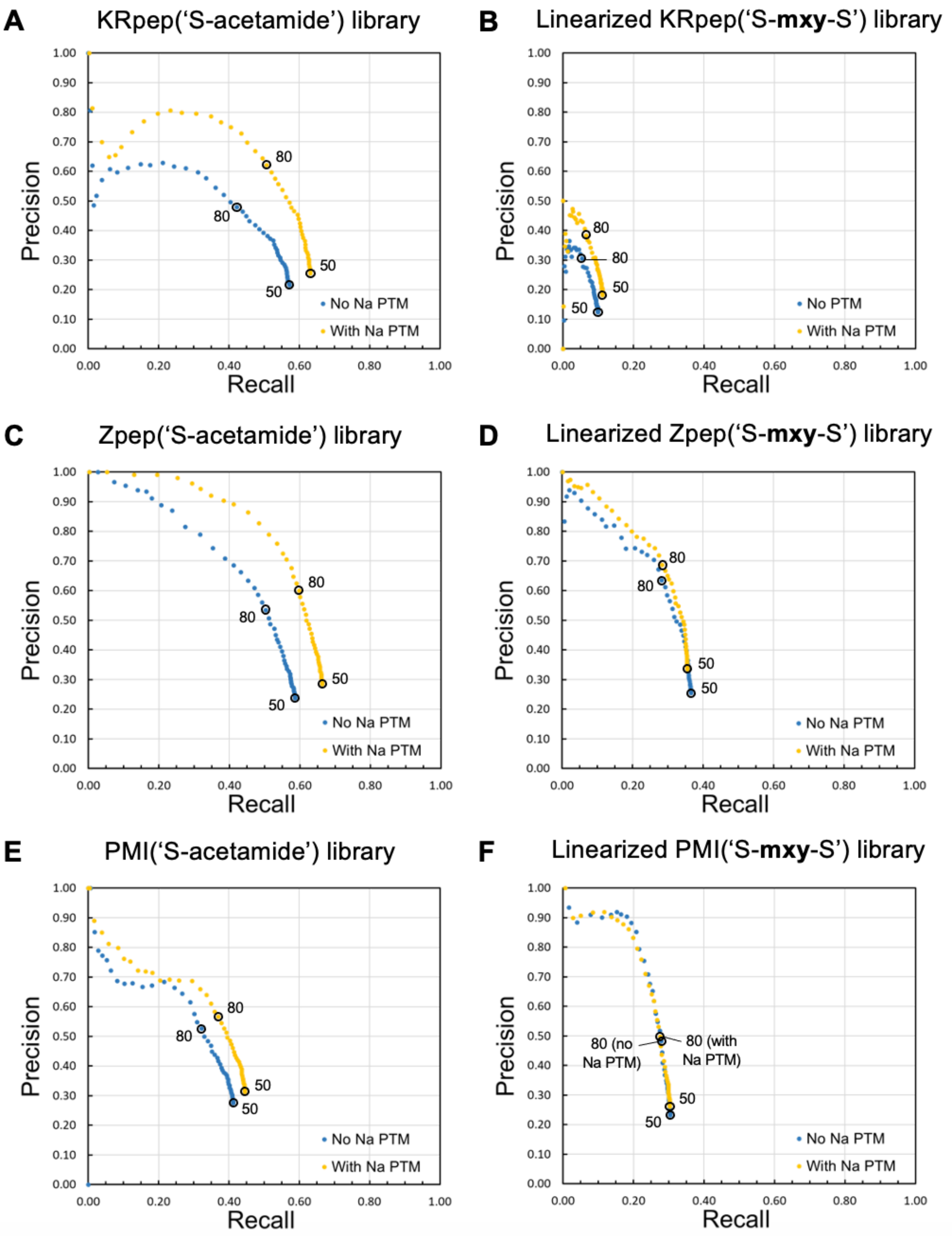

Figure S16. In some cases (*e.g.* 'KRpep('S-acetamide') library), the inclusion of an optional Na post-translational modification has more significant effects when filtration of raw *de novo* output is relaxed prior to calculating precision-recall. The analysis was as above (**Figure S15**), except that in this case, raw *de novo* output was filtered based on correct length and terminal residues only. This allowed peptides containing amino acids not present in the library to be included in the precision-recall calculations.

Experimental
'Split-mix' synthesis of 'shotgun Ala-scan' libraries

Library 1
'Wild type' sequence: CPLYISYDPVC-*CONH*$_2$ 'fixed position' residues are underlined; all others are randomized as WT or Ala
Average MW = 1,067.27
Average E_{280} = 1,520 (M^{-1} cm^{-1})

Library 2
'Wild type' sequence: CFTNGLLYESC-*CONH*$_2$
Average MW = 1,055.72
Average E_{280} = 880 (M^{-1} cm^{-1})

Library 3
'Wild type' sequence: CTSFAEYWNLLSPC-*CONH*$_2$
Note: The Ala residue was varied as either Ala, or Gly
Average MW = 1455.67
Average E_{280} = 6,245 (M^{-1} cm^{-1})

These procedures are reproduced from Koh, L. Q., Lim, Y. W., & Gates, Z. P. (2022). Affinity Selection from Synthetic Peptide Libraries Enabled by De Novo MS/MS Sequencing. *International Journal of Peptide Research and Therapeutics*, *28*(2), 1–14.

Installation of 'fixed position' residues
For each library, Tentagel S NH_2 90 µm beads (1 g, 0.25 mmol/g, 2.9 x 10^6 beads) were transferred to 20 mL fritted plastic syringes (Torviq) and swollen in DMF. 'Fmoc-Rink-COOH' (2 mmol) was dissolved in HATU solution (0.38 M in DMF, 5 mL, 1.9 mmol HATU), and treated with diisopropylethyl amine (520 µL, 3 mmol). The resulting solution was added to the resin. After 10 min, the resin was washed with DMF (3 x 5 mL flow washes). The Fmoc group was removed by treatment with 20% piperidine in DMF (1 x 5 mL flow wash; 2 x 5 mL batch treatments, 3 min each). Finally, the resin was washed with DMF (5 x 5 mL flow washes). Finally, each library was divided evenly between two 20 mL fritted syringes (Torviq).

Installation of 'randomized position' residues
The 'split' H_2N-peptidyl-resin portions were treated with solutions of either Fmoc-Ala-OH, or the appropriate 'wild type' Fmoc-AA-OH (1 mmol portions), activated as above. After 10 min, the resin portions were washed with DMF (as above), and 'pooled' by pouring into a 50 mL plastic tube (washing the fritted syringes with DMF to achieve quantitative transfer of the resin). The suspension was vigorously swirled, and then 'split' by dividing evenly between the two 20 mL fritted syringes (using a 1 mL Eppendorf micropipette). Fmoc group removal was performed as above, and the process was repeated for each varied position.

Preparative cleavage/side chain deprotection
Peptidyl-resin was washed twice with dichloromethane, drained under gravity and allowed to dry under a stream of nitrogen. The peptidyl-resin was cleaved using a 'cleavage cocktail' (95% trifluoroacetic acid, 2.5% water, 2.5% triisopropylsilane; 1 mL cleavage cocktail/75 mg of peptidyl-resin) for 2 hours at ambient temperature. The cleavage mixture was concentrated using inert gas and triturated with cold diethyl ether. The triturated mixture was clarified by centrifugation, discarding the solvent. The trituration process was repeated, and the resulting resin-precipitate residue was dried under inert gas, dissolved in 50% acetonitrile : 50% water (0.1% trifluoroacetic acid), and filtered using a 0.45 µm nylon filter. The filtrate was flash frozen and lyophilized to afford:

- KRpep('SH') as a white solid (49.24 mg, 38.73 μmol, 68% yield from 0.3 g peptidyl-resin)
- KRpep('SH') library as a white solid (88.67 mg, 83.04 μmol total; 162 nmol/peptide nominal, 75% yield from 0.56 g peptidyl-resin)
- Zpep('SH') as a white solid (32.24 mg, 25.82 μmol, 68% yield from 0.2 g peptidyl-resin)
- Zpep('SH') library as a white solid (46.70 mg, 44.21 μmol total; 86.3 nmol/peptide nominal, 56% yield from 0.4 g peptidyl-resin)
- PMI('SH') as a white solid (17.2 mg, 10.53 μmol, 74% yield from 0.1 g peptidyl-resin)
- PMI('SH') library as a white solid (24.32 mg, 16.61 μmol, 32.4 nmol/peptide nominal, 55% yield from 0.2 g peptidyl-resin)

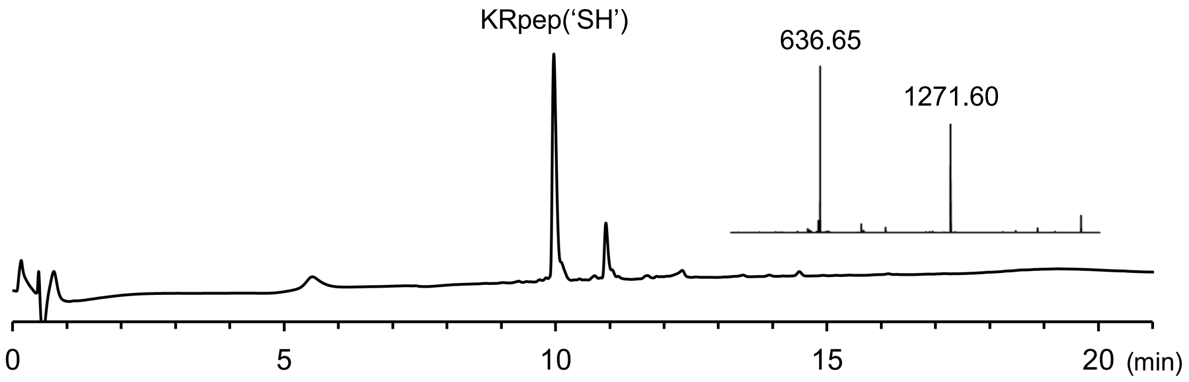

Figure S17. LC-MS analysis of KRpep('SH'). UV chromatogram is shown, with inlaid mass spectrum integrated across the principal UV component. Monoisotopic *m/z* calculated for $[M+H]^+$ 1271.57, found 1271.60.

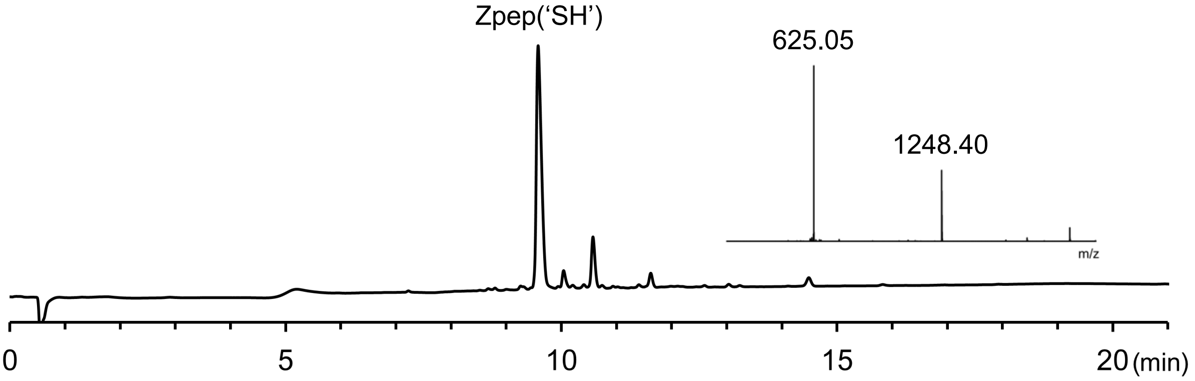

Figure S18. LC-MS analysis of Zpep('SH'). UV chromatogram is shown, with inlaid mass spectrum integrated across the principal UV component. Monoisotopic *m/z* calculated for $[M+H]^+$ 1248.53, found 1248.40.

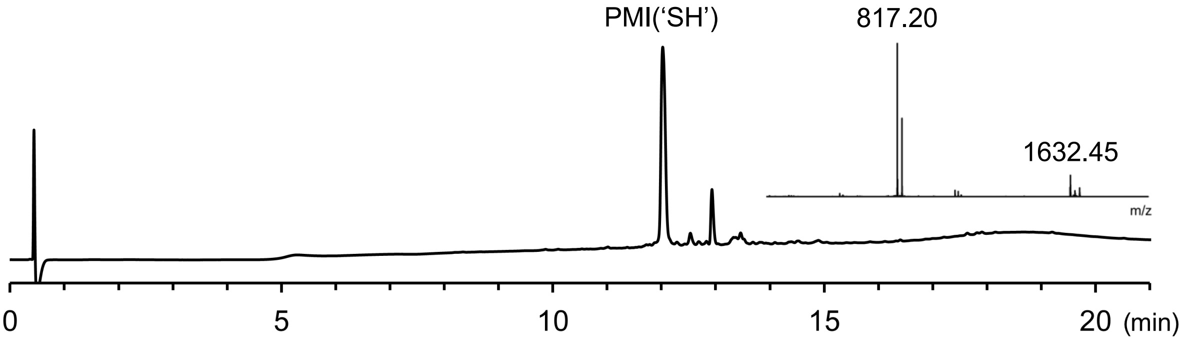

Figure S19. LC-MS analysis of PMI('SH'). UV chromatogram is shown, with inlaid mass spectrum integrated across the principal UV component. Monoisotopic *m/z* calculated for $[M+H]^+$ 1632.71, found 1632.45.

Solid phase extraction

Solvent A = MS grade water (0.1% trifluoroacetic acid)
Solvent B = MS grade acetonitrile (0.1% trifluoroacetic acid)

Large-scale solid phase extraction

Cartridge: S*Pure Maxi-Clean™ 300 mg C_{18} cartridge

1) Wetting the bonded phase, with Solvent B (5 mL)
2) Equilibrating, with Solvent A (5 mL)
3) Loading the reaction mixture
4) Washing, with Solvent A (5 mL)
5) Eluting, with 50/50 Solvent A/Solvent B (2 x 2 mL)

Small-scale solid phase extraction

Cartridge: MoBiTec Mobicol ‘F’ columns packed with Waters Oasis HLB resin (~30 mg, 30 µm)

1) Wetting the bonded phase, with Solvent B (0.2 mL)
2) Equilibrating, with Solvent A (5 x 0.2 mL)
3) Loading the reaction mixture
4) Washing, with Solvent A (5 x 0.2 mL)
5) Eluting, with 30/70 Solvent A/Solvent B (2 x 0.2 mL)

Liquid chromatography-mass spectrometry analysis

Mass spectra were acquired in positive mode using a Shimadzu LCMS-2020 single quadrupole liquid chromatography-mass spectrometry system, equipped with a Phenomenex Jupiter Proteo C_{12} (2.1 × 50 mm; 3.6 µm packing, for 'KRpep' and 'Zpep') or Phenomenex Aeris XB-C18 100Å (2.6 × 50 mm; 2.6 µm packing, for 'PMI'. Solvent A was MS grade water (0.1% trifluoroacetic acid); Solvent B was MS grade acetonitrile (0.1% trifluoroacetic acid). Chromatographic conditions were as follows:

Flow rate: 0.4 mL/min
Column oven: 25 °C
Gradient program (linear):

Time (min)	Solvent B (%)
0	1
3	1
18	61
21	61
21.01	1
24	1

Nano liquid chromatography-tandem mass spectrometry using Orbitrap Fusion Lumos

nLC parameters
LC system: ThermoFisher Easy-nLC 1200
Column: ThermoFisher Pepmap RSLC C18 (2 µm, 100 Å, 75 µm x 50 cm)
Column temperature: 40 °C
Trap/desalt column: not present
Flow rate: 300 nL/min
Sample loading volume: 5 µL

Mobile Phase A = MS grade water (0.05% acetic acid)
Mobile Phase B = 95% MS grade acetonitrile, 5% MS grade water (0.05% acetic acid)

'Standard run' gradient (linear steps)

Time (min)	Solvent B (%)
0	5
60	45
68	95
75	95

'Extended run' gradient (linear steps)

Time (min)	Solvent B (%)
0	5
90	65
98	95
105	95

Orbitrap Mass Spectrometry Parameters
Primary MS Spectra acquisition parameters

Primary MS Detection	Orbitrap
Orbitrap Resolution	120,000
Scan Range	200 – 1400
RF lens (%)	55
Normalized AGC Target	250%
Maximum Injection Time	100 ms
Microscans	1
Data type	Centroid
Polarity	Positive

Precursor selection parameters (data-dependent acquisition)

Dynamic Precursor Exclusion	On
Monoisotopic peak determination	Peptide
Charge States	2-6
Intensity Threshold	5×10^4
Dynamic Exclusion	Exclude precursors within a mass tolerance of 10 ppm for 30 s; if detected > 4 times within 30 s.<br>Exclude Isotopes<br>Dependent scan on single charge state per precursor

	Exclude within cycle
Data dependent mode	Number of Scans
Number of dependent scan	15

Selected precursors were subjected to CID and HCD:

Collision-induced dissociation (CID) (ddMS^2 OT CID)

Isolation Mode	Quadrupole
Isolation window (m/z)	1.3
Isolation offset	Off
Activation Type	CID
Collision Energy Mode	Fixed
CID Collision Energy (%)	30
CID Activation Time (ms)	10
Activation Q	0.25
Multistage Activation	Off
Detector Type	Orbitrap
Orbitrap Resolution	30000
Mass Range	Normal
Scan Range Mode	Auto
Normalized AGC Target (%)	40
Maximum Injection Time Mode	Dynamic
Microscans	1
Data type	Centroid

Higher-energy collisional Dissociation (ddMS^2 OT HCD)

Isolation Mode	Quadrupole
Isolation window (m/z)	1.3
Isolation offset	Off
Activation Type	HCD
Collision Energy Mode	Stepped
HCD Collision Energy (%)	25, 30, 35
Detector Type	Orbitrap
Orbitrap Resolution	30000
Mass Range	Normal
Scan Range Mode	Auto
Normalized AGC Target (%)	40
Maximum Injection Time Mode	Dynamic
Microscans	1
Data type	Centroid

***De novo* sequencing and data refinement**

Data from Tandem Mass Spectrometry (.raw files) was processed using PEAKS Studio 10.5:

PEAKS Refinement parameters:
Enzyme: None
Merge scans: HCD and CID were merged within a 0.2 minute and 0.02 Da window
Accepted charge states: 2-8

PEAKS *De novo* parameters:
Precursor mass error < 15 ppm
Fragment Ion Mass error < 0.02 Da
Candidates per spectrum: 5
Post-translation Modifications:
C-terminal amide (-0.98 Da, fixed, [X]@C-term)
S-acetamide (+57.02 Da, fixed, [C]) **or** S-**mxy**-S-Cys-PTH (+340.07 Da, fixed, [C])

PEAKS output was processed using a Python Script (GetPep.py; Vinogradov, A. A. *et al*. Library Design-Facilitated High-Throughput Sequencing of Synthetic Peptide Libraries. *ACS Comb. Sci*. 19, 694–701 (2017)). Briefly, the raw *de novo* output ('all de novo candidates'; up to 5 candidates per spectrum) was filtered for:
1) correct length
2) correct N- and C-terminal 'fixed' residues
3) of the remaining assignments, the top-scoring assignment per spectrum was kept
4) exact duplicate assignments were discarded

Note: the 'refinement' step originally described with GetPep.py (Vinogradov *et al* 2017) was not employed

__Acetamide capping of KRpep('SH')__

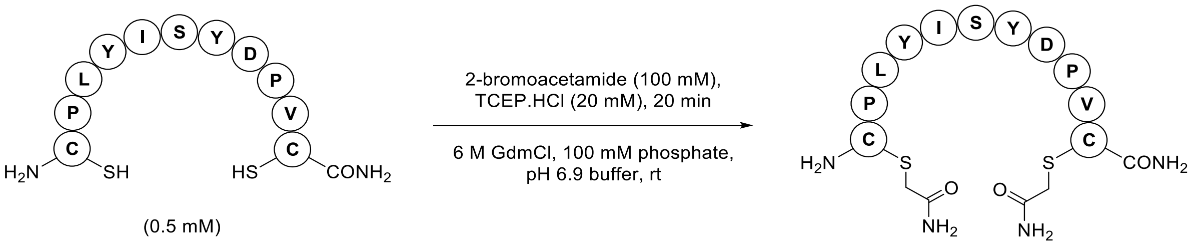

Preparation of reaction buffer

The reaction buffer was prepared by dissolving tris(2-carboxyethyl)phosphine hydrochloride (257.6 mg, 0.9 mmol) and 2-bromoacetamide (620.8 mg, 4.5 mmol) in 6 M guanidinium chloride, 100 mM phosphate, pH 6.9 buffer (22.5 mL). The resulting solution was adjusted back to pH 6.9 using aqueous NaOH after each addition. The reaction buffer was used immediately after its preparation to minimize the hydrolysis of 2-bromoacetamide.

Reaction

KRpep('SH') (5 mg, 3.93 µmol) was dissolved in 6 M guanidinium chloride, 100 mM phosphate, pH 6.9 buffer (3.93 mL). A t_0 aliquot was taken for LC-MS analysis (8 µL injection volume, 5 µg). The reaction buffer was added (3.93 mL), and the mixture was left to react for 20 min before being quenched by the addition of dithiothreitol (242.7 mg, 1.57 mmol). The resulting solution was subjected to large-scale solid phase extraction (Page S23). The eluates were aliquoted for LC-MS analysis (2 µL injection volume, 5 µg, **Figure S20**), before being flash frozen and lyophilized to afford KRpep('S-acetamide') as a white solid (4.28 mg, 3.09 µmol, 79% yield). The reaction was conducted in parallel with KRpep('SH') library.

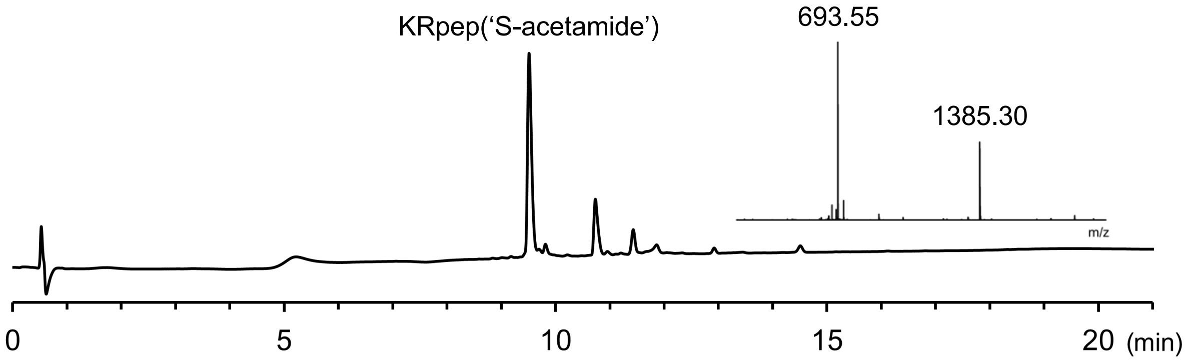

Figure S20. UV chromatogram from LC-MS analysis of the reaction product, with inlaid mass spectrum integrated across the principal UV component. Monoisotopic *m/z* calculated for $[M+H]^+$ 1385.62, found 1385.30.

Acetamide capping of KRpep('SH') library

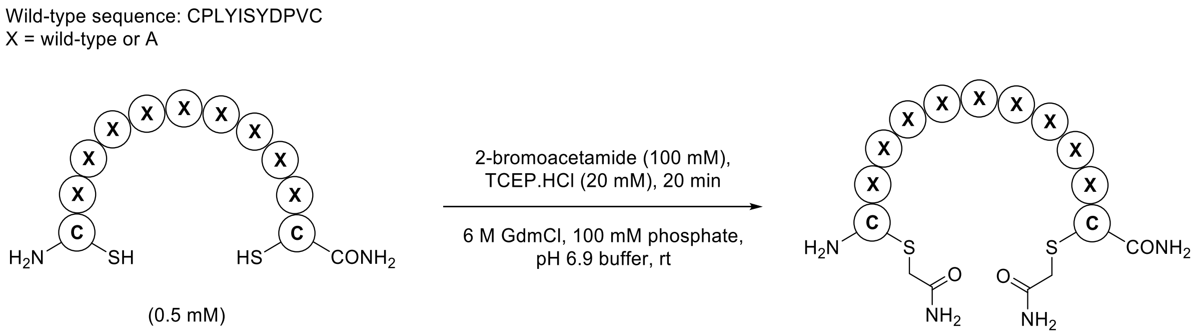

Reaction

KRpep('SH') library (5 mg, 4.68 μmol total; 9.14 nmol/peptide nominal) was dissolved in 6 M guanidinium chloride, 100 mM phosphate, pH 6.9 buffer (4.68 mL). The reaction buffer from the preceding entry (Page S28) was added (4.68 mL), and the mixture was left to react for 20 min. The reaction was quenched by the addition of dithiothreitol (289.2 mg, 1.87 mmol). The resulting solution was subjected to large-scale solid phase extraction (Page S23). The eluates were flash frozen and lyophilized to afford KRpep('S-acetamide') library as a white solid (2.79 mg, 2.36 μmol total; 4.61 nmol/peptide nominal, 50% yield). The reaction was conducted in parallel with KRpep('SH').

Preparation of stock solutions for nLC-MS analysis

The KRpep('S-acetamide') library (2.79 mg, 2.36 μmol total; 4.61 nmol/peptide nominal) was dissolved in 50/50 Solvent A/B (470 μL) to give a nominally 5 mM solution, which was then centrifuged (4,000 rpm, 2 min). The supernatant (5 μL) was diluted in MS grade water (45 μL), and the peptide concentration of the resulting solution was analyzed with a Thermo Scientific™ NanoDrop™ Eight UV-Vis spectrophotometer. Based on the measured peptide concentration, the supernatant of the nominally 5 mM solution was then serially diluted using MS Mobile Phase (2.5% MS grade acetonitrile, 97.5% MS grade water, 0.05% acetic acid) spiked with 2 fmol/peptide/μL Pierce Peptide Retention Time Calibration Mixture to yield 20, 10, and 2 fmol/peptide/μL solutions of the KRpep('S-acetamide') library. The solutions were then analyzed by nLC-MS ('standard run', Page S25).

α,α'-Dibromo-*m*-xylene cyclization of KRpep('SH')

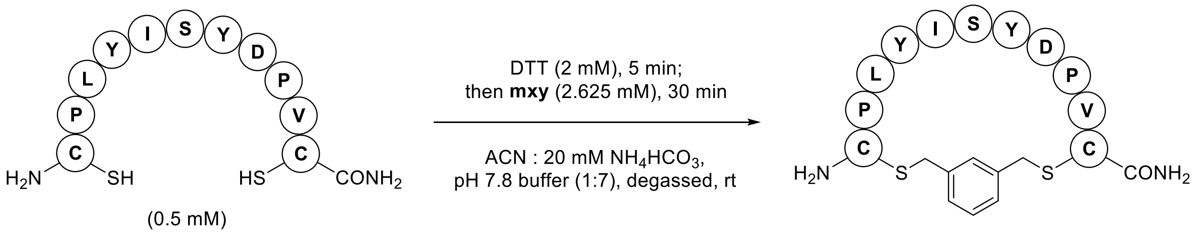

KRpep('SH') (5 mg, 3.93 µmol) was dissolved in aqueous 20 mM ammonium bicarbonate, pH 7.8 buffer (6.72 mL). 0.1 M aqueous dithiothreitol solution (157 µL) was added and left to react for 5 min. A t_0 aliquot (20 µL) was then diluted in aqueous 6 M guanidinium chloride, 100 mM phosphate, pH 6.9 buffer (53 µL) for LC-MS analysis (25 µL injection volume, 5 µg). 21 mM α,α'-dibromo-*m*-xylene in degassed acetonitrile (0.98 mL) was then added to the solution and left to react for 30 min, then washed with cold diethyl ether (3x, approx. 7.9 mL each). Excess ether was evaporated over a stream of inert gas, and the resulting solution was diluted 4-fold in aqueous 6 M guanidinium chloride, 200 mM phosphate, pH 3 buffer (23.6 mL). The solution was filtered through a 0.45 µm nylon filter and aliquoted for LC-MS analysis (24 µL injection volume, 5 µg). The filtered solution was subjected to large-scale solid phase extraction (Page S23), and the eluates were aliquoted for LC-MS analysis (2 µL injection volume, 5 µg, **Figure S21**). The eluates were flash frozen and lyophilized to afford KRpep('S-**mxy**-S') as a white solid (3.60 mg, 2.62 µmol, 67% yield). The reaction was conducted in parallel with KRpep('SH') library.

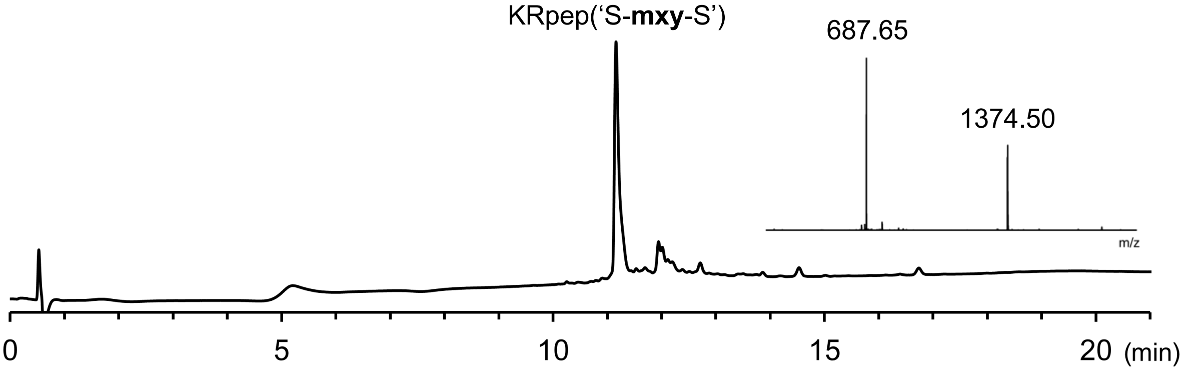

Figure S21. UV chromatogram from LC-MS analysis of the reaction product, with inlaid mass spectrum integrated across the principal UV component. Monoisotopic *m/z* calculated for $[M+H]^+$ 1373.63, found 1374.50.

α,α’-Dibromo-*m*-xylene cyclization of KRpep(‘SH’) library

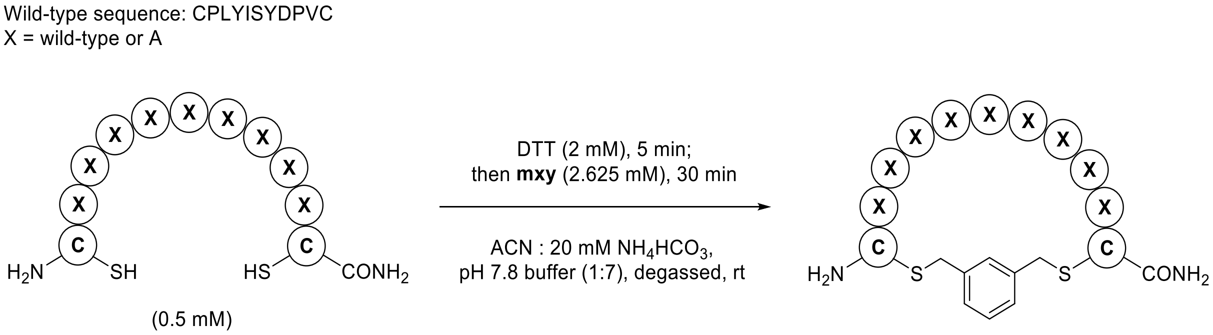

KRpep(‘SH’) library (12 mg, 11.24 μmol total; 22 nmol/peptide nominal) was dissolved in aqueous 20 mM ammonium bicarbonate, pH 7.8 buffer (19.23 mL). 0.1 M aqueous dithiothreitol solution (0.45 mL) was added and left to react for 5 min. 21 mM α,α’-dibromo-*m*-xylene in degassed acetonitrile (2.81 mL) was then added to the solution and left to react for 30 min, then washed with cold diethyl ether (3x, approx. 22.5 mL each). Excess ether was evaporated over a stream of inert gas, and the resulting solution was diluted 4-fold in aqueous 6 M guanidinium chloride, 200 mM phosphate, pH 3 buffer (67.5 mL). The solution was filtered through a 0.45 μm nylon filter. The filtered solution was subjected to large-scale solid phase extraction (Page S23). The eluates were flash frozen and lyophilized to afford KRpep(‘S-**mxy**-S’) library as a white solid (6.08 mg, 5.20 μmol total; 10.2 nmol/peptide nominal, 46% yield). The reaction was conducted in parallel with KRpep(‘SH’).

PITC installation on KRpep('S-mxy-S')

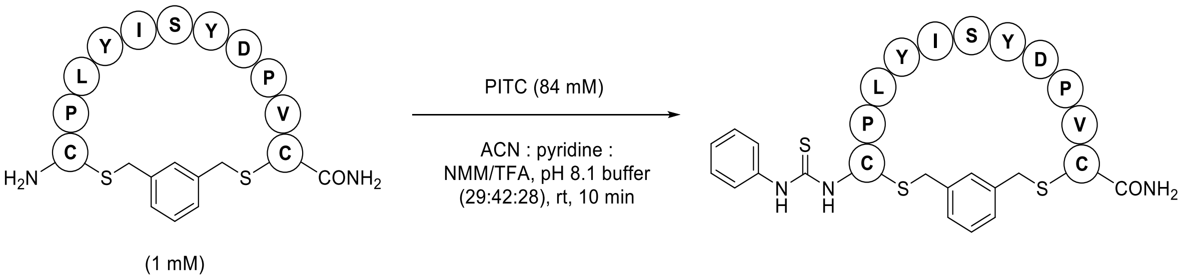

Preparation of pH 8.1 NMM/TFA buffer

N-methylmorpholine (1.4 mL) and trifluoroacetic acid (0.19 mL) were diluted in deionized water (8.4 mL) and the pH was adjusted to 8.1.

Reaction

Pyridine (1.26 mL) was combined with the pH 8.1 NMM/TFA buffer (0.84 mL). KRpep('S-**mxy**-S') (3 mg, 2.18 µmol) was dissolved in the pyridine-buffer mixture (1.53 mL), and a t_0 aliquot was diluted 10-fold in Solvent A for LC-MS analysis (25 µL injection volume, 5 µg). Phenyl isothiocyanate (30 µL) was diluted in acetonitrile (0.87 mL) to give a 3.33% (v/v) PITC solution. The PITC solution (0.66 mL) was then added to the peptide solution and left to react for 10 min. The reaction mixture was diluted 10-fold by addition of Solvent A (19.66 mL) and centrifuged until the supernatant was clear (4,000 rpm, 30 min). The supernatant was carefully decanted with a syringe and aliquoted for LC-MS analysis (36 µL injection volume, 5 µg). The supernatant was washed with diethyl ether (3x, approx. 21.8 mL each) and excess ether was evaporated over a stream of inert gas. The resulting solution was aliquoted for LC-MS analysis (36 µL injection volume, 5 µg) and subjected to large-scale solid phase extraction (Page S23). The eluates were aliquoted for LC-MS analysis (3.3 µL injection volume, 5 µg), which revealed that the desired product had eluted in the "Loading" fraction. The fraction was then divided into equal portions (3 x 7 mL), and a t_0 aliquot was taken for LC-MS analysis (35 µL injection volume, 5 µg). The first portion was left unchanged; the second portion was diluted 2-fold in Solvent A (7 mL) and aliquoted for LC-MS analysis (40 µL injection volume, 2.8 µg); a stream of N_2 was passed over the third portion to evaporate any residual ether, and an aliquot was taken for LC-MS analysis (25 µL injection volume, 5 µg, **Figure S22**). All 3 portions were subjected to solid phase extraction as earlier, and the eluates were analyzed by LC-MS (10 µL injection volume, 5 µg), which showed that the peptide had eluted as expected. As the contents of the eluates of all 3 portions were identical, they were combined after flash freezing and lyophilization to afford PITC-KRpep('S-**mxy**-S') as a white solid (1.38 mg, 0.91 µmol, 42% yield).

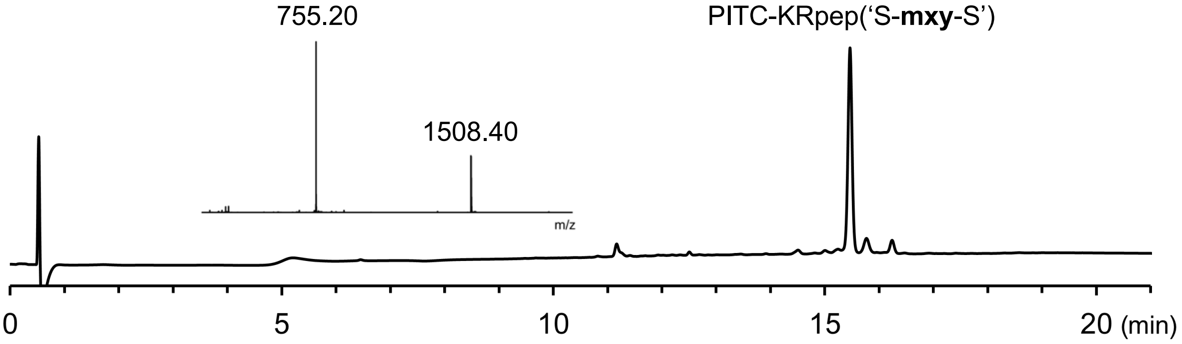

Figure S22. UV chromatogram from LC-MS analysis of the reaction product, with inlaid mass spectrum integrated across the principal UV component. Monoisotopic *m/z* calculated for $[M+H]^+$ 1508.64, found 1508.40.

PITC installation on KRpep('S-mxy-S') library

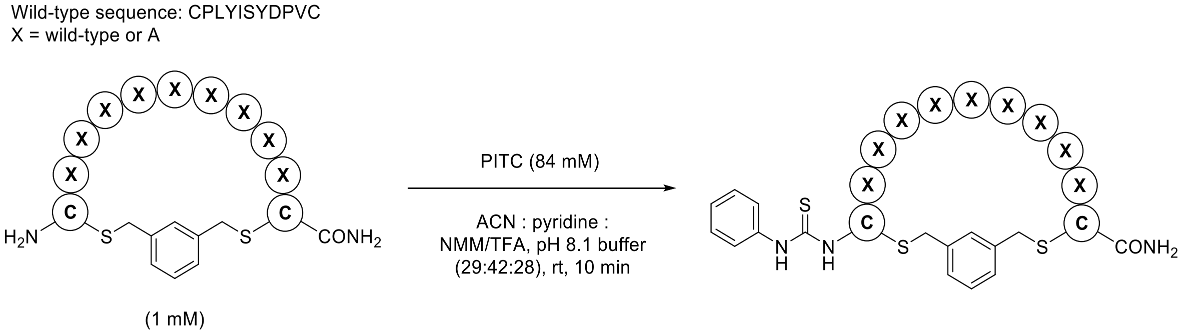

Reaction

Pyridine (2.1 mL) was combined with the pH 8.1 NMM/TFA buffer from the preceding entry (Page S32) (1.4 mL). KRpep('S-**mxy**-S') library (3 mg, 2.56 µmol total; 5 nmol/peptide nominal) was dissolved in the pyridine-buffer mixture (1.79 mL), and a t_0 aliquot was diluted 10-fold in Solvent A for LC-MS analysis (30 µL injection volume, 5 µg). Phenyl isothiocyanate (50 µL) was diluted in acetonitrile (1.45 mL) to give a 3.33% (v/v) PITC solution. The PITC solution (0.77 mL) was then added to the peptide solution and left to react for 10 min. The reaction mixture was diluted 10-fold by addition of Solvent A (23.07 mL) and centrifuged until the supernatant was clear (4,000 rpm, 30 min). The supernatant was carefully decanted with a syringe and aliquoted for LC-MS analysis (40 µL injection volume, 5 µg). The supernatant was washed with diethyl ether (3x, approx. 25.6 mL each) and excess ether was evaporated over a stream of inert gas. The solution was aliquoted for LC-MS analysis (40 µL injection volume, 5 µg), and subjected to large-scale solid phase extraction (Page S23). The eluates were aliquoted for LC-MS analysis (3.3 µL injection volume, 5 µg, **Figure S23**), and flash frozen and lyophilized to afford PITC-KRpep('S-**mxy**-S') library as a white solid (1.49 mg, 1.14 µmol total; 2.23 nmol/peptide nominal, 45% yield). The reaction was conducted in parallel with KRpep('S-**pps**-S') library.

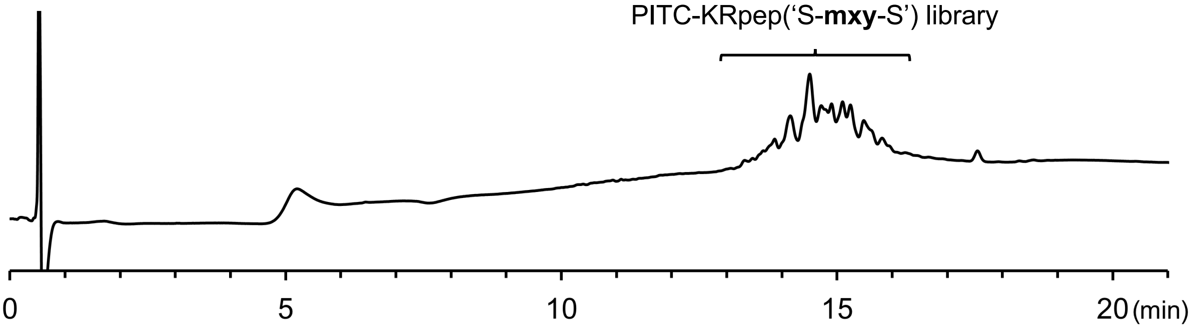

Figure S23. UV chromatogram of the reaction product.

Linearization of PITC-KRpep('S-mxy-S')

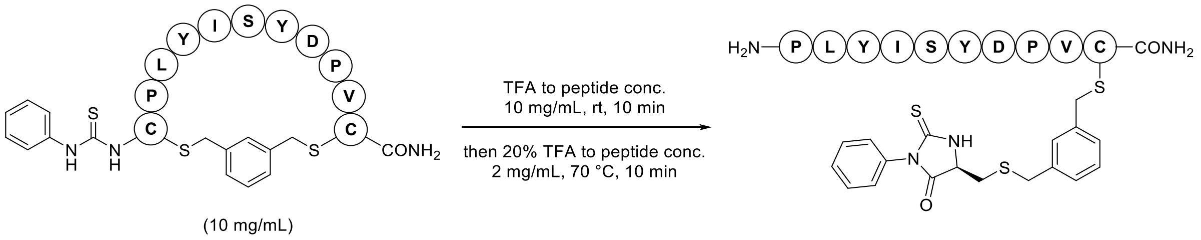

PITC-KRpep('S-**mxy**-S') (0.1 mg, 66 nmol) was dissolved in trifluoroacetic acid (10 µL) and left to react for 10 min. The solution was diluted 5-fold with deionized water (40 µL) and heated to 70 °C for 10 min. The solution was then diluted with aqueous 6 M guanidinium chloride, 100 mM phosphate, pH 7 buffer (150 µL), and subjected to small-scale solid phase extraction (Page S23) with 4 x 0.1 mL elution fractions. Aliquots of the eluates were taken before LC-MS analysis (25 µL injection volume, 5 µg, **Figure S24**). The product was not isolated. The reaction was conducted in parallel with PITC-KRpep('S-**pps**-S').

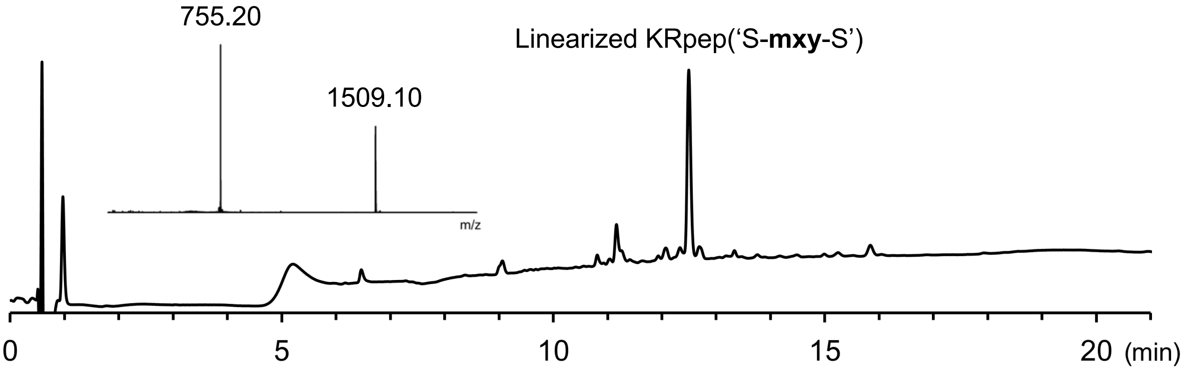

Figure S24. UV chromatogram from LC-MS analysis of the reaction product, with inlaid mass spectrum integrated across the principal UV component. Monoisotopic *m/z* calculated for $[M+H]^+$ 1508.64, found 1509.10.

<u>**Linearization of PITC-KRpep('S-mxy-S') library**</u>

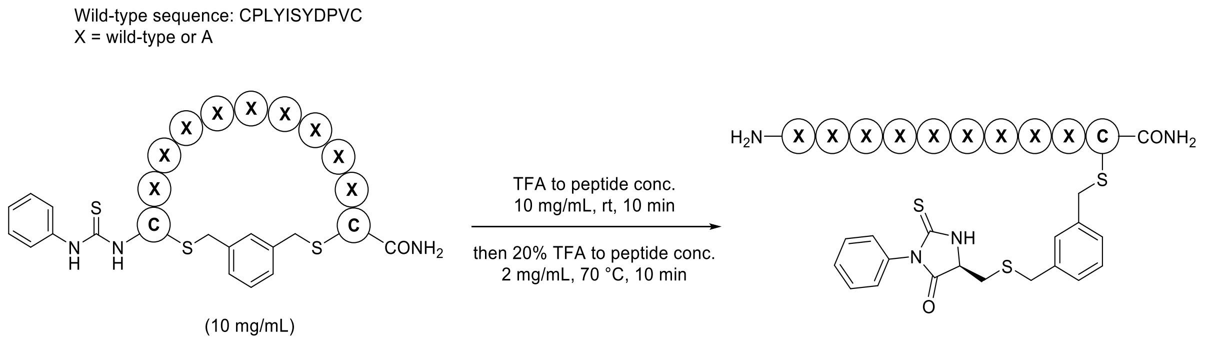

PITC-KRpep('S-**mxy**-S') library (0.75 mg, 0.57 µmol total; 1.11 nmol/peptide nominal) was dissolved in trifluoroacetic acid (75 µL) and left to react for 10 min. The solution was diluted 5-fold with deionized water (0.3 mL) and heated to 70 °C for 10 min. The solution was then diluted with aqueous 6 M guanidinium chloride, 100 mM phosphate, pH 7 buffer (1.13 mL), and subjected to small-scale solid phase extraction (Page S23). Aliquots of the eluates were taken before LC-MS analysis (25 µL injection volume, 5 µg, **Figure S25**). Eluates 1 & 2 were flash frozen and lyophilized to afford linearized KRpep('S-**mxy**-S') library as a white solid (0.69 mg, 0.53 µmol total; 1.03 nmol/peptide nominal, 92% yield). The reaction was conducted in parallel with PITC-KRpep('S-**pps**-S') and PITC-KRpep('S-**pps**-S') library.

Preparation of stock solutions for nLC-MS analysis

The linearized KRpep('S-**mxy**-S') library (0.69 mg, 0.53 µmol total; 1.03 nmol/peptide nominal) was dissolved in 50/50 Solvent A/B (106 µL) to give a nominally 5 mM solution, which was then centrifuged (4,000 rpm, 2 min). The supernatant (2 µL) was diluted in MS grade water (18 µL), and the peptide concentration of the resulting solution was analyzed with a Thermo Scientific™ NanoDrop™ Eight UV-Vis spectrophotometer, which gave calculated results exceeding the nominal peptide concentration. Hence, based on the nominal peptide concentration, the supernatant of the nominally 5 mM solution was then serially diluted using MS Mobile Phase (2.5% MS grade acetonitrile, 97.5% MS grade water, 0.05% acetic acid) spiked with 2 fmol/peptide/µL Pierce Peptide Retention Time Calibration Mixture to yield 20, 10, and 2 fmol/peptide/µL solutions of the linearized KRpep('S-**mxy**-S') library. The solutions were then analyzed by nLC-MS ('standard run', Page S25).

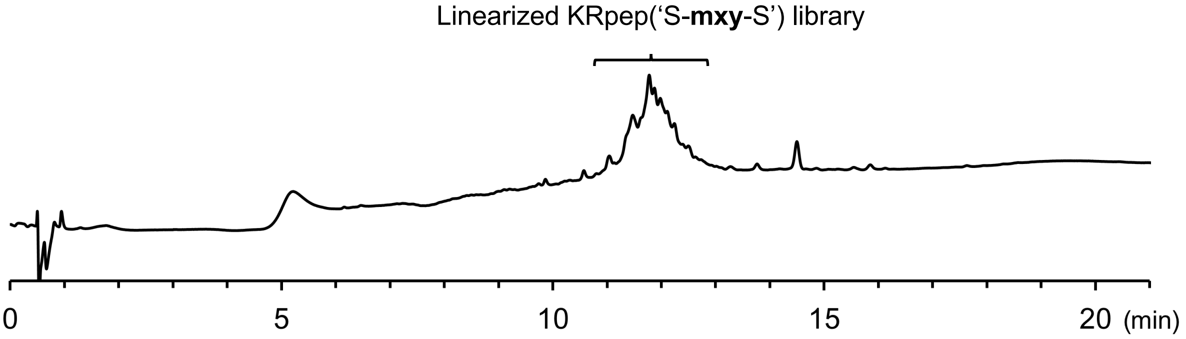

Figure S25. UV chromatogram of the reaction product.

Pentafluorophenyl sulfide cyclization of KRpep('SH')

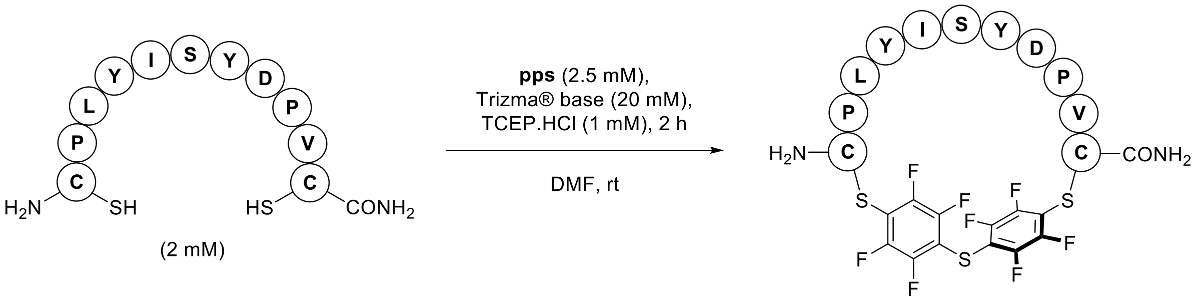

KRpep('SH') (5 mg, 3.93 µmol) was dissolved in dimethyl formamide (0.84 mL). A t_0 aliquot was diluted 10-fold in 50:50 Solvent A:B for LC-MS analysis (18 µL injection volume, 5 µg). 50 mM Trizma® base in dimethyl formamide (0.79 mL), 20 mM tris(2-carboxyethyl)phosphine hydrochloride in dimethyl formamide (98 µL), and 20 mM pentafluorophenyl sulfide in dimethyl formamide (0.25 mL) were added to the peptides, and the solutions were left to react for 2 h. The solution was then diluted 20-fold with Solvent A (37 mL), aliquoted for LC-MS analysis (40 µL injection volume, 5 µg), and subjected to large-scale solid phase extraction (Page S23). The eluates were aliquoted for LC-MS analysis (2 µL injection volume, 5 µg, **Figure S26**). The eluates were flash frozen and lyophilized to afford KRpep('S-**pps**-S') as a white solid (5.12 mg, 3.20 µmol, 81% yield). The reaction was conducted in parallel with KRpep('SH') library.

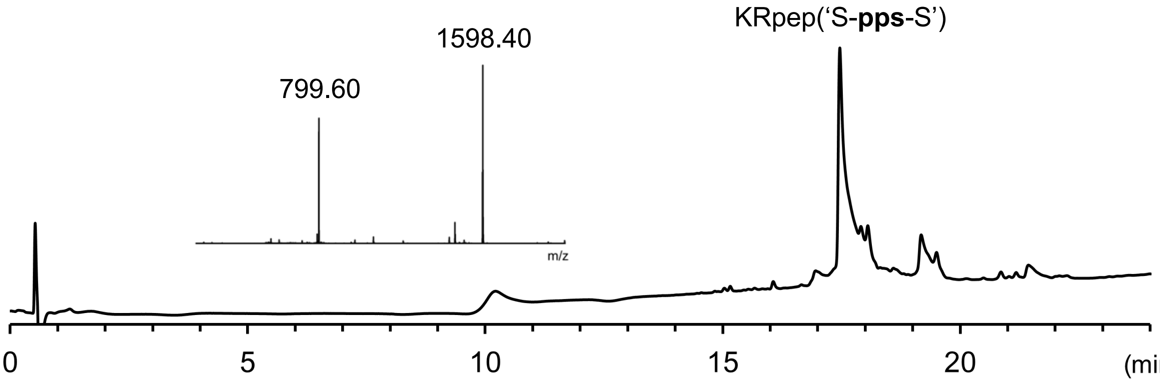

Figure S26. UV chromatogram from LC-MS analysis of the reaction product, with inlaid mass spectrum integrated across the principal UV component. Monoisotopic *m/z* calculated for $[M+H]^+$ 1597.52, found 1598.40.

Pentafluorophenyl sulfide cyclization of KRpep('SH') library

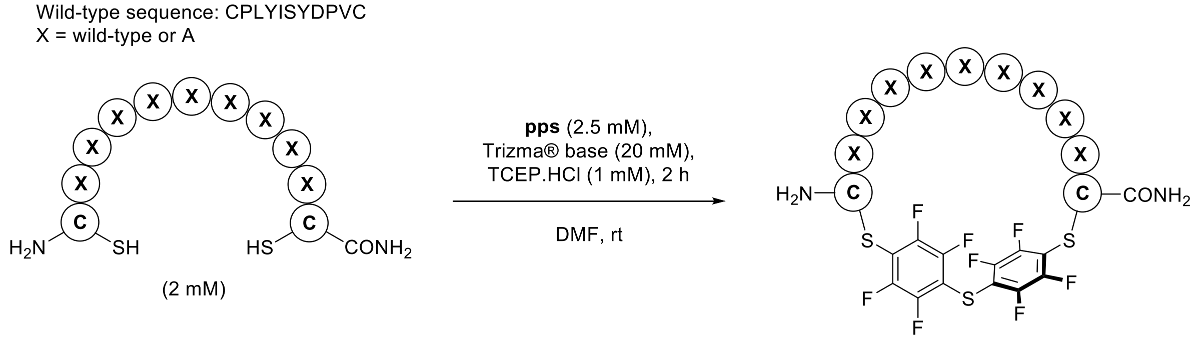

KRpep('SH') library (12 mg, 11.24 µmol total; 22 nmol/peptide nominal) was dissolved in dimethyl formamide (2.39 mL). 50 mM Trizma® base in dimethyl formamide (2.25 mL), 20 mM tris(2-carboxyethyl)phosphine hydrochloride in dimethyl formamide (0.28 mL), and 20 mM pentafluorophenyl sulfide in dimethyl formamide (0.7 mL) were added to the peptides, and the solutions were left to react for 2 h. The solution was then diluted 20-fold with Solvent A (107 mL) and subjected to large-scale solid phase extraction (Page S23). The eluates were flash frozen and lyophilized to afford KRpep('S-**pps**-S') library as a white solid (9.43 mg, 6.77 µmol total; 13.2 nmol/peptide nominal, 60% yield). The reaction was conducted in parallel with KRpep('SH').

PITC installation on KRpep('S-pps-S')

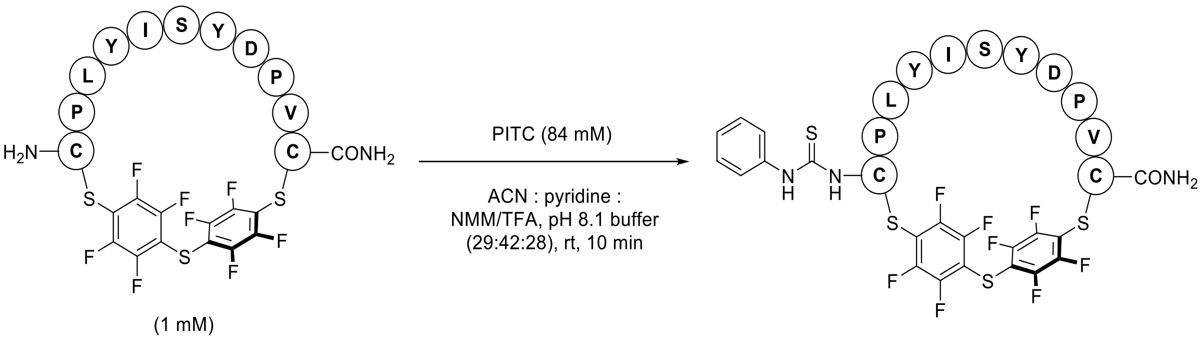

Pyridine (0.63 mL) was combined with the pH 8.1 NMM/TFA buffer from Page S32 (0.42 mL). KRpep('S-**pps**-S') (1 mg, 0.63 μmol) was dissolved in the pyridine-buffer mixture (0.44 mL), and a t_0 aliquot was diluted 10-fold in Solvent A for LC-MS analysis (22 μL injection volume, 5 μg). Phenyl isothiocyanate (15 μL) was diluted in acetonitrile (0.44 mL) to give a 3.33% (v/v) PITC solution. The PITC solution (188 μL) was then added to the peptide solution and left to react for 10 min. The reaction mixture was diluted 10-fold by addition of Solvent A (5.64 mL) and centrifuged until the supernatant was clear (4,000 rpm, 30 min). The supernatant was carefully decanted with a syringe and aliquoted for LC-MS analysis (30 μL injection volume, 5 μg). The supernatant was washed with diethyl ether (3x, approx. 6.3 mL each) and excess ether was evaporated over a stream of inert gas. An aliquot was taken for LC-MS analysis (30 μL injection volume, 5 μg). The solution was subjected to large-scale solid phase extraction (Page S23), and the eluates were aliquoted for LC-MS analysis (10 μL injection volume, 5 μg, **Figure S27**). The eluates were flash frozen and lyophilized to afford PITC-KRpep('S-**pps**-S') as a white solid (0.38 mg, 0.22 μmol, 35% yield).

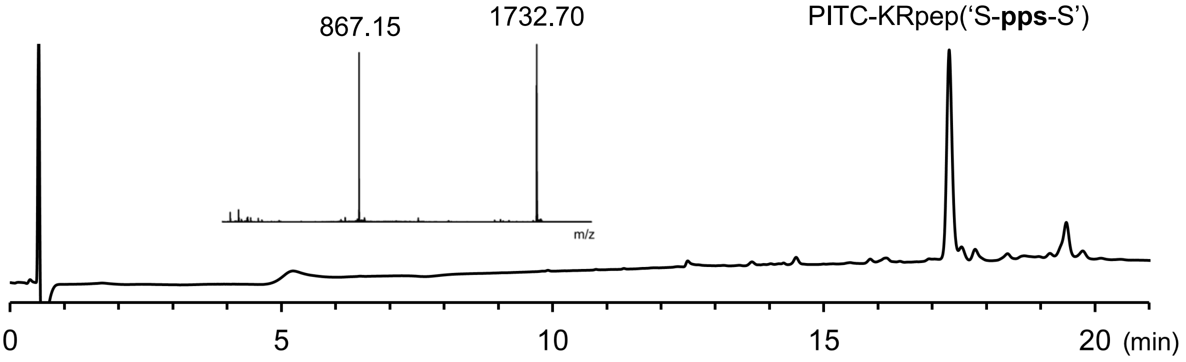

Figure S27. UV chromatogram from LC-MS analysis of the reaction product, with inlaid mass spectrum integrated across the principal UV component. Monoisotopic *m/z* calculated for $[M+H]^+$ 1732.54, found 1732.70.

PITC installation on KRpep('S-pps-S') library

Wild-type sequence: CPLYISYDPVC
X = wild-type or A

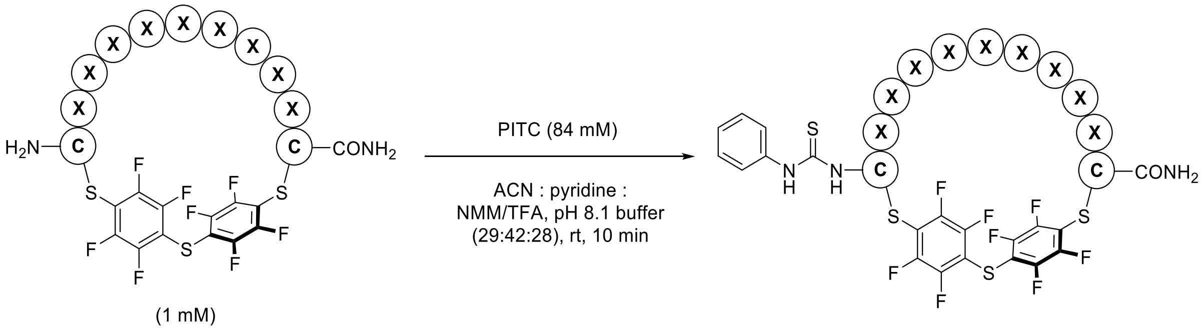

Pyridine (2.1 mL) was combined with the pH 8.1 NMM/TFA buffer from Page S32 (1.4 mL). KRpep('S-**pps**-S') library (3 mg, 2.15 µmol total; 4.20 nmol/peptide nominal) was dissolved in the pyridine-buffer mixture (1.51 mL), and a t_0 aliquot was diluted 10-fold in Solvent A for LC-MS analysis (25 µL injection volume, 5 µg). Phenyl isothiocyanate (50 µL) was diluted in acetonitrile (1.45 mL) to give a 3.33% (v/v) PITC solution. The PITC solution (0.65 mL) was then added to the peptide solution and left to react for 10 min. The reaction mixture was diluted 10-fold by addition of Solvent A (19.38 mL) and centrifuged until the supernatant was clear (4,000 rpm, 1 h). The supernatant was carefully decanted with a syringe and aliquoted for LC-MS analysis (35 µL injection volume, 5 µg). The supernatant was washed with diethyl ether (3x, approx. 21.5 mL each) and excess ether was evaporated over a stream of inert gas. An aliquot was taken for LC-MS analysis (35 µL injection volume, 5 µg). The solution was subjected to large-scale solid phase extraction (Page S23), and the eluates were aliquoted for LC-MS analysis (3.3 µL injection volume, 5 µg, **Figure S28**). The eluates were flash frozen and lyophilized to afford PITC-KRpep('S-**pps**-S') library as a white solid (1.40 mg, 0.92 µmol total; 1.80 nmol/peptide nominal, 43% yield). The reaction was conducted in parallel with KRpep('S-**mxy**-S') library.

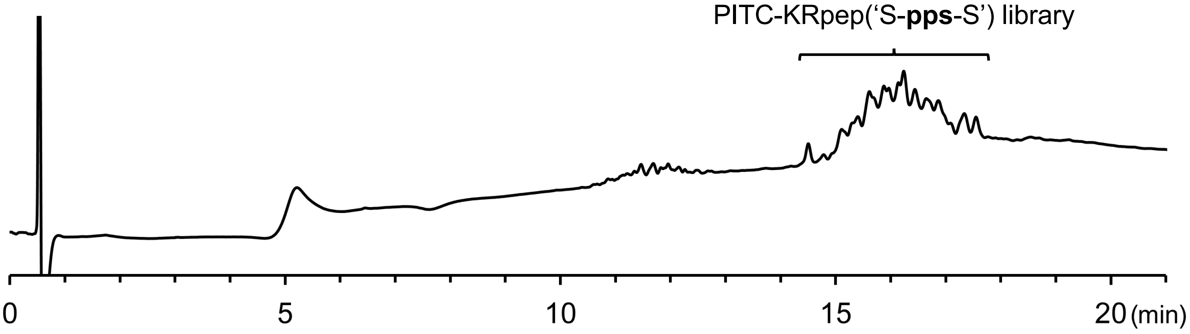

Figure S28. UV chromatogram of the reaction product.

Attempted linearization of PITC-KRpep(‘S-pps-S’)

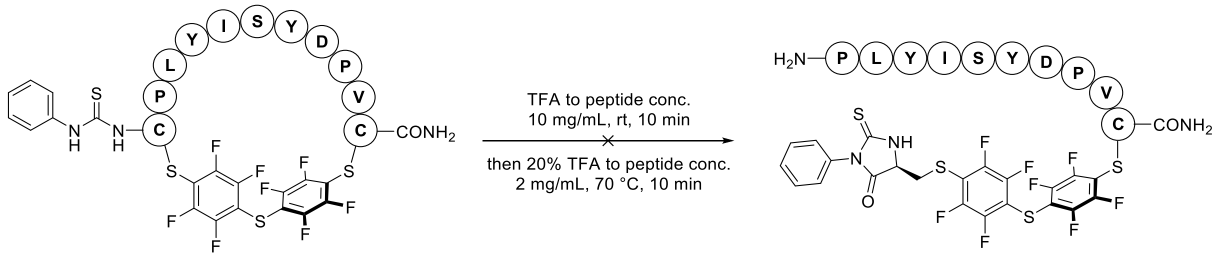

PITC-KRpep(‘S-**pps**-S’) (0.38 mg, 0.22 μmol) was dissolved in trifluoroacetic acid (38 μL) and left to react for 10 min. The solution was diluted 5-fold with deionized water (152 μL) and heated to 70 °C for 10 min. The solution was then diluted with aqueous 6 M guanidinium chloride, 100 mM phosphate, pH 7 buffer (0.57 mL), and subjected to small-scale solid phase extraction (Page S23). Aliquots of the eluates were diluted 10-fold in Solvent A for LC-MS analysis (26 μL injection volume, 5 μg, **Figure S29**). The reaction was conducted in parallel with PITC-KRpep(‘S-**mxy**-S’) library and PITC-KRpep(‘S-**pps**-S’) library.

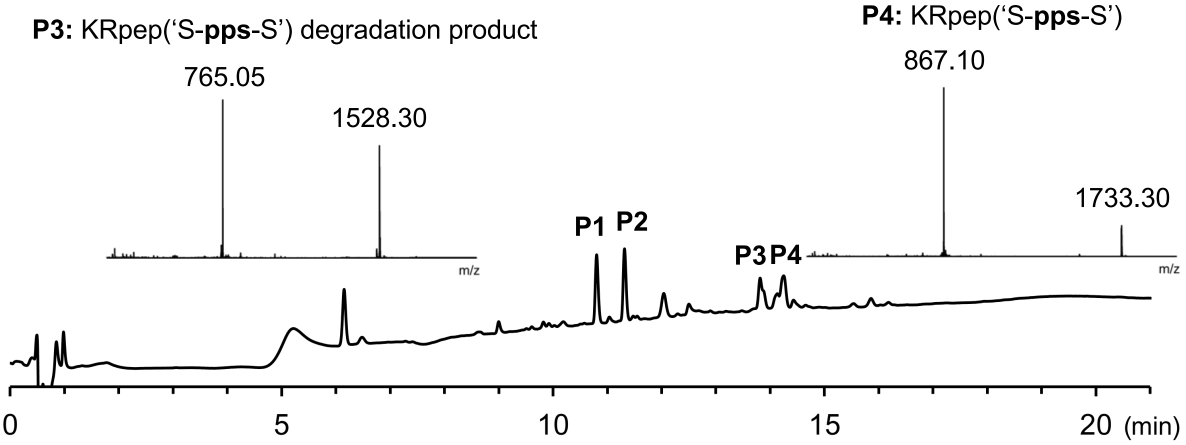

Figure S29. UV chromatogram from LC-MS analysis of the reaction product, with inlaid mass spectrum integrated across the peaks P3 and P4 respectively. *m/z* calculated for $[M+H]^+$ 1732.54, found 1733.30. Peaks P1 and P2 could not be identified.

Attempted linearization of PITC-KRpep(‘S-pps-S’) library

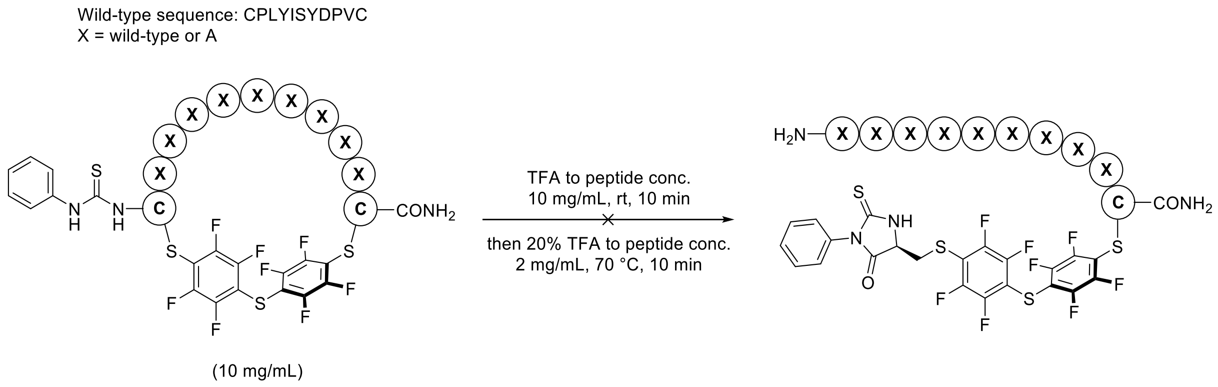

PITC-KRpep(‘S-**pps**-S’) library (0.70 mg, 0.46 μmol total; 0.90 nmol/peptide nominal) was dissolved in trifluoroacetic acid (70 μL) and left to react for 10 min. The solution was diluted 5-fold with deionized water (0.28 mL) and heated to 70 °C for 10 min. The solution was then diluted with aqueous 6 M guanidinium chloride, 100 mM phosphate, pH 7 buffer (1.05 mL), and subjected to small-scale solid phase extraction (Page S23). Aliquots of the eluates were diluted 10-fold in Solvent A for LC-MS analysis (14 μL injection volume, 5 μg, **Figure S30**). The reaction was conducted in parallel with PITC-KRpep(‘S-**mxy**-S’) library and PITC-KRpep(‘S-**pps**-S’).

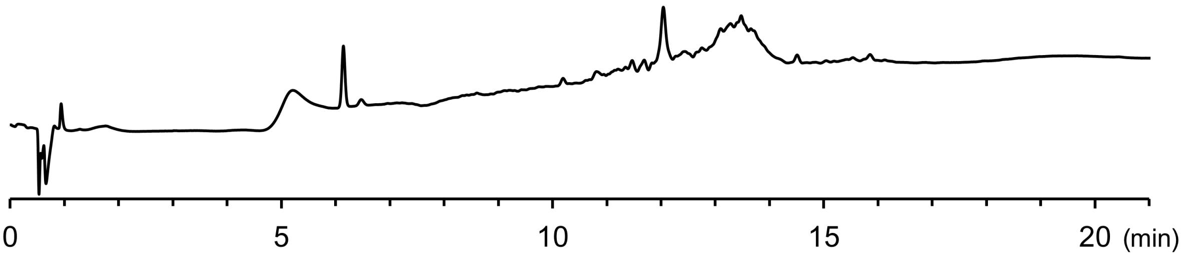

Figure S30. UV chromatogram of the reaction product.

Attempted diiodomethane cyclization of KRpep('SH') (original conditions)

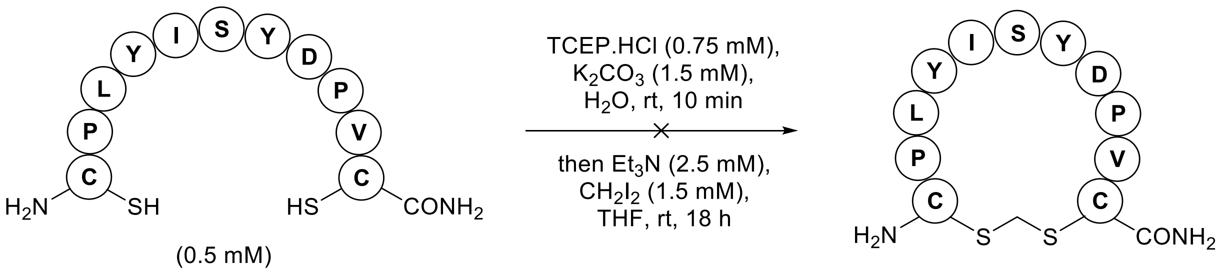

KRpep('SH') (0.1 mg, 78.6 nmol) was dissolved in 5 mM aqueous TCEP.HCl (24 µL), 5 mM aqueous potassium carbonate (47 µL), and deionized water (74 µL). A t_0 aliquot was taken, diluted 5-fold in aqueous 6 M guanidinium chloride, 100 mM phosphate, pH 2 buffer, and analyzed by LC-MS (36 µL injection volume, 5 µg). 50 mM triethylamine in THF (7.9 µL) and 50 mM diiodomethane in THF (4.7 µL) were added to the peptide solutions. At t = 30 min and t = 18 h, aliquots were taken, diluted as above, and analyzed by LC-MS (39 µL injection volume, 5 µg, **Figure S31**). The product was not isolated. The reaction was conducted in parallel with Zpep('SH').

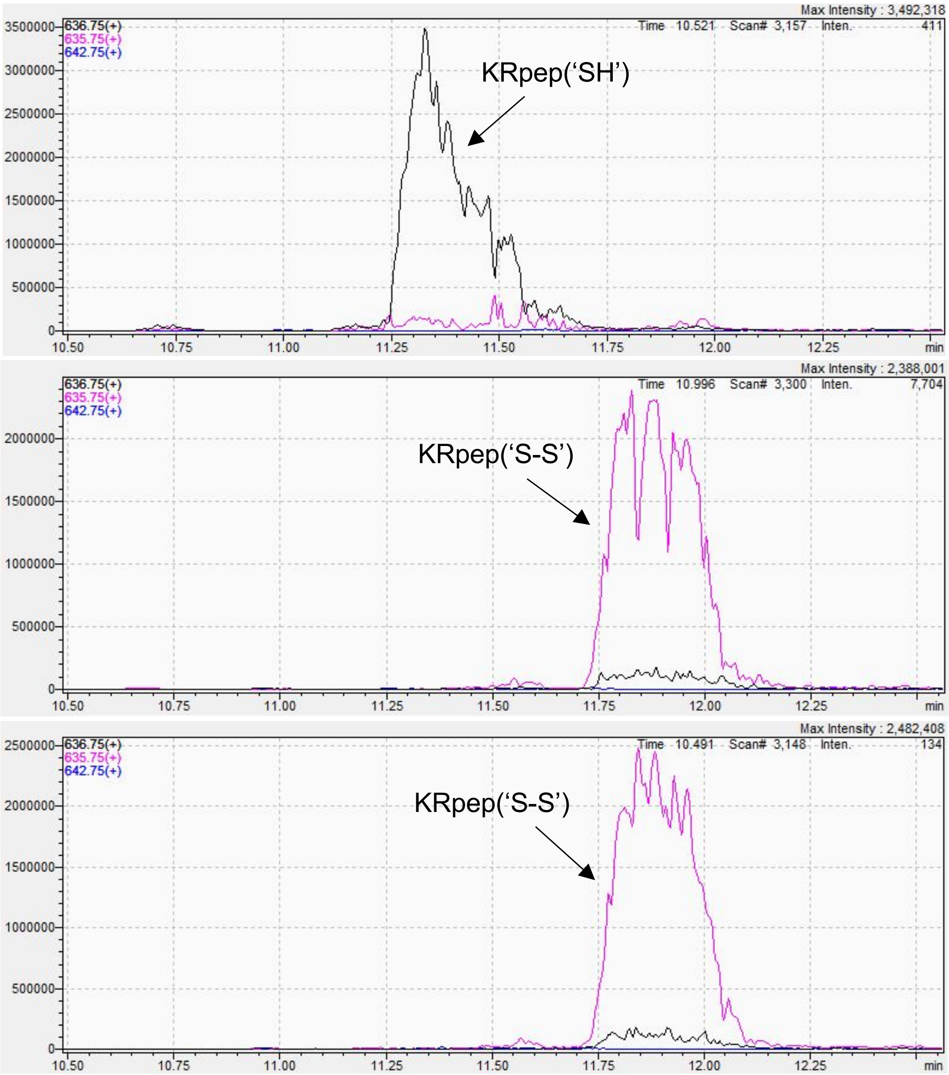

Figure S31. Extracted ion chromatograms of the reaction mixture at t = 0 (top), at t = 30 min (middle), and t = 18 h (bottom). Traces corresponding to KRpep('SH'), KRpep('S-S'), and KRpep('S-CH_2-S') are in black, magenta, and blue respectively.

Attempted diiodomethane cyclization of KRpep('SH') (adapted mxy cyclisation conditions)

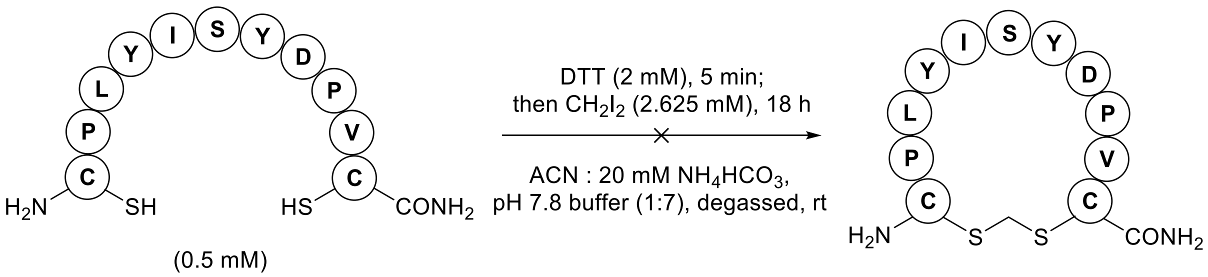

Preparation of buffer

Ammonium bicarbonate (15.81 mg, 0.2 mmol) was dissolved in degassed deionized water (10 mL) to give an aqueous 20 mM ammonium bicarbonate pH 7.8 buffer.

Preparation of dithiothreitol solution

Dithiothreitol (7.72 mg, 0.05 mmol) was dissolved in the ammonium bicarbonate buffer (0.5 mL) to give a 0.1 M dithiothreitol solution.

Preparation of diiodomethane solution in acetonitrile

Diiodomethane (5.80 µL, 0.072 mmol) was dissolved in degassed acetonitrile (0.5 mL) to give a 144 mM diiodomethane solution.

Reaction

KRpep('SH') (0.1 mg, 78.6 nmol) was dissolved in the ammonium bicarbonate buffer (135 µL) and 0.1 M dithiothreitol solution (3.2 µL). t_0 aliquots were diluted 5-fold in aqueous 6 M guanidinium chloride, 100 mM phosphate, pH 2 buffer, and analyzed by LC-MS (35 µL injection volume, 5 µg). Degassed acetonitrile (16.8 µL), followed by 144 mM diiodomethane solution (2.9 µL) were then added to the peptide solution. At t = 30 min and t = 18 h, aliquots were taken, diluted as above, and analyzed by LC-MS (40 µL injection volume, 5 µg, **Figure S32**). The product was not isolated. The reaction was conducted in parallel with Zpep('SH').

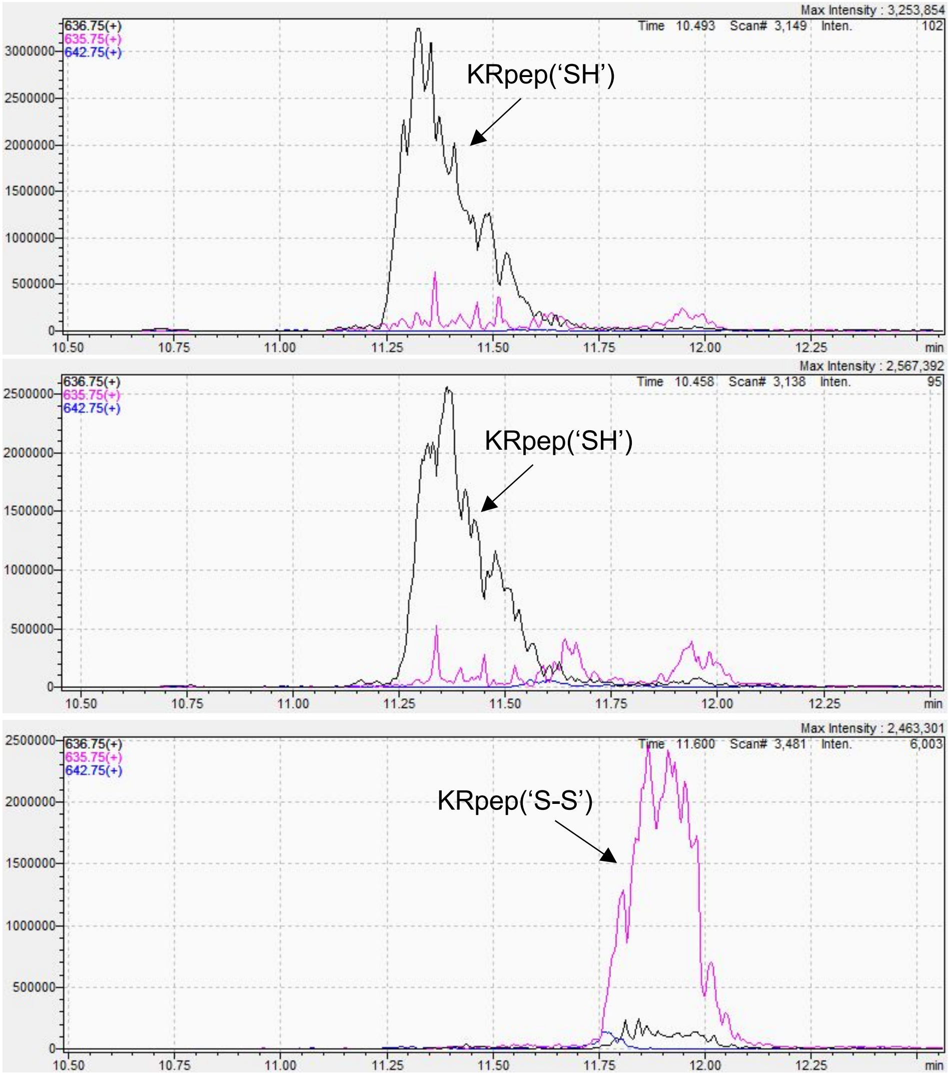

Figure S32. Extracted ion chromatograms of the reaction mixture at t = 0 (top), at t = 30 min (middle), and t = 18 h (bottom). Traces corresponding to KRpep('SH'), KRpep('S-S'), and KRpep('S-CH_2-S') are in black, magenta, and blue respectively.

Attempted diiodomethane cyclization of KRpep('SH') (A*STAR conditions)

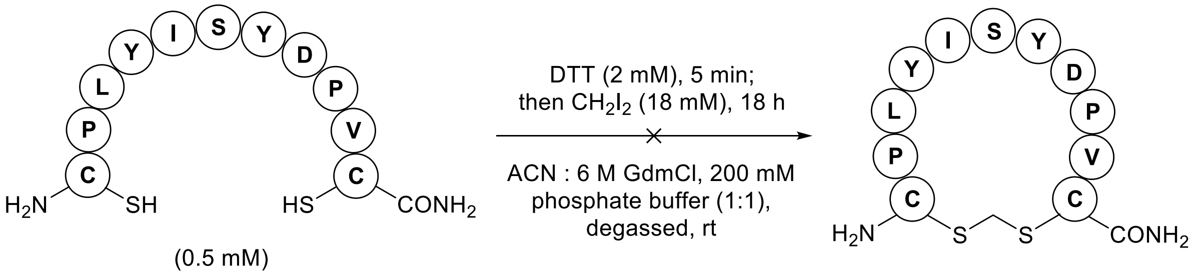

Dithiothreitol (7.72 mg, 0.05 mmol) was dissolved in 6 M guanidinium chloride, 200 mM phosphate, pH 9 buffer (0.5 mL) to give a 100 mM dithiothreitol solution. KRpep('SH') (0.1 mg, 78.6 nmol) was dissolved in the 100 mM dithiothreitol solution (3.2 µL) and 6 M guanidinium chloride, 200 mM phosphate, pH 9 buffer (76 µL) and the mixture was left to react for 5 min. A t_0 aliquot was diluted 5-fold in 6 M guanidinium chloride, 100 mM phosphate, pH 2 buffer for LC-MS analysis (20 µL, 5 µg). 36 mM diiodomethane in degassed acetonitrile (79 µL) was then added to the mixture. At t = 30 min and t = 18 h, aliquots were taken, diluted as above, and analyzed by LC-MS (39 µL injection volume, 5 µg, **Figure S33**). The product was not isolated. The reaction was conducted in parallel with Zpep('SH'), Ac-KRpep('SH'), and Ac-Zpep('SH').

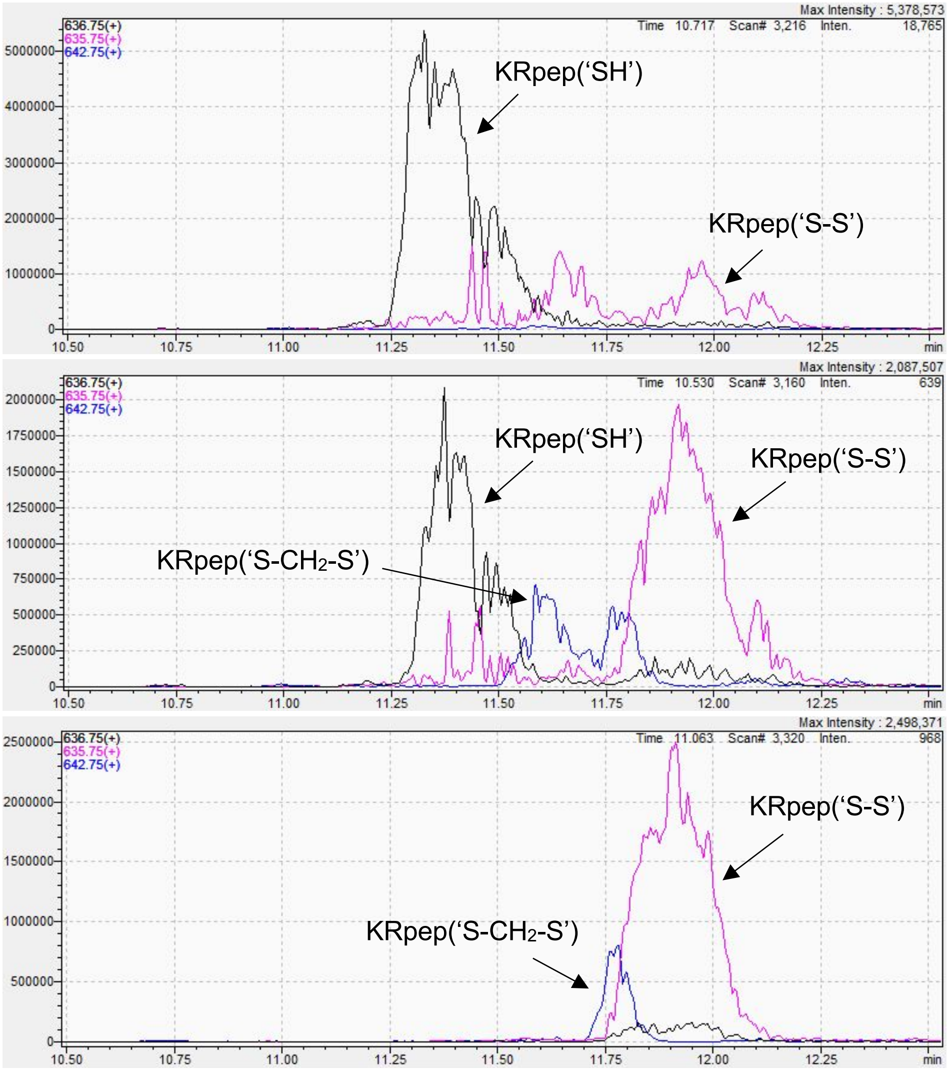

Figure S33. Extracted ion chromatograms of the reaction mixture at t = 0 (top), at t = 30 min (middle), and t = 18 h (bottom). Traces corresponding to KRpep('SH'), KRpep('S-S'), and KRpep('S-CH_2-S') are in black, magenta, and blue respectively.

Acetamide capping of Zpep('SH')

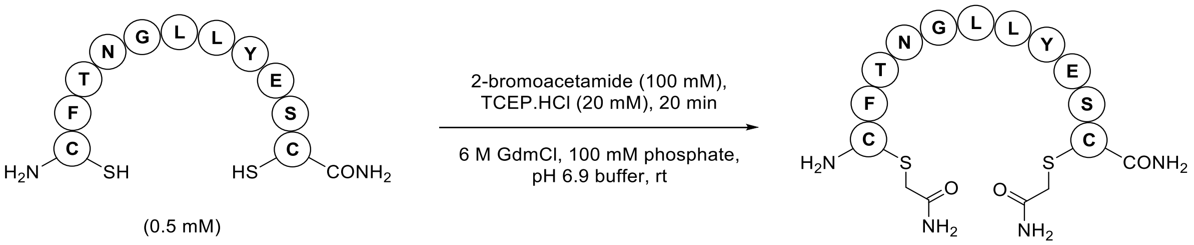

Preparation of reaction buffer

The reaction buffer was prepared by dissolving tris(2-carboxyethyl)phosphine hydrochloride (103.19 mg, 0.36 mmol) and 2-bromoacetamide (248.33 mg, 1.8 mmol) in 6 M guanidinium chloride, 100 mM phosphate, pH 6.9 buffer (9 mL). The resulting solution was adjusted back to pH 6.9 using aqueous NaOH.

Reaction

Zpep('SH') (5 mg, 4.00 µmol) was dissolved in 6 M guanidinium chloride, 100 mM phosphate, pH 6.9 buffer (4 mL). A t_0 aliquot was taken for LC-MS analysis (4 µL injection volume, 5 µg). The reaction buffer (4 mL) was added and left to react for 20 min. The reaction was quenched by the addition of dithiothreitol (247.2 mg, 1.60 mmol). The solution was aliquoted for LC-MS analysis (8 µL injection volume, 5 µg) and subjected to large-scale solid phase extraction (Page SS23). Aliquots of the eluates were diluted 10-fold in Solvent A and analyzed by LC-MS (20 µL injection volume, 5 µg, **Figure S34**). The eluates were flash frozen and lyophilized to afford Zpep('S-acetamide') as a white solid (3.89 mg, 2.85 µmol, 71% yield). The reaction was conducted in parallel with Zpep('SH') library.

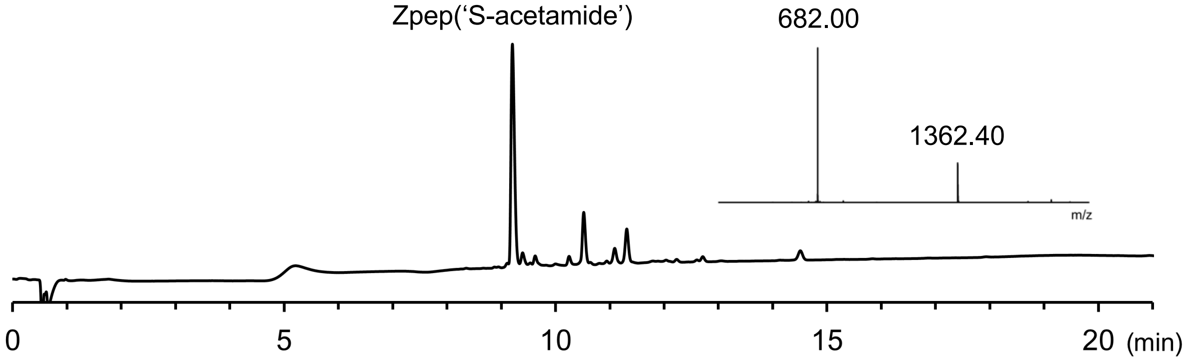

Figure S34. UV chromatogram from LC-MS analysis of the reaction product, with inlaid mass spectrum integrated across the principal UV component. Monoisotopic *m/z* calculated for $[M+H]^+$ 1362.58, found 1362.40.

Acetamide capping of Zpep('SH') library

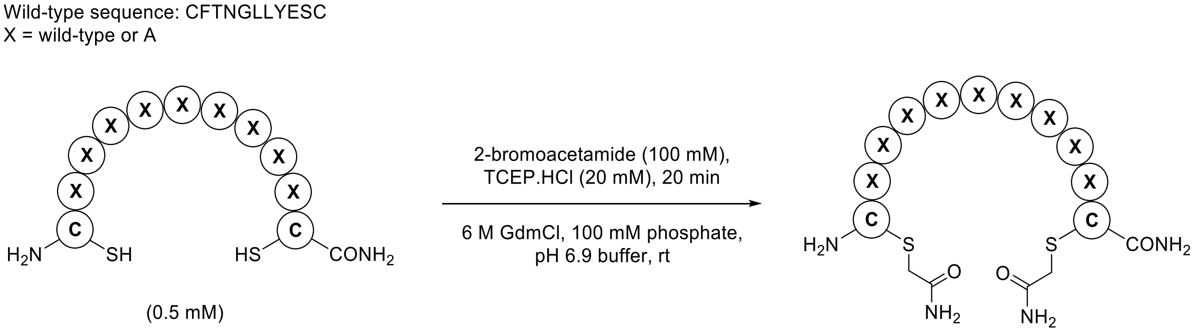

Zpep('SH') library (5 mg, 4.74 µmol total; 9.26 nmol/peptide nominal) was dissolved in 6 M guanidinium chloride, 100 mM phosphate, pH 6.9 buffer (4.74 mL). The reaction buffer from the preceding entry (Page S48) (4.74 mL) was added and left to react for 20 min. The reaction was quenched by the addition of dithiothreitol (292.31 mg, 1.89 mmol), and the resulting solution was subjected to large-scale solid phase extraction (Page S23). The eluates were flash frozen and lyophilized to afford Zpep('S-acetamide') library as a white solid (3.82 mg, 3.26 µmol total; 6.37 nmol/peptide nominal, 69% yield). The reaction was conducted in parallel with Zpep('SH').

Preparation of stock solutions for nLC-MS analysis

The Zpep('S-acetamide') library (3.82 mg, 3.26 µmol total; 6.37 nmol/peptide nominal) was dissolved in 50/50 Solvent A/B (653 µL) to give a nominally 5 mM solution, which was then centrifuged (4,000 rpm, 2 min). The supernatant (2 µL) was diluted in MS grade water (18 µL), and the peptide concentration of the resulting solution was analyzed with a Thermo Scientific™ NanoDrop™ Eight UV-Vis spectrophotometer. Based on the measured peptide concentration, the supernatant of the nominally 5 mM solution was then serially diluted using MS Mobile Phase (2.5% MS grade acetonitrile, 97.5% MS grade water, 0.05% acetic acid) spiked with 2 fmol/peptide/µL Pierce Peptide Retention Time Calibration Mixture to yield 20, 10, and 2 fmol/peptide/µL solutions of the Zpep('S-acetamide') library. The solutions were then analyzed by nLC-MS ('standard run', Page S25).

α,α'-Dibromo-*m*-xylene cyclization of Zpep('SH')

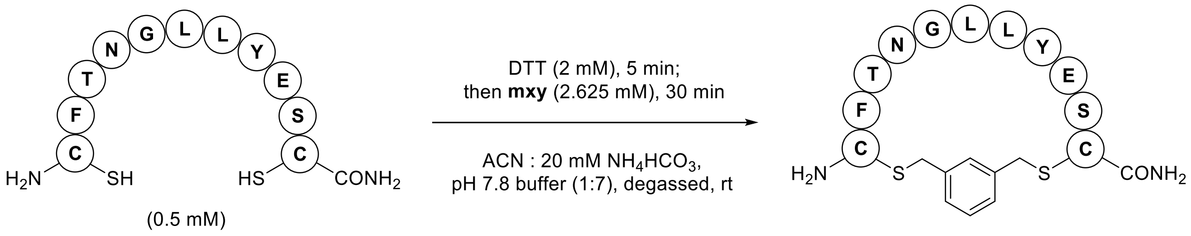

Ammonium bicarbonate (50.59 mg, 0.64 mmol) was dissolved in degassed deionized water (32 mL) to give an aqueous 20 mM ammonium bicarbonate, pH 7.8 buffer. Dithiothreitol (15.43 mg, 0.1 mmol) was dissolved in the ammonium bicarbonate buffer (1 mL) to give a 0.1 M dithiothreitol solution. Zpep('SH') (5 mg, 4.00 µmol) was dissolved in ammonium bicarbonate buffer (6.85 mL). The 0.1 M dithiothreitol solution (160 µL) was added and left to react for 5 min. A t_0 aliquot was taken for LC-MS analysis (7 µL injection volume, 5 µg). 21 mM α,α'-dibromo-*m*-xylene in degassed acetonitrile (1 mL) was then added and left to react for 30 min. The reaction mixture was washed with cold diethyl ether (3x, approx. 8.0 mL each). Excess ether was evaporated over a stream of inert gas, and the solution was filtered through a 0.45 µm nylon filter (NOTE: this was a deviation from the standard procedure, which involved 4-fold dilution into aqueous 6 M guanidinium chloride, 200 mM phosphate, pH 3 buffer, prior to filtration). The filtered solution was subjected to large-scale solid phase extraction (Page S23); but LC-MS analysis indicated the product had been lost during filtration. To recover the product, the 0.45 µm nylon filter was washed with 6 M guanidinium chloride, 100 mM phosphate, pH 6.9 buffer (24 mL). The resulting filtrate was then subjected to large-scale solid phase extraction (Page S23), after combining with the 'loading' fraction of the failed SPE (to approximate the loading conditions of the standard procedure). The eluates were aliquoted for LC-MS analysis (32 µL injection volume, 5 µg, **Figure S35**), flash frozen, and lyophilized to yield Zpep('S-**mxy**-S') as a white solid (5.60 mg, 4.15 µmol, 104% yield). The reaction was conducted in parallel with Zpep('SH') library.

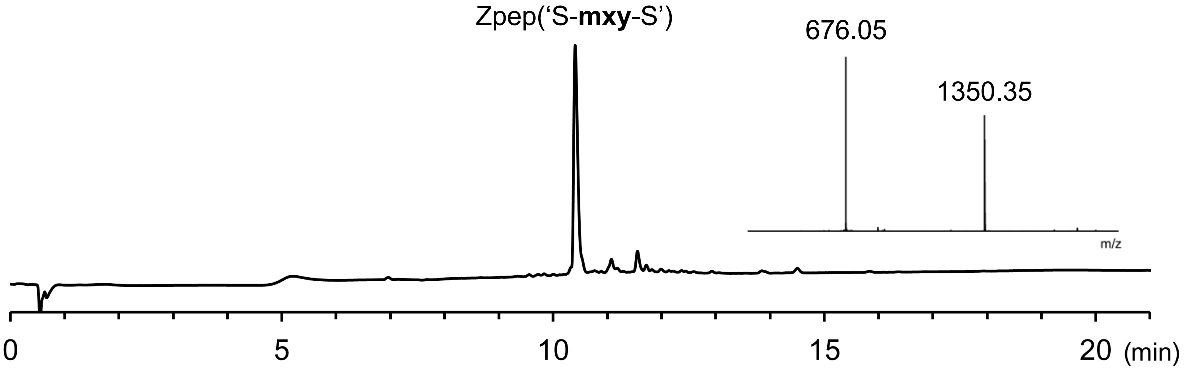

Figure S35. UV chromatogram from LC-MS analysis of the reaction product, with inlaid mass spectrum integrated across the principal UV component. Monoisotopic *m/z* calculated for $[M+H]^+$ 1350.59, found 1350.35.

α,α'-Dibromo-*m*-xylene cyclization of Zpep('SH') library

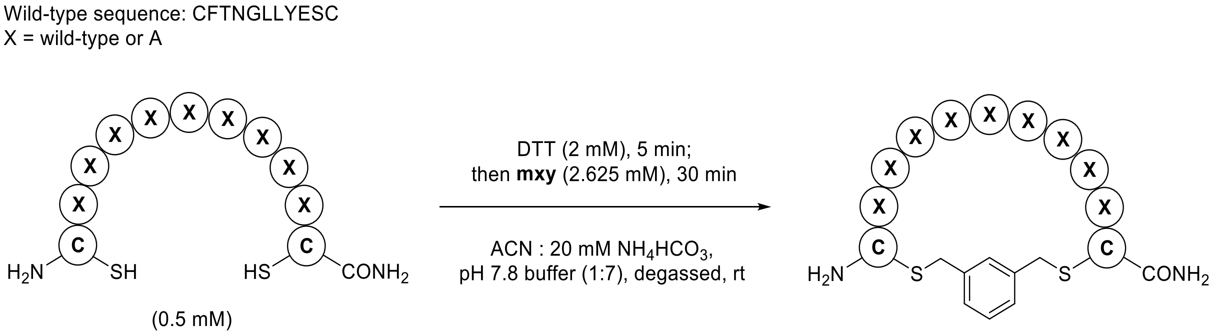

Zpep('SH') library (12 mg, 11.4 µmol total; 22.2 nmol/peptide nominal) was dissolved in aqueous 20 mM ammonium bicarbonate, pH 7.8 buffer from the preceding entry (Page S50) (19.44 mL). The 0.1 M dithiothreitol solution from the preceding entry (Page S50) (0.46 mL) was added to the peptide solution, which was left to react for 5 min. 21 mM α,α'-dibromo-*m*-xylene in degassed acetonitrile (2.84 mL) was then added and left to react for 30 min. The reaction mixture was washed with cold diethyl ether (3x, approx. 22.7 mL each). Excess ether was evaporated over a stream of inert gas, and the solution was filtered through a 0.45 µm nylon filter (NOTE: As above for Zpep('SH'), this was a deviation from the standard procedure, which involved 4-fold dilution into aqueous 6 M guanidinium chloride, 200 mM phosphate, pH 3 buffer, prior to filtration). The filtered solution was subjected to large-scale solid phase extraction (Page S23); but LC-MS analysis indicated the product had been lost during filtration. To recover the product, the 0.45 µm nylon filter was washed with 6 M guanidinium chloride, 100 mM phosphate, pH 6.9 buffer (68 mL). The resulting filtrate was then subjected to large-scale solid phase extraction (Page S23), after combining with the 'loading' fraction of the failed SPE (to approximate the loading conditions of the standard procedure). The eluates were flash frozen and lyophilized to yield Zpep('S-**mxy**-S') library as a white solid (6.02 mg, 5.20 µmol total; 10.2 nmol/peptide nominal, 46% yield). The reaction was conducted in parallel with Zpep('SH').

PITC installation on Zpep('S-mxy-S')

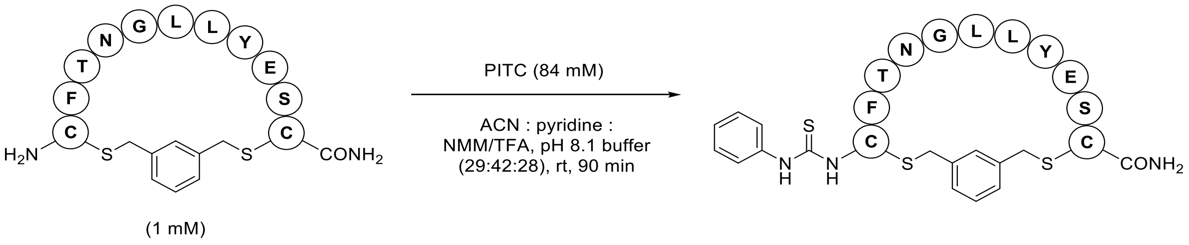

Pyridine (0.92 mL) and pH 8.1 NMM/TFA buffer from Page S32 (0.62 mL) were combined to give the pyr/NMM buffer. Zpep('S-**mxy**-S') (1.44 mg, 1.07 µmol) was dissolved in the pyr/NMM buffer (0.75 mL). An aliquot was diluted 10-fold in Solvent A and analyzed by LC-MS (26 µL injection volume, 5 µg). Phenyl isothiocyanate (22 µL) was diluted in acetonitrile (0.64 mL) to give a 3.33% (v/v) PITC solution, which was added to the peptide solution (0.32 mL). The mixture was then left to react for 90 min, after which it was quenched with Solvent A (9.6 mL). The mixture was centrifuged until the supernatant was clear (4,000 rpm, 15 min) and the supernatant was carefully decanted with a syringe and aliquoted for LC-MS analysis (37 µL injection volume, 5 µg). The supernatants were then washed with diethyl ether (3x, approx. 10.7 mL each). Excess ether was evaporated over a stream of inert gas and the resulting solution was aliquoted for LC-MS analysis (31 µL injection volume, 5 µg). The solutions were subjected to large-scale solid phase extraction (Page S23). Aliquots of the eluates were diluted 4-fold in Solvent A and then subjected to LC-MS analysis (28 µL injection volume, 5 µg, **Figure S36**). Eluates were flash frozen and lyophilized to afford PITC-Zpep('S-**mxy**-S') as a white solid (0.75 mg, 0.50 µmol, 47% yield). The reaction was conducted in parallel with Zpep('SH') library.

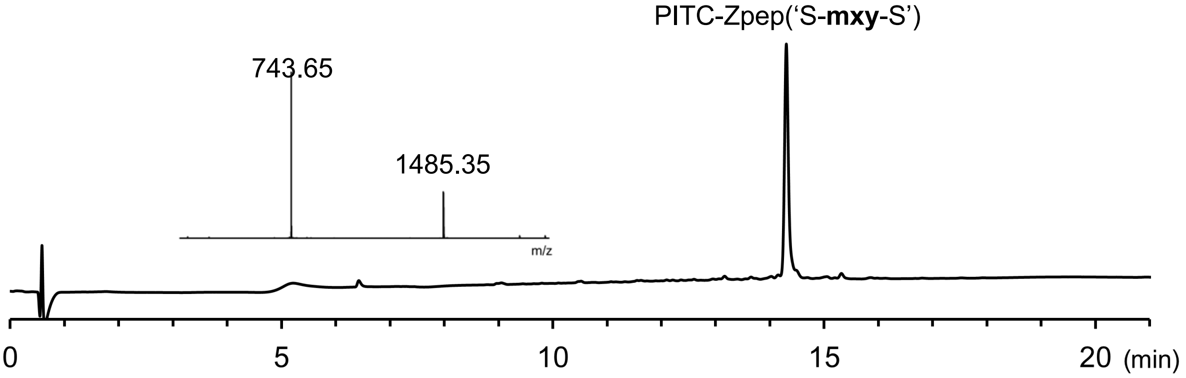

Figure S36. UV chromatogram from LC-MS analysis of the reaction product, with inlaid mass spectrum integrated across the principal UV component. Monoisotopic *m/z* calculated for $[M+H]^+$ 1485.60, found 1485.35.

PITC installation on Zpep('S-mxy-S') library

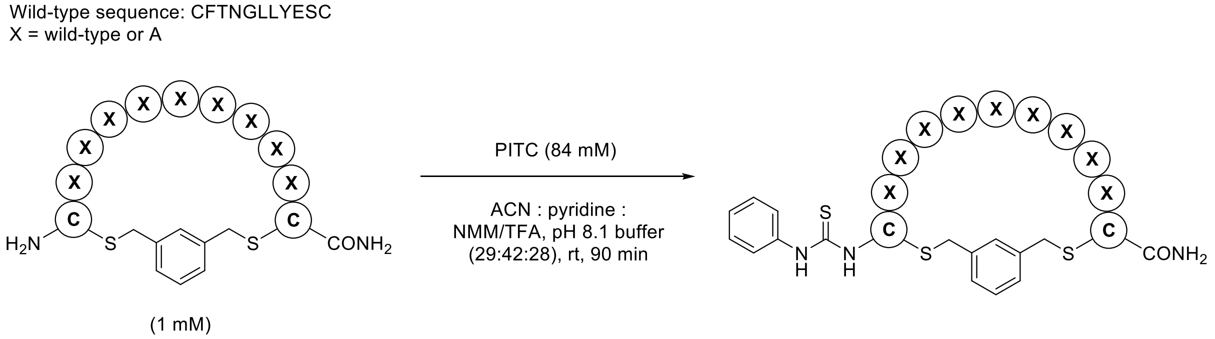

Zpep('S-**mxy**-S') library (1.14 mg, 0.98 µmol total; 1.91 nmol/peptide nominal) was dissolved in the pyr/NMM buffer from the preceding entry (Page S52) (0.69 mL). An aliquot was diluted 10-fold in Solvent A and analyzed by LC-MS (30 µL injection volume, 5 µg). The 3.33% (v/v) PITC solution from the preceding entry (Page S52) was added to the peptide solutions (0.3 mL). The mixture was then left to react for 90 min, after which it was quenched with Solvent A (8.86 mL). The mixture was centrifuged until the supernatant was clear (4,000 rpm, 15 min) and the supernatant was carefully decanted with a syringe and aliquoted for LC-MS analysis (40 µL injection volume, 4.65 µg). The supernatants were then washed with diethyl ether (3x, approx. 9.8 mL each). Excess ether was evaporated over a stream of inert gas and the resulting solutions were aliquoted for LC-MS analysis (31 µL injection volume, 5 µg). The solutions were subjected to large-scale solid phase extraction (Page S23). Aliquots of the eluates were diluted 4-fold in Solvent A and then subjected to LC-MS analysis (35 µL injection volume, 5 µg). Eluates were flash frozen and lyophilized to afford PITC-Zpep('S-**mxy**-S') library as a white solid (0.34 mg, 0.26 µmol total; 0.51 nmol/peptide nominal, 27% yield, **Figure S37**). The reaction was conducted in parallel with Zpep('SH').

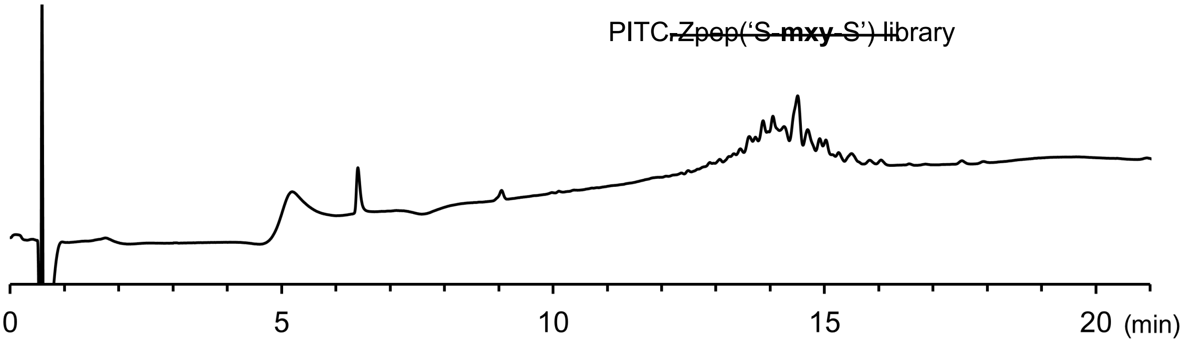

Figure S37. UV chromatogram of the reaction product.

Edman linearization of PITC-Zpep('S-mxy-S')

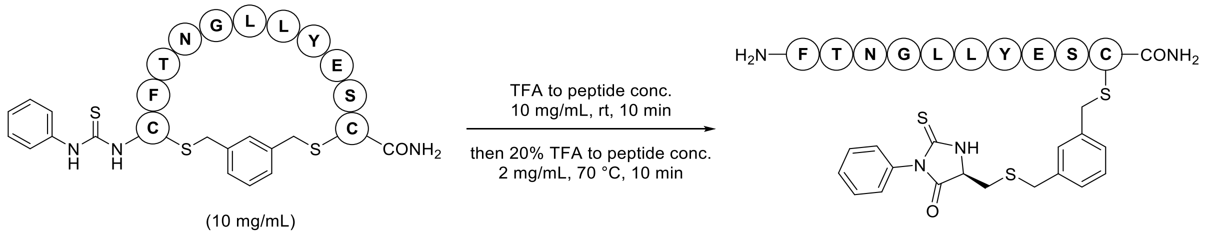

PITC-Zpep('S-**mxy**-S') (0.75 mg, 0.50 µmol) was dissolved in trifluoroacetic acid (75 µL) and left to react for 10 min. The solution was diluted 5-fold with deionized water (0.3 mL) and heated to 70 °C for 10 min. The solution was then diluted with aqueous 6 M guanidinium chloride, 100 mM phosphate, pH 7 buffer (1.13 mL), and subjected to small-scale solid phase extraction (Page S23). Aliquots of the eluates were diluted 10-fold in Solvent A and subjected to LC-MS analysis (13 µL injection volume, 5 µg, **Figure S38**), and the eluates were flash frozen and lyophilized to afford linearized Zpep('S-**mxy**-S') as a white solid (0.66 mg, 0.44 µmol, 88% yield). The reaction was conducted in parallel with PITC-Zpep('S-**mxy**-S') library.

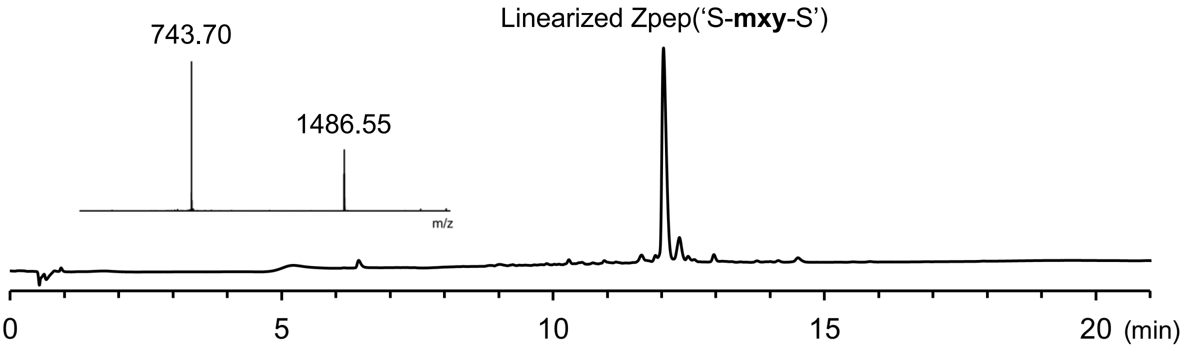

Figure S38. UV chromatogram from LC-MS analysis of the reaction product, with inlaid mass spectrum integrated across the principal UV component. Monoisotopic *m/z* calculated for $[M+H]^+$ 1485.60, found 1486.55.

Edman linearization of PITC-Zpep('S-mxy-S') library

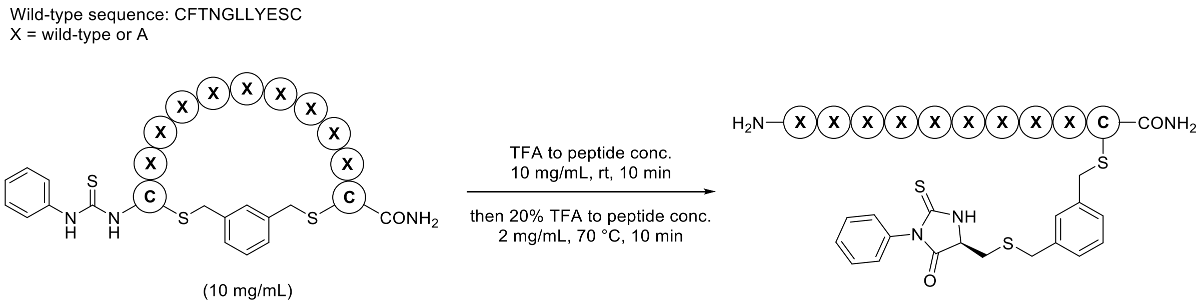

PITC-Zpep('S-**mxy**-S') library (0.34 mg, 0.26 µmol total; 0.51 nmol/peptide nominal) was dissolved in trifluoroacetic acid (34 µL) and left to react for 10 min. The solution was diluted 5-fold with deionized water (136 µL) and heated to 70 °C for 10 min. The solution was then diluted with aqueous 6 M guanidinium chloride, 100 mM phosphate, pH 7 buffer (0.51 mL), and subjected to small-scale solid phase extraction (Page S23). Aliquots of the eluates were diluted 10-fold in Solvent A and subjected to LC-MS analysis (29 µL injection volume, 5 µg, **Figure S39**), and the eluates were flash frozen and lyophilized to afford linearized Zpep('S-**mxy**-S') library as a white solid (0.14 mg, 0.11 µmol total; 0.21 nmol/peptide nominal, 41% yield). The reaction was conducted in parallel with PITC-Zpep('S-**mxy**-S').

Preparation of stock solutions for nLC-MS analysis

The linearized Zpep('S-**mxy**-S') library (0.14 mg, 0.11 µmol total; 0.21 nmol/peptide nominal) was dissolved in 50/50 Solvent A/B (21.7 µL) to give a nominally 5 mM solution, which was then centrifuged (4,000 rpm, 2 min). The supernatant (2 µL) was diluted in MS grade water (18 µL), and the peptide concentration of the resulting solution was analyzed with a Thermo Scientific™ NanoDrop™ Eight UV-Vis spectrophotometer, which gave calculated results exceeding the nominal peptide concentration. Hence, based on the nominal peptide concentration, the supernatant of the nominally 5 mM solution was then serially diluted using MS Mobile Phase (2.5% MS grade acetonitrile, 97.5% MS grade water, 0.05% acetic acid) spiked with 2 fmol/peptide/µL Pierce Peptide Retention Time Calibration Mixture to yield 20, 10, and 2 fmol/peptide/µL solutions of the linearized Zpep('S-**mxy**-S') library. The solutions were then analyzed by nLC-MS ('standard run', Page S25).

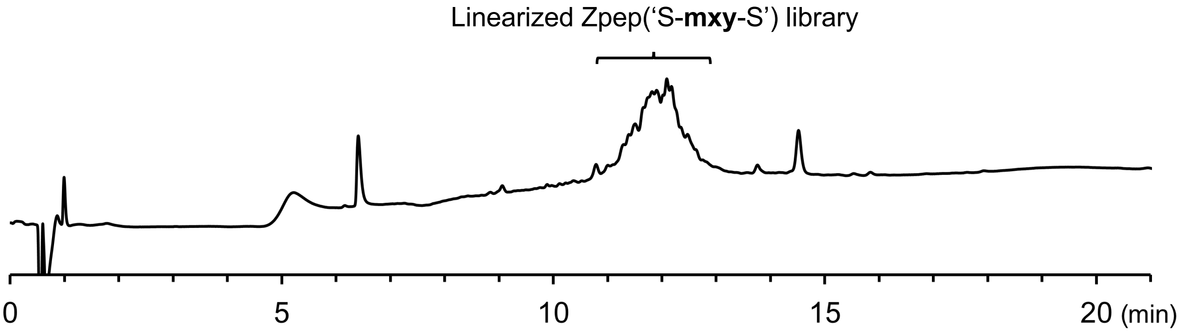

Figure S39. UV chromatogram of the reaction product.

Pentafluorophenyl sulfide cyclization of Zpep('SH')

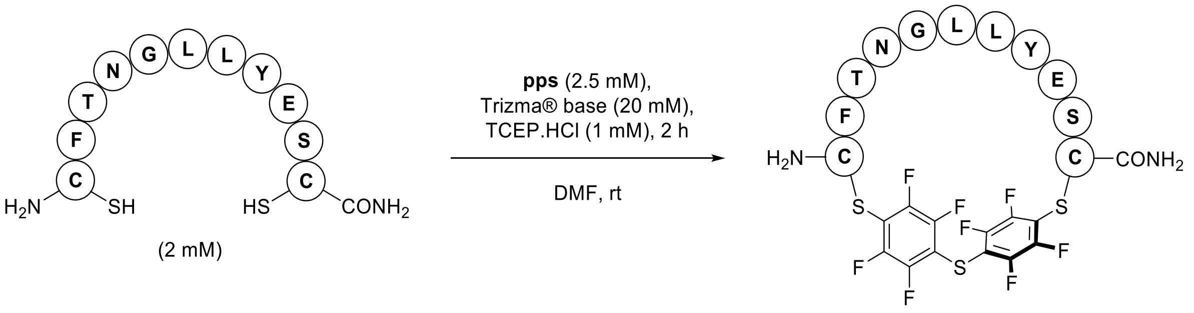

Zpep('SH') (5 mg, 4.00 µmol) was dissolved in dimethyl formamide (0.85 mL). 50 mM Trizma® base in dimethyl formamide (0.8 mL) and 20 mM tris(2-carboxyethyl)phosphine hydrochloride in dimethyl formamide (100 µL) were added to the solution, and a t_0 aliquot was diluted 20-fold in Solvent A for LC-MS analysis (35 µL injection volume, 5 µg). 20 mM pentafluorophenyl sulfide in dimethyl formamide (0.25 mL) was added, and the mixture was left to react for 2 h. The solution was then diluted 20-fold with Solvent A (38 mL), aliquoted for LC-MS analysis (40 µL injection volume, 5 µg), and subjected to large-scale solid phase extraction (Page S23). Aliquots of the eluates were diluted 10-fold with aqueous 6 M guanidinium chloride, 100 mM phosphate, pH 2 buffer for LC-MS analysis (20 µL injection volume, 5 µg, **Figure S40**). The eluates were flash frozen and lyophilized to afford Zpep('S-**pps**-S') as a white solid (3.93 mg, 2.50 µmol, 62% yield). The reaction was conducted in parallel with Zpep('SH') library.

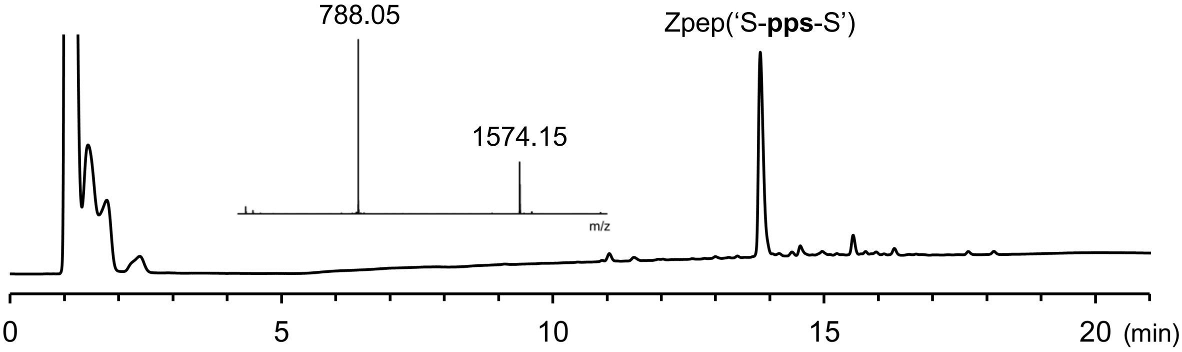

Figure S40. UV chromatogram from LC-MS analysis of the reaction product, with inlaid mass spectrum integrated across the principal UV component. Monoisotopic *m/z* calculated for $[M+H]^+$ 1574.48, found 1574.15.

Pentafluorophenyl sulfide cyclization of Zpep('SH') library

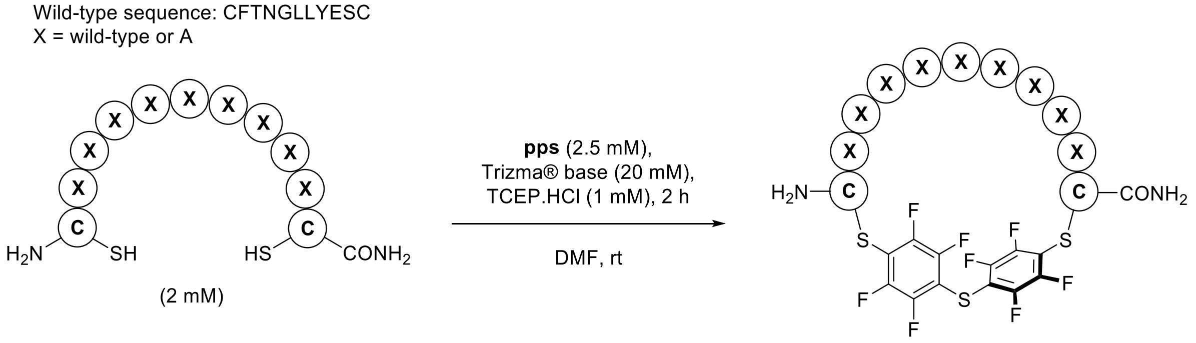

Zpep('SH') library (12 mg, 11.4 μmol total; 22.2 nmol/peptide nominal) was dissolved in dimethyl formamide (2.42 mL). 50 mM Trizma® base in dimethyl formamide (2.27 mL), 20 mM tris(2-carboxyethyl)phosphine hydrochloride in dimethyl formamide (0.28 mL), and 20 mM pentafluorophenyl sulfide in dimethyl formamide (0.71 mL) were added to the peptides, and the solutions were left to react for 2 h. The solution was then diluted 20-fold with Solvent A (108 mL) and subjected to large-scale solid phase extraction (Page S23). The eluates were flash frozen and lyophilized to afford Zpep('S-**pps**-S') library as a white solid (12.27 mg, 8.88 μmol total; 17.3 nmol/peptide nominal, 78% yield). The reaction was conducted in parallel with Zpep('SH').

PITC installation on Zpep('S-pps-S')

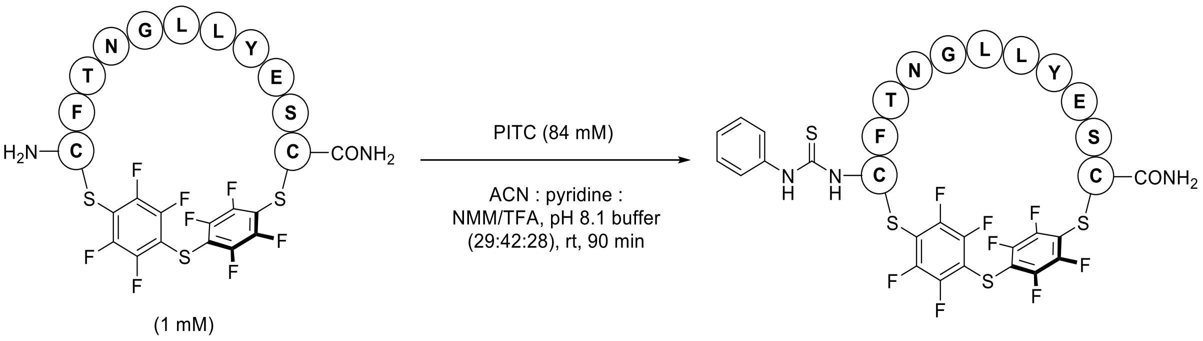

Pyridine (1.47 mL) was combined with the pH 8.1 NMM/TFA buffer from Page S32 (0.98 mL). KRpep('S-**pps**-S') (1.5 mg, 0.95 µmol) was dissolved in the pyridine-buffer mixture (0.67 mL), and a t_0 aliquot was diluted 10-fold in Solvent A for LC-MS analysis (22 µL injection volume, 5 µg). Phenyl isothiocyanate (35 µL) was diluted in acetonitrile (1.02 mL) to give a 3.33% (v/v) PITC solution. The PITC solution (0.29 mL) was then added to the peptide solution and left to react for 90 min. The reaction mixture was diluted 10-fold by addition of Solvent A (8.58 mL) and centrifuged until the supernatant was clear (4,000 rpm, 30 min). The supernatant was carefully decanted with a syringe and aliquoted for LC-MS analysis (32 µL injection volume, 5 µg). The supernatant was washed with diethyl ether (3x, approx. 9.5 mL each) and excess ether was evaporated over a stream of inert gas. An aliquot was taken for LC-MS analysis (32 µL injection volume, 5 µg). The solution was subjected to large-scale solid phase extraction (Page S23), and aliquots of the eluates were diluted 5-fold in Solvent A for LC-MS analysis (33 µL injection volume, 5 µg, **Figure S41**). The eluates were flash frozen and lyophilized to afford PITC-KRpep('S-**pps**-S') as a white solid (0.16 mg, 93.6 nmol, 10% yield). The reaction was conducted in parallel with Zpep('S-**pps**-S') library.

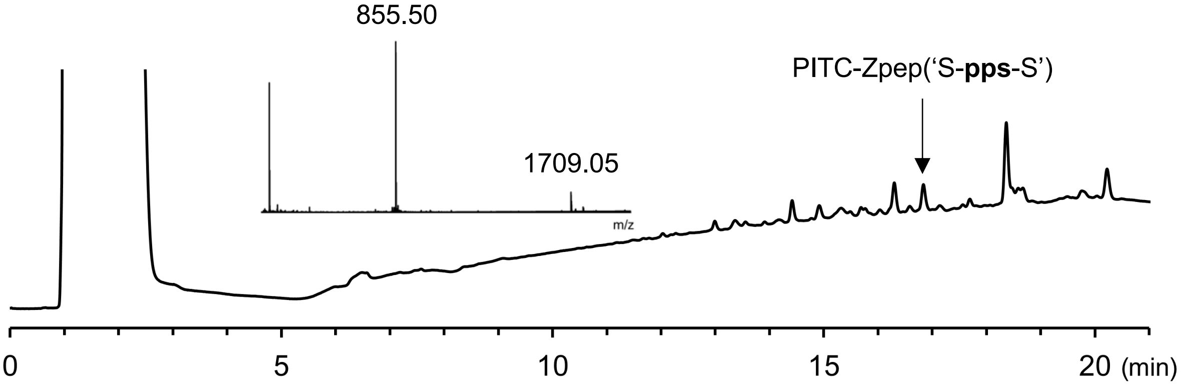

Figure S41. UV chromatogram from LC-MS analysis of the reaction product, with inlaid mass spectrum integrated across the principal UV component. Monoisotopic *m/z* calculated for $[M+H]^+$ 1709.50, found 1709.05.

PITC installation on Zpep('S-pps-S') library

Wild-type sequence: CFTNGLLYESC
X = wild-type or A

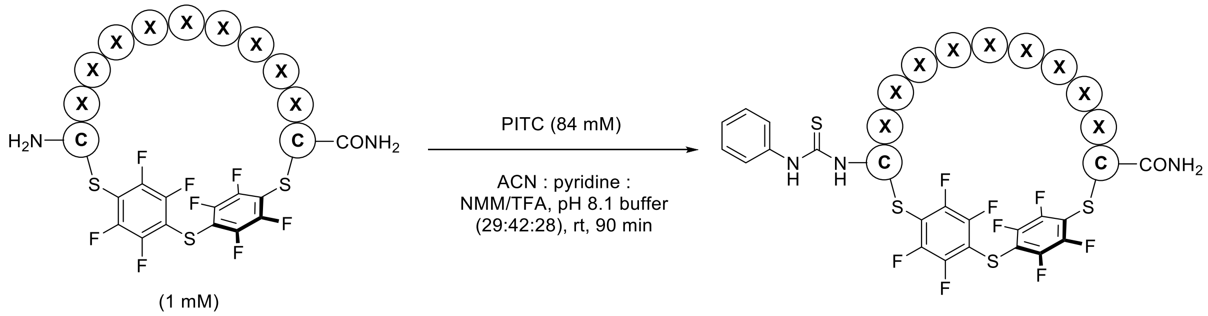

Zpep('S-**pps**-S') library (3 mg, 2.17 µmol total; 4.24 nmol/peptide nominal) was dissolved in the pyridine-buffer mixture from the preceding entry (Page S58) (1.52 mL), and a t_0 aliquot was diluted 10-fold in Solvent A for LC-MS analysis (25 µL injection volume, 5 µg). The PITC solution from the preceding entry (Page S58) (0.65 mL) was then added to the peptide solution and left to react for 90 min. The reaction mixture was diluted 10-fold by addition of Solvent A (19.54 mL) and centrifuged until the supernatant was clear (4,000 rpm, 45 min). The supernatant was carefully decanted with a syringe and aliquoted for LC-MS analysis (36 µL injection volume, 5 µg). The supernatant was washed with diethyl ether (3x, approx. 21.7 mL each) and excess ether was evaporated over a stream of inert gas. The solution was aliquoted for LC-MS analysis (36 µL injection volume, 5 µg), and subjected to large-scale solid phase extraction (Page S23). Aliquots of the eluates were diluted 5-fold in Solvent A for LC-MS analysis (17 µL injection volume, 5 µg, **Figure S42**). The eluates were flash frozen and lyophilized to afford PITC-Zpep('S-**pps**-S') library as a white solid (0.72 mg, 0.42 µmol total; 0.82 nmol/peptide nominal, 22% yield). The reaction was conducted in parallel with Zpep('S-**pps**-S').

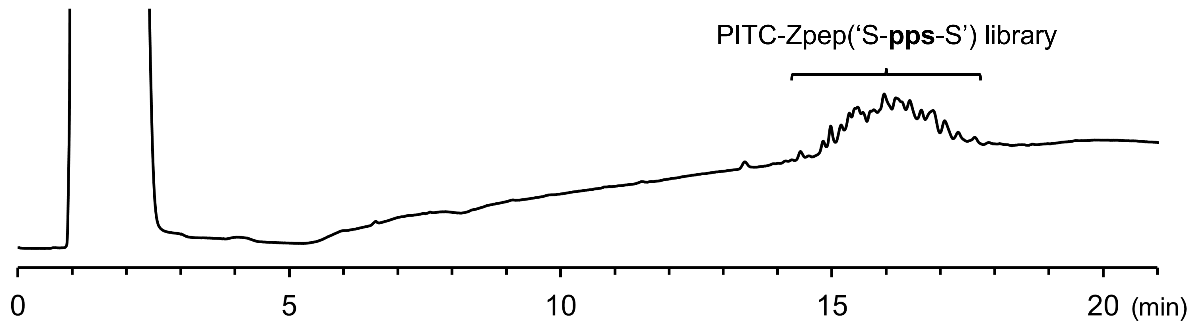

Figure S42. UV chromatogram of the reaction product.

Attempted diiodomethane cyclization of Zpep('SH') (original conditions)

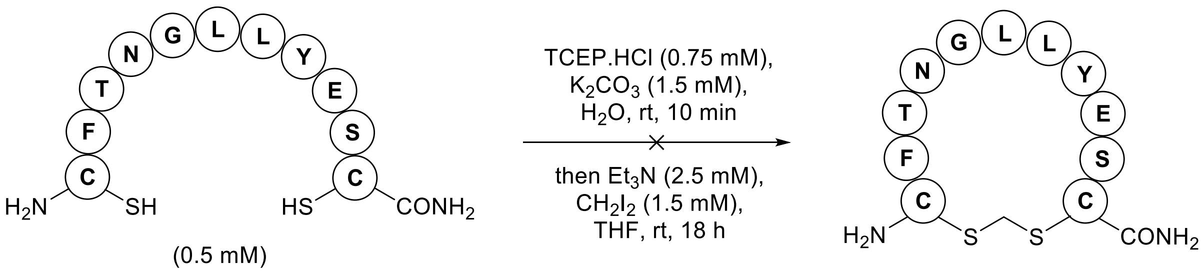

Zpep('SH') (0.1 mg, 80.1 nmol) was dissolved in 5 mM aqueous TCEP.HCl (24 µL), 5 mM aqueous potassium carbonate (48 µL), and deionized water (75 µL). A t_0 aliquot was taken, diluted 5-fold in aqueous 6 M guanidinium chloride, 100 mM phosphate, pH 2 buffer, and analyzed by LC-MS (37 µL injection volume, 5 µg). 50 mM triethylamine in THF (8 µL) and 50 mM diiodomethane in THF (4.8 µL) were added to the peptide solutions. At t = 30 min and t = 18 h, aliquots were taken, diluted as above, and analyzed by LC-MS (40 µL injection volume, 5 µg, **Figure S43**). The product was not isolated. The reaction was conducted in parallel with KRpep('SH').

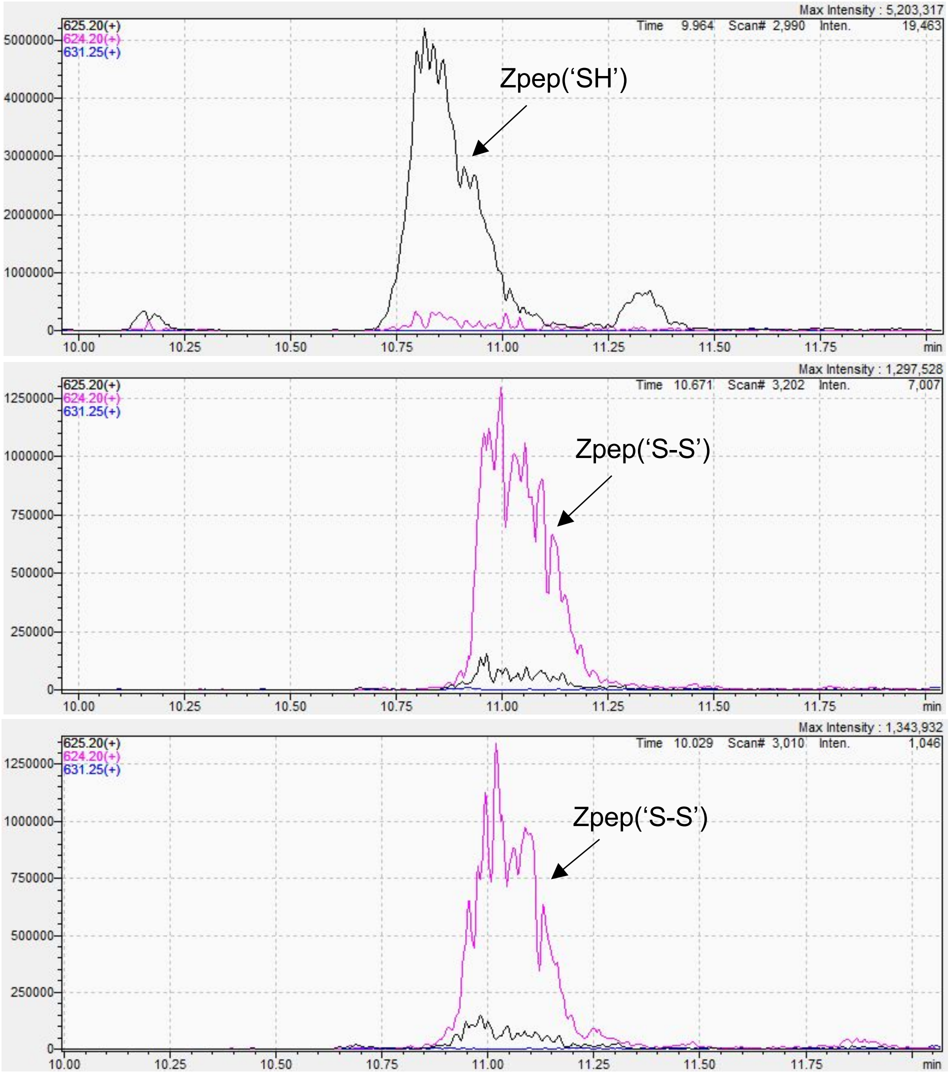

Figure S43. Extracted ion chromatograms of the reaction mixture at t = 0 (top), at t = 30 min (middle), and t = 18 h (bottom). Traces corresponding to Zpep('SH'), Zpep('S-S'), and Zpep('S-CH_2-S') are in black, magenta, and blue respectively.

Attempted diiodomethane cyclization of Zpep('SH') (adapted mxy cyclisation conditions)

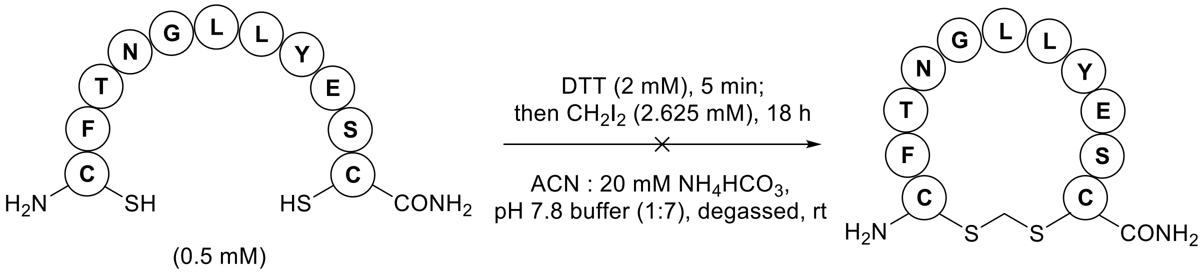

Zpep('SH') (0.1 mg, 80.1 nmol) was dissolved in the ammonium bicarbonate buffer (137 µL) and 0.1 M dithiothreitol solution (3.2 µL) from Page S46. t_0 aliquots were diluted 5-fold in aqueous 6 M guanidinium chloride, 100 mM phosphate, pH 2 buffer, and analyzed by LC-MS (35 µL injection volume, 5 µg). Degassed acetonitrile (17.1 µL), followed by 144 mM diiodomethane solution (2.9 µL) were then added to the peptide solution. At t = 30 min and t = 18 h, aliquots were taken, diluted as above, and analyzed by LC-MS (40 µL injection volume, 5 µg, **Figure S44**). The product was not isolated. The reaction was conducted in parallel with KRpep('SH').

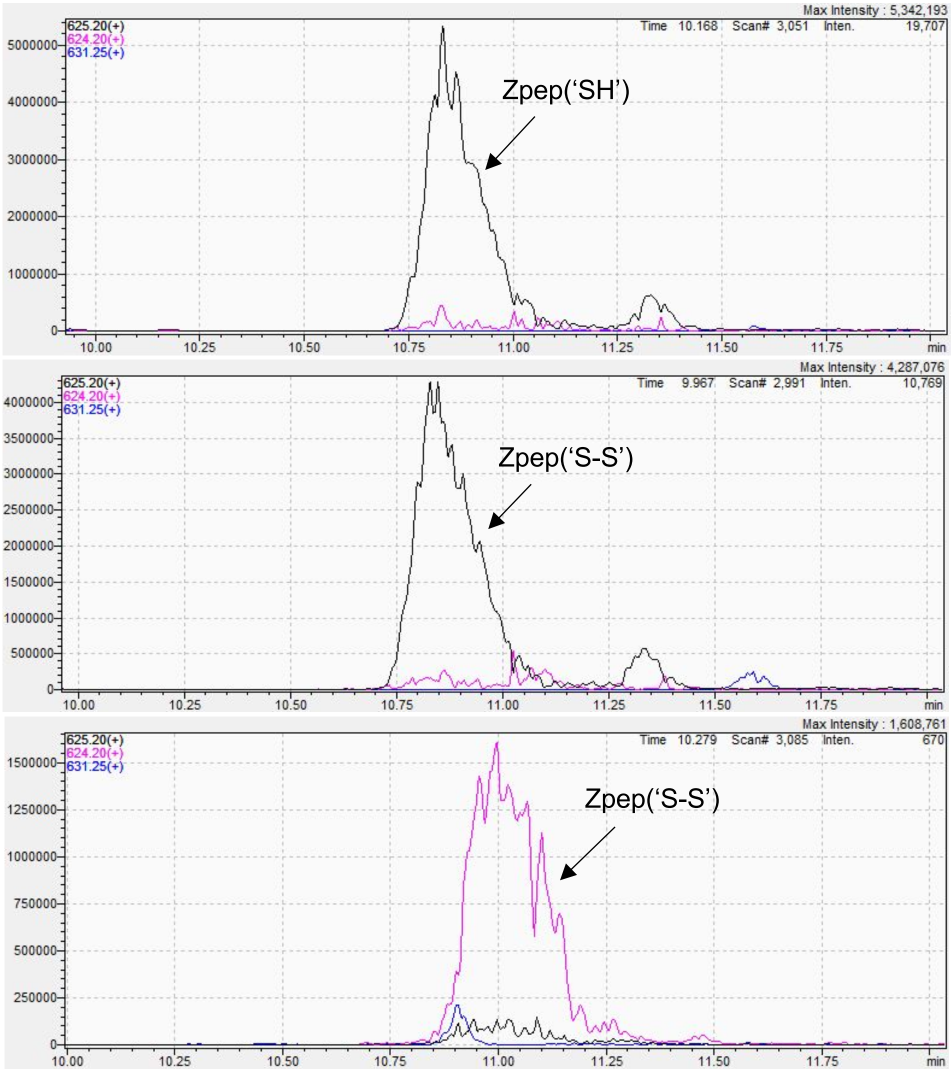

Figure S44. Extracted ion chromatograms of the reaction mixture at t = 0 (top), at t = 30 min (middle), and t = 18 h (bottom). Traces corresponding to Zpep('SH'), Zpep('S-S'), and Zpep('S-CH_2-S') are in black, magenta, and blue respectively.

Attempted diiodomethane cyclization of Zpep(‘SH’) (A*STAR conditions)

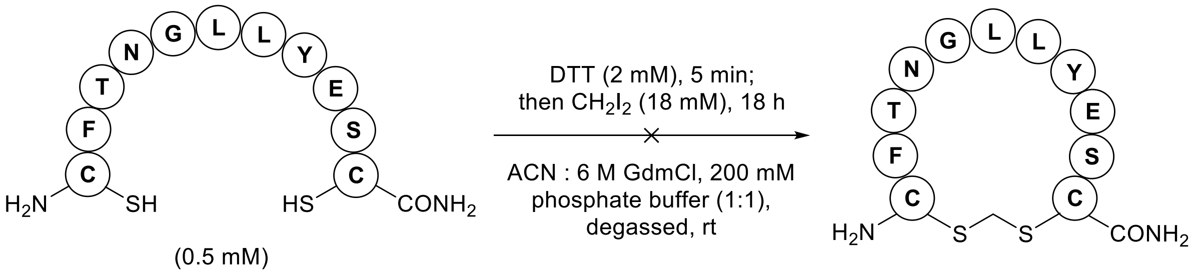

Zpep(‘SH’) (0.1 mg, 80.1 nmol) was dissolved in the 100 mM dithiothreitol solution from Page S46 (3.2 µL) and 6 M guanidinium chloride, 200 mM phosphate, pH 9 buffer (77 µL) and the mixture was left to react for 5 min. A t_0 aliquot was diluted 5-fold in 6 M guanidinium chloride, 100 mM phosphate, pH 2 buffer for LC-MS analysis (20 µL, 5 µg). 36 mM diiodomethane in degassed acetonitrile (80 µL) was then added to the mixture. At t = 30 min and t = 18 h, aliquots were taken, diluted as above, and analyzed by LC-MS (40 µL injection volume, 5 µg, **Figure S45**). The product was not isolated. The reaction was conducted in parallel with KRpep(‘SH’), Ac-KRpep(‘SH’), and Ac-Zpep(‘SH’).

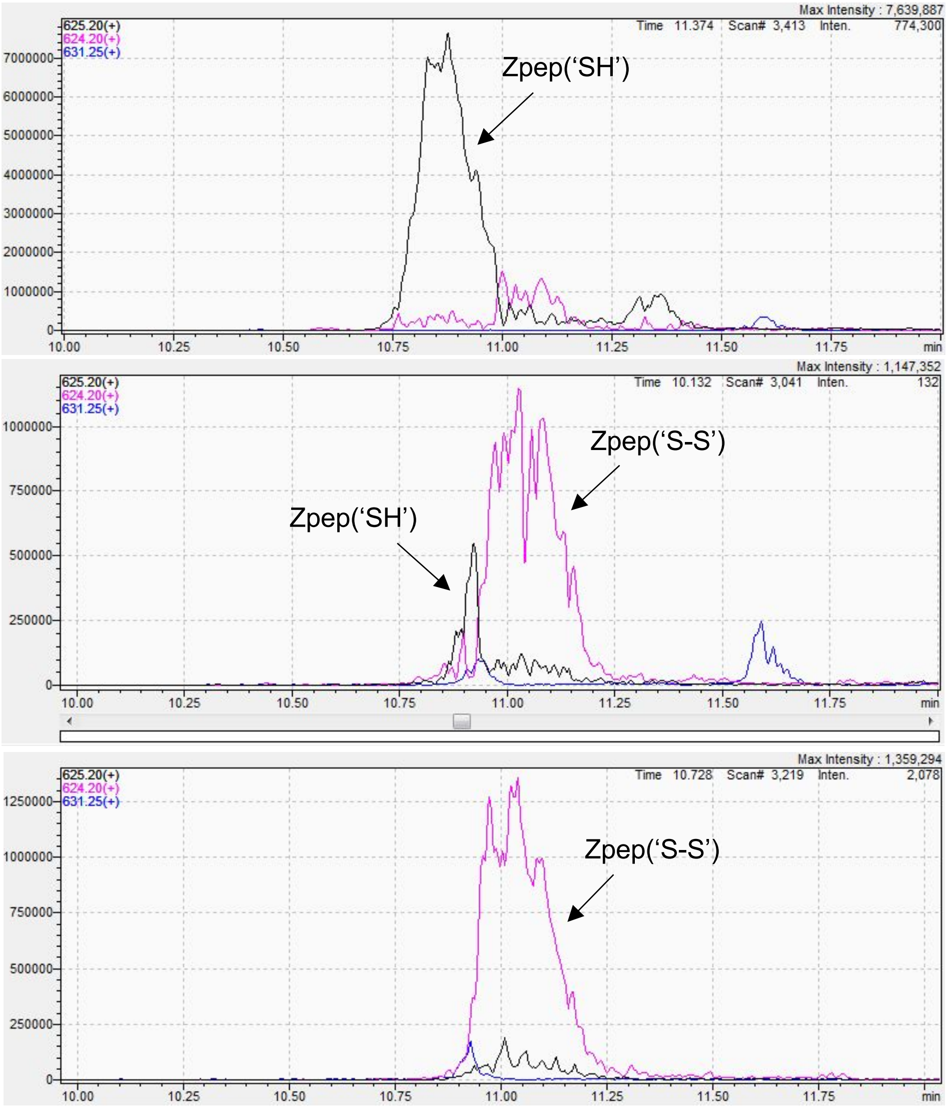

Figure S45. Extracted ion chromatograms of the reaction mixture at t = 0 (top), at t = 30 min (middle), and t = 18 h (bottom). Traces corresponding to Zpep('SH'), Zpep('S-S'), and Zpep('S-CH_2-S') are in black, magenta, and blue respectively.

N-terminus acetylation of 'KRpep' peptidyl-resin

Preparation of 'capping solution'

Acetic anhydride (0.8 mL), dimethyl formamide (0.8 mL), and diisopropylethyl amine (0.4 mL) were combined to form the 'capping solution'.

Reaction

'KRpep' peptidyl-resin (50 mg, 8.03 µmol) was washed with DMF and drained. The 'capping solution' (1 mL) was added to the peptidyl-resin and left to react for 30 min. The solution was then drained and the resulting peptidyl-resin was washed with DMF (5 x 2 mL), followed by DCM (10 x 1 mL), and dried under N_2 to afford Ac-'KRpep' peptidyl-resin as a pale yellow solid (46.90 mg, 7.49 µmol, 93% yield).

Preparative cleavage/side chain deprotection was conducted as per Page S20. Aliquots of the eluates after large-scale solid phase extraction (Page S23) were diluted 40-fold in Solvent A for LC-MS analysis (30 µL injection volume, 5 µg, **Figure S46**). The eluates were flash frozen and lyophilized to afford Ac-KRpep('SH') as a white solid (3.91 mg, 2.98 µmol, 40% yield). Both reactions were conducted in parallel with Zpep('SH').

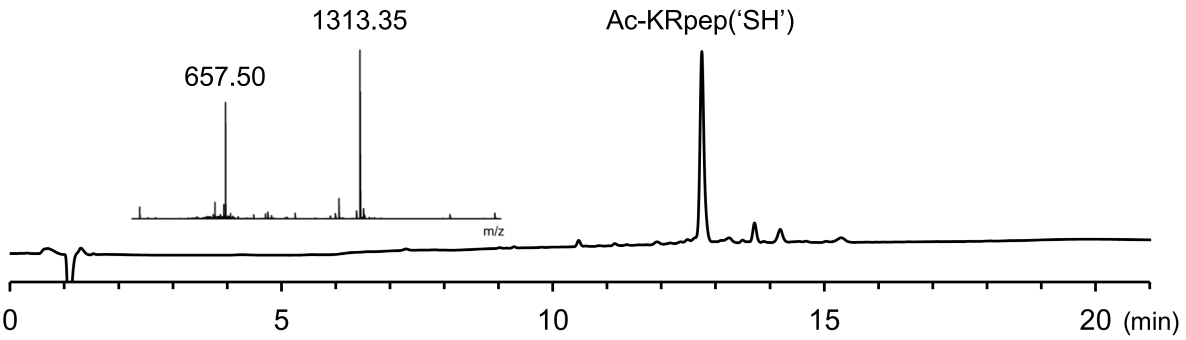

Figure S46. UV chromatogram from LC-MS analysis of the reaction product, with inlaid mass spectrum integrated across the principal UV component. Monoisotopic *m/z* calculated for $[M+H]^+$ 1313.59, found 1313.35.

N-terminus acetylation of 'Zpep' peptidyl-resin

'Zpep' peptidyl-resin (50 mg, 7.69 µmol) was washed with DMF and drained. The 'capping solution' from the preceding entry (Page S66) (1 mL) was added to the peptidyl-resin and left to react for 30 min. The solution was then drained and the resulting peptidyl-resin was washed with DMF (5 x 2 mL), followed by DCM (10 x 1 mL), and dried under N_2 to afford Ac-'Zpep' peptidyl-resin as a pale yellow solid (50.33 mg, 7.69 µmol, 100% yield).

Preparative cleavage/side chain deprotection was conducted as per the preceding entry (Page S66). Aliquots of the eluates after large-scale solid phase extraction (Page S23) were diluted 40-fold in Solvent A for LC-MS analysis (30 µL injection volume, 5 µg, **Figure S47**). The eluates were flash frozen and lyophilized to afford Ac-Zpep('SH') as a white solid (4.70 mg, 3.64 µmol, 45% yield). Both reactions were conducted in parallel with KRpep('SH').

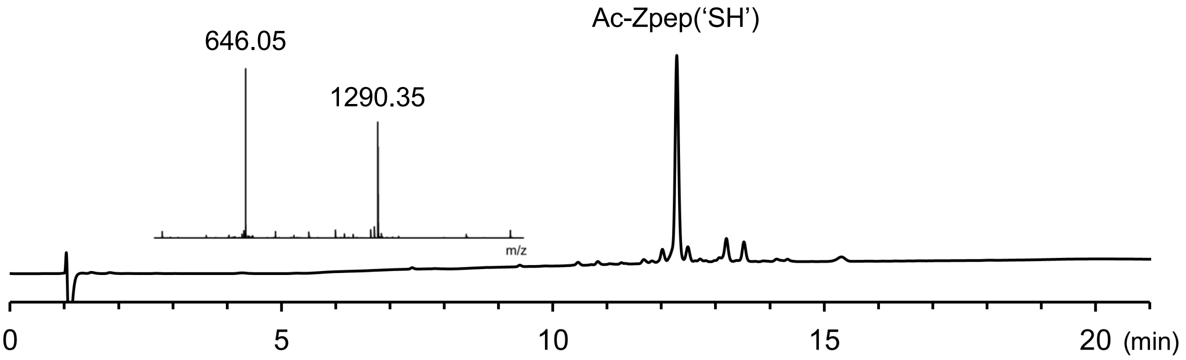

Figure S47. UV chromatogram from LC-MS analysis of the reaction product, with inlaid mass spectrum integrated across the principal UV component. Monoisotopic *m/z* calculated for $[M+H]^+$ 1290.55, found 1290.35.

Attempted diiodomethane cyclization of Ac-KRpep('SH') (adapted mxy cyclisation conditions)

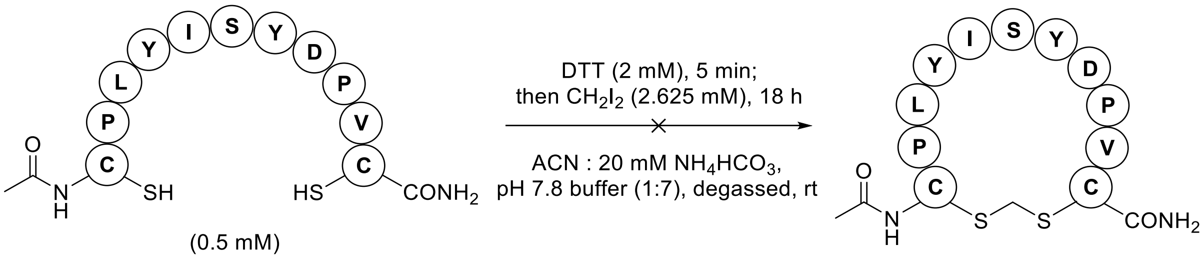

Preparation of buffer

Ammonium bicarbonate (7.91 mg, 0.1 mmol) was dissolved in degassed deionized water (5 mL) to give an aqueous 20 mM ammonium bicarbonate pH 7.8 buffer.

Preparation of dithiothreitol solution

Dithiothreitol (7.72 mg, 0.05 mmol) was dissolved in the ammonium bicarbonate buffer (0.5 mL) to give a 0.1 M dithiothreitol solution.

Preparation of diiodomethane solution in acetonitrile

Diiodomethane (5.80 µL, 0.072 mmol) was dissolved in degassed acetonitrile (0.5 mL) to give a 144 mM diiodomethane solution.

Reaction

Ac-KRpep('SH') (0.1 mg, 76.1 nmol) was dissolved in the ammonium bicarbonate buffer (130 µL) and 0.1 M dithiothreitol solution (3.1 µL). t_0 aliquots were diluted 5-fold in aqueous 6 M guanidinium chloride, 100 mM phosphate, pH 2 buffer, and analyzed by LC-MS (33 µL injection volume, 5 µg). Degassed acetonitrile (16.3 µL), followed by 144 mM diiodomethane solution (2.8 µL) were then added to the peptide solution. At t = 30 min and t = 18 h, aliquots were taken, diluted as above, and analyzed by LC-MS (38 µL injection volume, 5 µg, **Figure S48**). The product was not isolated. The reaction was conducted in parallel with Ac-Zpep('SH').

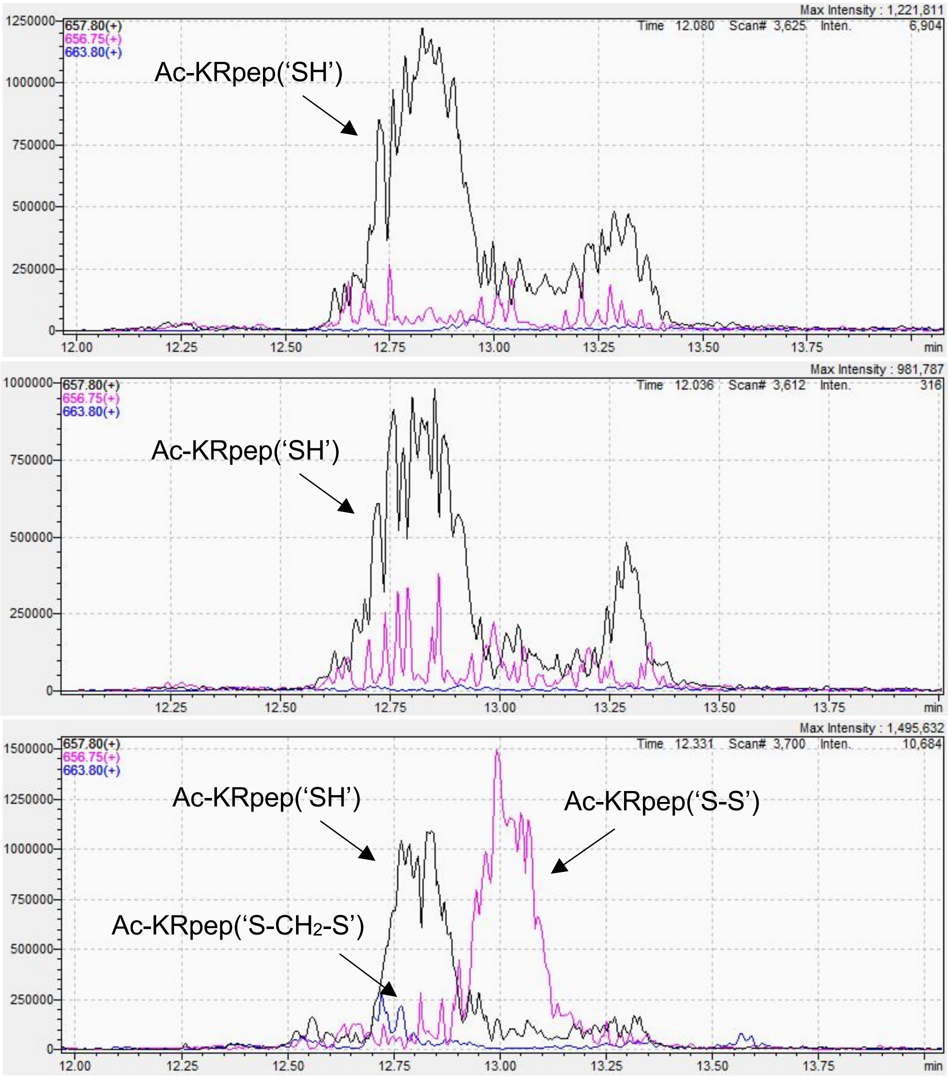

Figure S48. Extracted ion chromatograms of the reaction mixture at t = 0 (top), at t = 30 min (middle), and t = 18 h (bottom). Traces corresponding to Ac-KRpep('SH'), Ac-KRpep('S-S'), and Ac-KRpep('S-CH_2-S') are in black, magenta, and blue respectively.

__**Attempted diiodomethane cyclization of Ac-Zpep('SH') (adapted mxy cyclisation conditions)**__

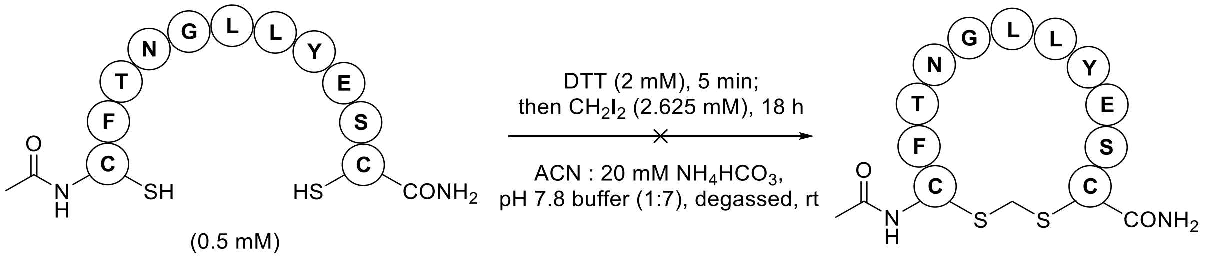

Ac-Zpep('SH') (0.1 mg, 7.75 nmol) was dissolved in the ammonium bicarbonate buffer (133 µL) and 0.1 M dithiothreitol solution (3.1 µL) from Page S68. t_0 aliquots were diluted 5-fold in aqueous 6 M guanidinium chloride, 100 mM phosphate, pH 2 buffer, and analyzed by LC-MS (34 µL injection volume, 5 µg). Degassed acetonitrile (16.6 µL), followed by 144 mM diiodomethane solution (2.8 µL) were then added to the peptide solution. At t = 30 min and t = 18 h, aliquots were taken, diluted as above, and analyzed by LC-MS (39 µL injection volume, 5 µg, **Figure S49**). The product was not isolated. The reaction was conducted in parallel with Ac-KRpep('SH').

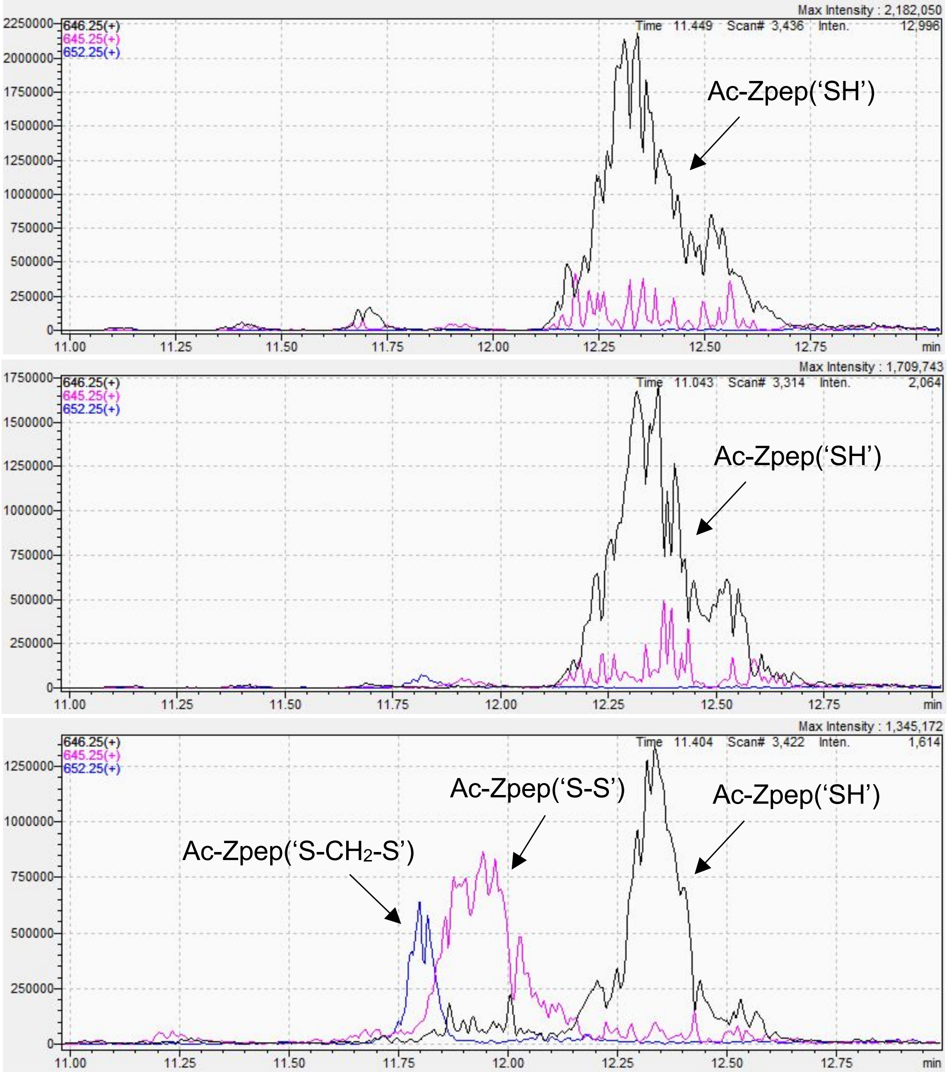

Figure S49. Extracted ion chromatograms of the reaction mixture at t = 0 (top), at t = 30 min (middle), and t = 18 h (bottom). Traces corresponding to Ac-Zpep('SH'), Ac-Zpep('S-S'), and Ac-Zpep('S-CH_2-S') are in black, magenta, and blue respectively.

Diiodomethane cyclization of Ac-KRpep(‘SH’) (A*STAR conditions)

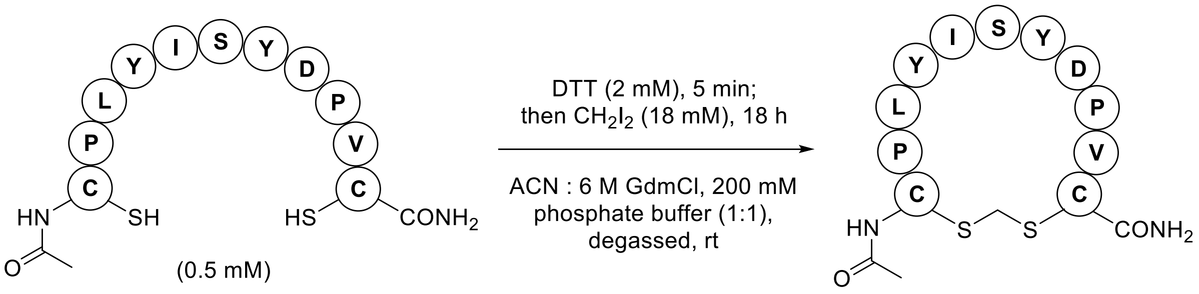

Ac-KRpep(‘SH’) (0.1 mg, 80.1 nmol) was dissolved in the 100 mM dithiothreitol solution from Page S46 (3.1 µL) and 6 M guanidinium chloride, 200 mM phosphate, pH 9 buffer (73 µL) and the mixture was left to react for 5 min. A t_0 aliquot was diluted 5-fold in 6 M guanidinium chloride, 100 mM phosphate, pH 2 buffer for LC-MS analysis (19 µL, 5 µg). 36 mM diiodomethane in degassed acetonitrile (76 µL) was then added to the mixture. At t = 30 min and t = 18 h, aliquots were taken, diluted as above, and analyzed by LC-MS (38 µL injection volume, 5 µg, **Figure S50**). The product was not isolated. The reaction was conducted in parallel with KRpep(‘SH’), Zpep(‘SH’), and Ac-Zpep(‘SH’).

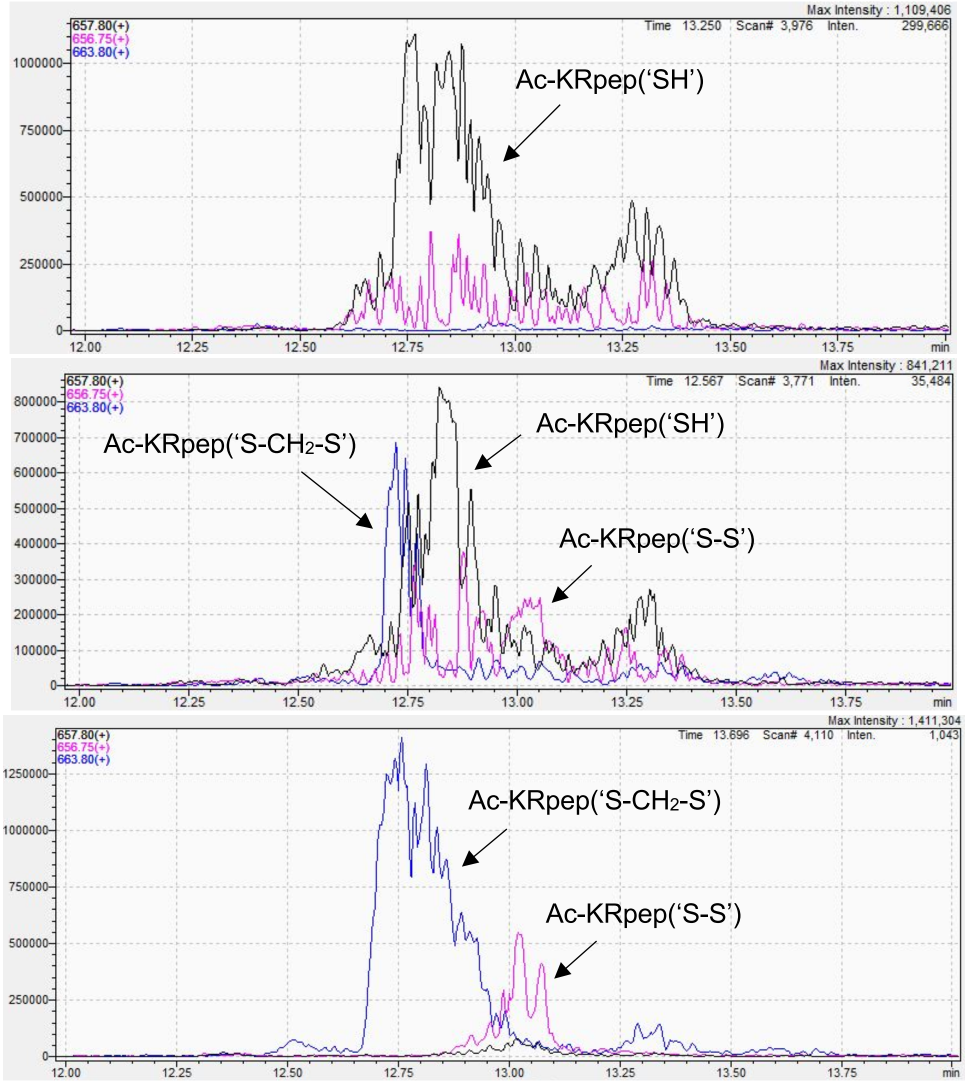

Figure S50. Extracted ion chromatograms of the reaction mixture at t = 0 (top), at t = 30 min (middle), and t = 18 h (bottom). Traces corresponding to Ac-KRpep('SH'), Ac-KRpep('S-S'), and Ac-KRpep('S-CH_2-S') are in black, magenta, and blue respectively.

Diiodomethane cyclization of Ac-Zpep('SH') (A*STAR conditions)

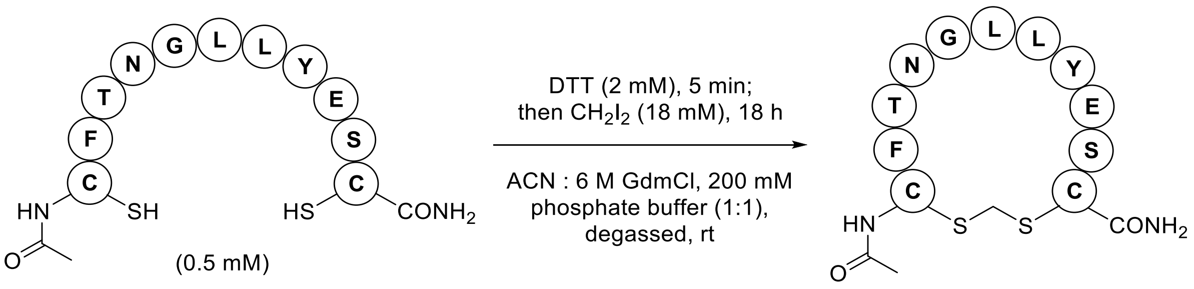

Ac-Zpep('SH') (0.1 mg, 80.1 nmol) was dissolved in the 100 mM dithiothreitol solution from Page S46 (3.1 µL) and 6 M guanidinium chloride, 200 mM phosphate, pH 9 buffer (74 µL) and the mixture was left to react for 5 min. A t_0 aliquot was diluted 5-fold in 6 M guanidinium chloride, 100 mM phosphate, pH 2 buffer for LC-MS analysis (19 µL, 5 µg). 36 mM diiodomethane in degassed acetonitrile (77 µL) was then added to the mixture. At t = 30 min and t = 18 h, aliquots were taken, diluted as above, and analyzed by LC-MS (39 µL injection volume, 5 µg, **Figure S51**). The product was not isolated. The reaction was conducted in parallel with KRpep('SH'), Zpep('SH'), and Ac-KRpep('SH').

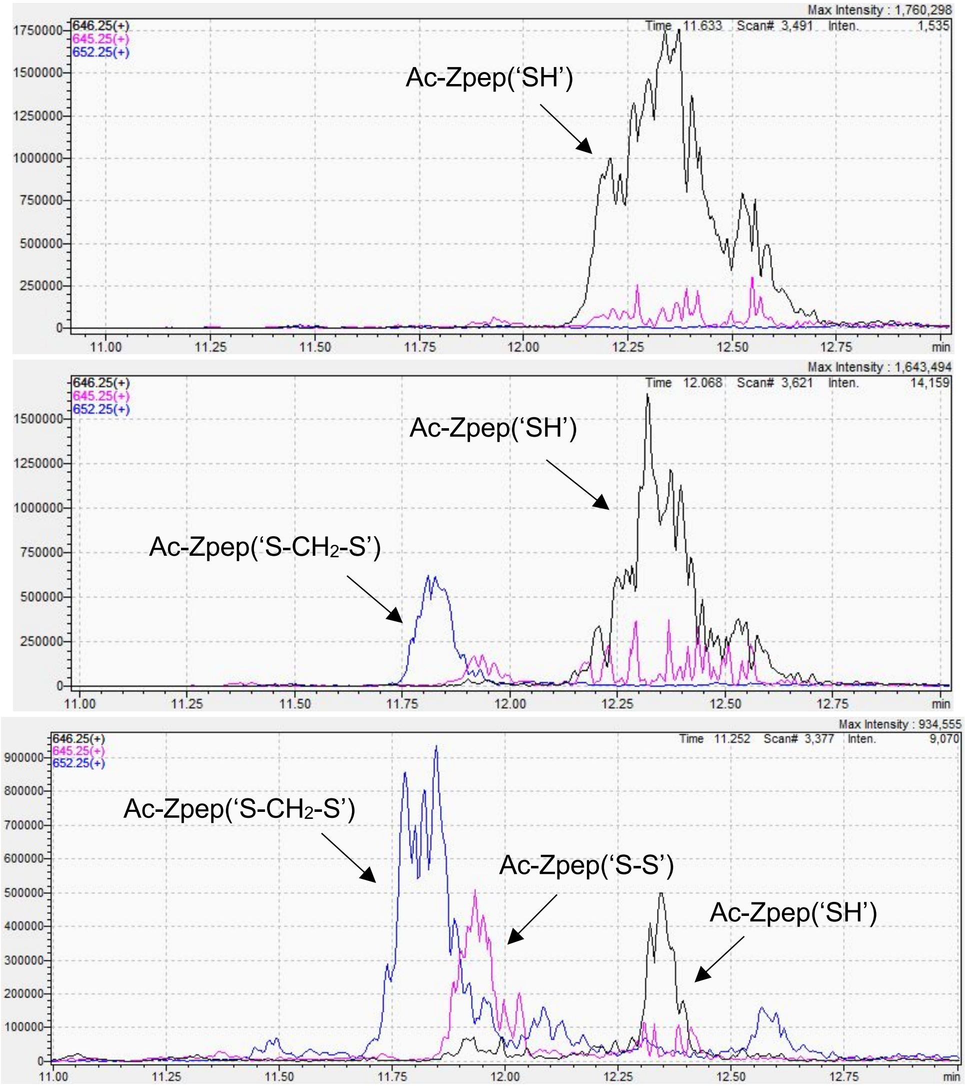

Figure S51. Extracted ion chromatograms of the reaction mixture at t = 0 (top), at t = 30 min (middle), and t = 18 h (bottom). Traces corresponding to Ac-Zpep('SH'), Ac-Zpep('S-S'), and Ac-Zpep('S-CH_2-S') are in black, magenta, and blue respectively.

Acetamide capping of PMI('SH')

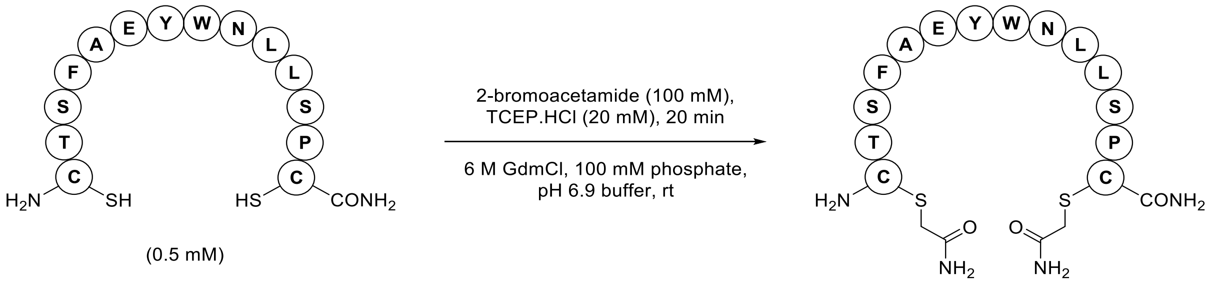

Preparation of reaction buffer

The reaction buffer was prepared by dissolving tris(2-carboxyethyl)phosphine hydrochloride (137.59 mg, 0.48 mmol) and 2-bromoacetamide (331.30 mg, 2.4 mmol) in 6 M guanidinium chloride, 100 mM phosphate, pH 6.9 buffer (12 mL). The resulting solution was adjusted back to pH 6.9 using aqueous NaOH.

Reaction

PMI('SH') (2.5 mg, 1.53 μmol) was dissolved in 6 M guanidinium chloride, 100 mM phosphate, pH 6.9 buffer (1.53 mL). A t_0 aliquot was taken and diluted 5-fold in 6 M guanidinium chloride, 200 mM phosphate, pH 3 buffer for LC-MS analysis (15 μL injection volume, 5 μg). The reaction buffer (1.53 mL) was added and left to react for 20 min. The reaction was quenched by the addition of dithiothreitol (94.50 mg, 0.61 mmol). The solution was aliquoted for LC-MS analysis (6.1 μL injection volume, 5 μg) and subjected to large-scale solid phase extraction (Page S23). Aliquots of the eluates were diluted 10-fold in Solvent A and analyzed by LC-MS (37 μL injection volume, 5 μg, **Figure S52**). The eluates were flash frozen and lyophilized to afford PMI('S-acetamide') as a white solid (2.69 mg, 1.54 μmol, 101% yield). The reaction was conducted in parallel with PMI('SH') library.

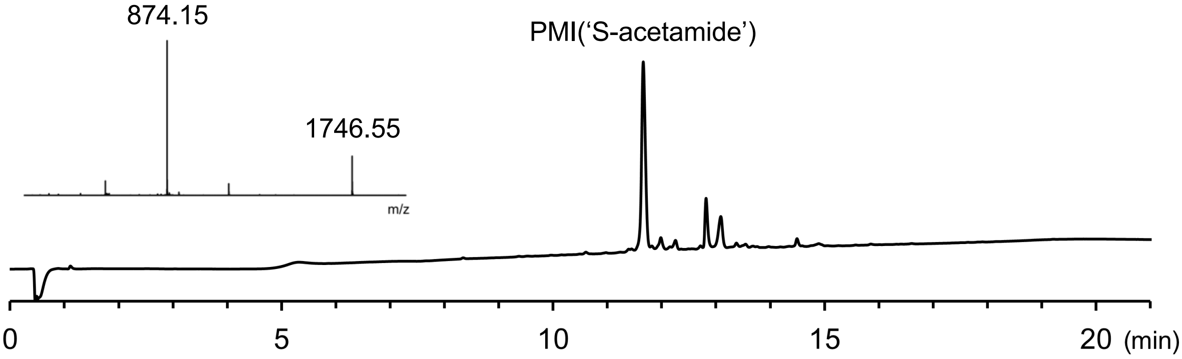

Figure S52. UV chromatogram from LC-MS analysis of the reaction product, with inlaid mass spectrum integrated across the principal UV component. Monoisotopic *m/z* calculated for $[M+H]^+$ 1746.75, found 1746.55.

Acetamide capping of PMI('SH') library

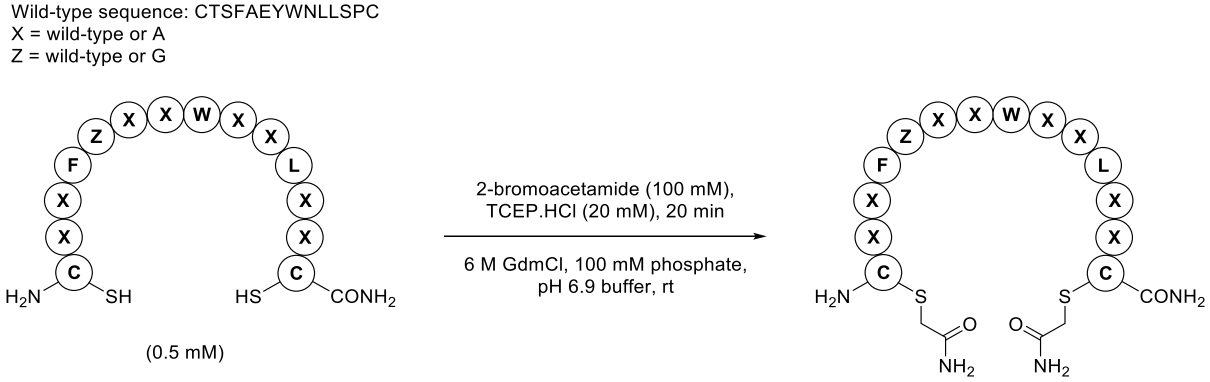

Reaction

PMI('SH') library (2.5 mg, 1.71 μmol; 3.33 nmol/peptide nominal) was dissolved in 6 M guanidinium chloride, 100 mM phosphate, pH 6.9 buffer (1.71 mL). A t_0 aliquot was taken and diluted 5-fold in 6 M guanidinium chloride, 200 mM phosphate, pH 3 buffer for LC-MS analysis (17 μL injection volume, 5 μg). The reaction buffer from the preceding entry (Page S76) was added (1.71 mL) and left to react for 20 min. The reaction was quenched by the addition of dithiothreitol (105.38 mg, 0.68 mmol). The solution was aliquoted for LC-MS analysis (6.8 μL injection volume, 5 μg) and subjected to large-scale solid phase extraction (Page S23). Aliquots of the eluates were diluted 10-fold in Solvent A and analyzed by LC-MS (37 μL injection volume, 5 μg, **Figure S53**). The eluates were flash frozen and lyophilized to afford PMI('S-acetamide') library as a white solid (2.42 mg, 1.53 μmol; 2.99 nmol/peptide nominal, 90% yield). The reaction was conducted in parallel with PMI('SH').

Preparation of stock solutions for nLC-MS analysis

The PMI('S-acetamide') library (2.42 mg, 1.53 μmol total; 2.99 nmol/peptide nominal) was dissolved in 50/50 Solvent A/B (613 μL) to give a nominally 2.5 mM solution, which was then centrifuged (15,000 rpm, 3 min). The supernatant (2 μL) was diluted in MS grade water (18 μL), and the peptide concentration of the resulting solution was analyzed with a Thermo Scientific™ NanoDrop™ Eight UV-Vis spectrophotometer. Based on the measured peptide concentration, the supernatant of the nominally 2.5 mM solution was then serially diluted using MS Mobile Phase (2.5% MS grade acetonitrile, 97.5% MS grade water, 0.05% acetic acid) spiked with 2 fmol/peptide/μL Pierce Peptide Retention Time Calibration Mixture to yield 20, 10, and 2 fmol/peptide/μL solutions of the PMI('S-acetamide') library. The solutions were then analyzed by nLC-MS ('standard run', Page S25).

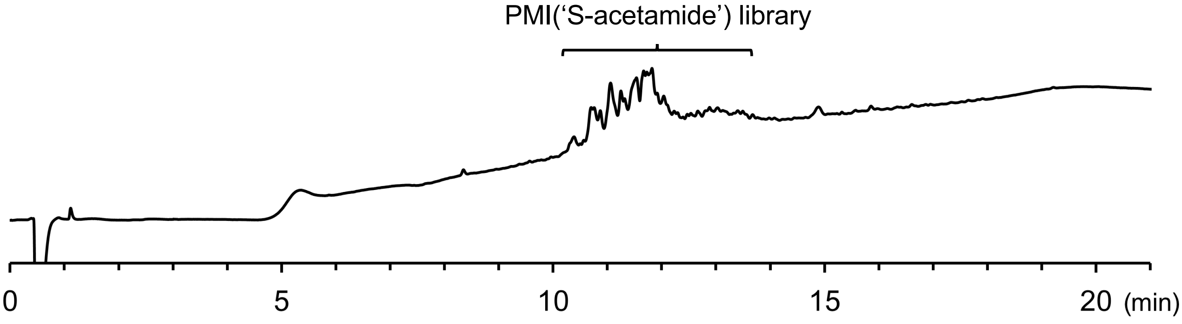

Figure S53. UV chromatogram of the reaction product.

α,α'-Dibromo-*m*-xylene cyclization of PMI('SH')

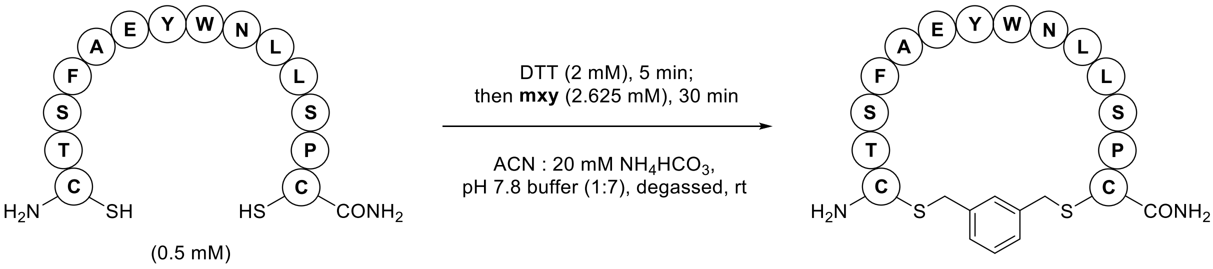

Ammonium bicarbonate (47.43 mg, 0.6 mmol) was dissolved in degassed deionized water (30 mL) to give an aqueous 20 mM ammonium bicarbonate, pH 7.8 buffer. Dithiothreitol (15.43 mg, 0.1 mmol) was dissolved in the ammonium bicarbonate buffer (1 mL) to give a 0.1 M dithiothreitol solution. PMI('SH') (5 mg, 3.06 μmol) was dissolved in ammonium bicarbonate buffer (5.24 mL). The 0.1 M dithiothreitol solution (123 μL) was added and left to react for 5 min. A t_0 aliquot was taken for LC-MS analysis (5.4 μL injection volume, 5 μg). 21 mM α,α'-dibromo-*m*-xylene in degassed acetonitrile (766 μL) was then added and left to react for 30 min. The reaction mixture was washed with cold diethyl ether (3x, approx. 6.1 mL each), and excess ether was evaporated over a stream of inert gas. The solution was then diluted 4-fold with 6 M guanidinium chloride, 100 mM phosphate, pH 6.9 buffer (18.4 mL), filtered through a 0.45 μm nylon filter, and aliquoted for LC-MS analysis (24 μL injection volume, 5 μg). The filtered solution was subjected to large-scale solid phase extraction (Page S23). Aliquots of the eluates were diluted 10-fold in Solvent A for LC-MS analysis (19 μL injection volume, 5 μg, **Figure S54**), and the eluates were flash frozen, and lyophilized to yield PMI('S-**mxy**-S') as a white solid (3.18 mg, 1.83 μmol, 60% yield). The reaction was conducted in parallel with PMI('SH') library.

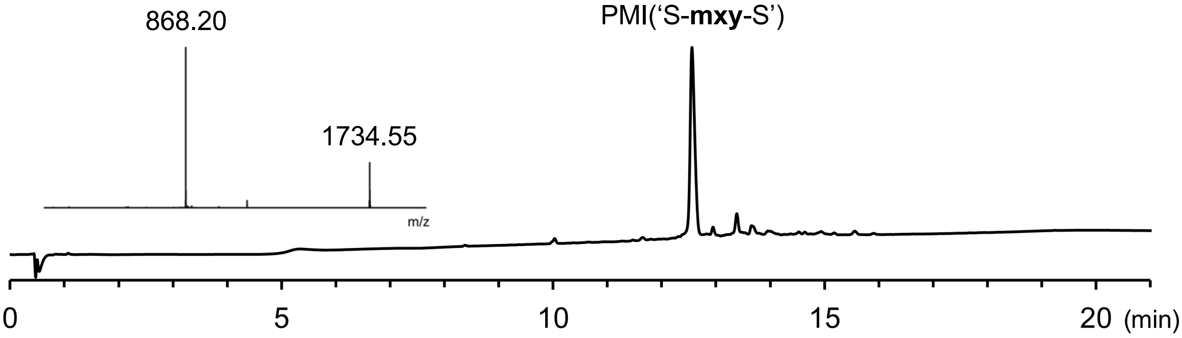

Figure S54. UV chromatogram from LC-MS analysis of the reaction product, with inlaid mass spectrum integrated across the principal UV component. Monoisotopic *m/z* calculated for $[M+H]^+$ 1734.76, found 1734.55.

α,α’-Dibromo-*m*-xylene cyclization of PMI(‘SH’) library

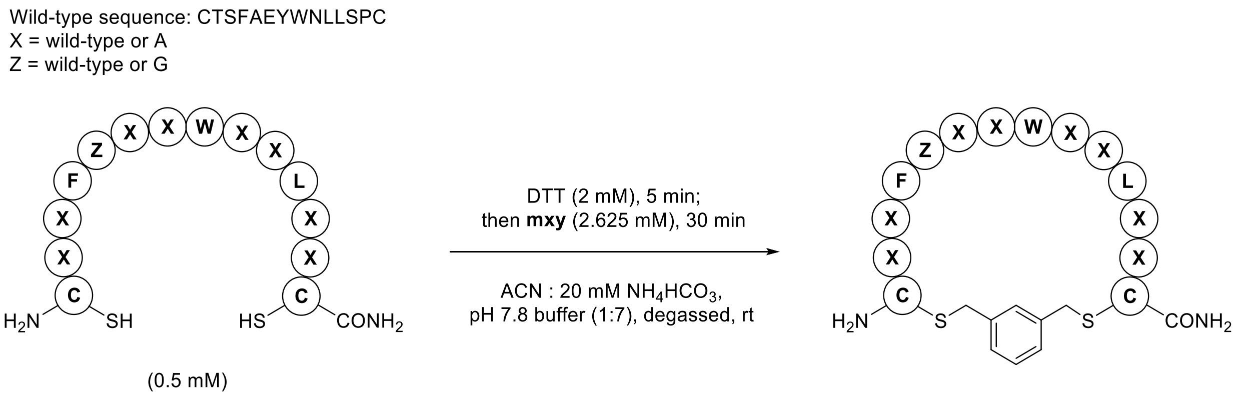

PMI(‘SH’) library (10.92 mg, 7.46 µmol; 14.6 nmol/peptide nominal) was dissolved in ammonium bicarbonate buffer from the preceding entry (Page S78) (12.75 mL). The 0.1 M dithiothreitol solution from the preceding entry (Page S78) (298 µL) was added and left to react for 5 min. A t_0 aliquot was taken for LC-MS analysis (6 µL injection volume, 5 µg). 21 mM α,α’-dibromo-*m*-xylene in degassed acetonitrile (1.86 mL) was then added and left to react for 30 min. The reaction mixture was washed with cold diethyl ether (3x, approx. 14.9 mL each), and excess ether was evaporated over a stream of inert gas. The resulting solution was split evenly into 2 portions, each of which was diluted 4-fold with 6 M guanidinium chloride, 100 mM phosphate, pH 6.9 buffer (3 vol., approx. 22.4 mL each), filtered through a 0.45 µm nylon filter, and aliquoted for LC-MS analysis (27 µL injection volume, 5 µg). The filtered solution was subjected to large-scale solid phase extraction (Page S23). Aliquots of the eluates were diluted 10-fold in Solvent A for LC-MS analysis (8.6 µL injection volume, 5 µg, **Figure S55**), and the eluates were flash frozen, and lyophilized to yield PMI(‘S-**mxy**-S’) library as a white solid (2.92 mg, 1.86 µmol; 3.64 nmol/peptide nominal, 25% yield). The reaction was conducted in parallel with PMI(‘SH’).

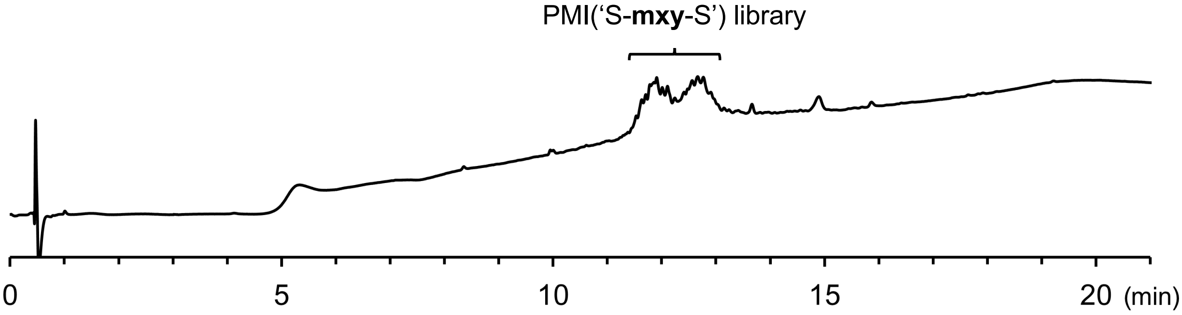

Figure S55. UV chromatogram of the reaction product.

PITC installation on PMI('S-mxy-S')

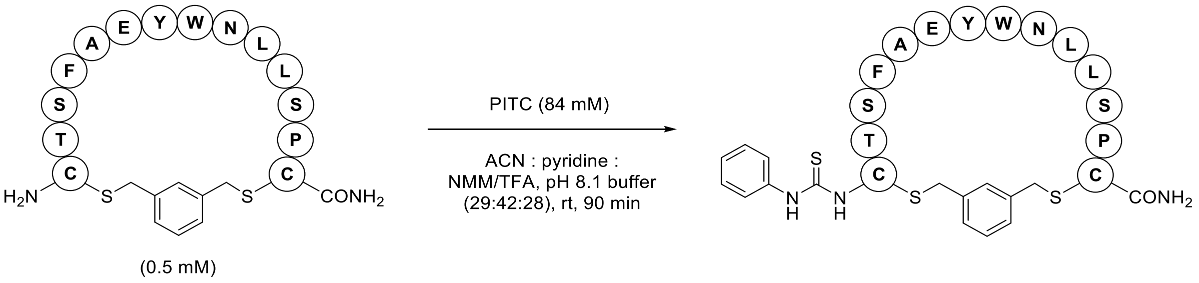

Preparation of pH 8.1 NMM/TFA buffer

N-methylmorpholine (1.4 mL) and trifluoroacetic acid (0.19 mL) were diluted in deionized water (8.4 mL) and the pH was adjusted to 8.1.

Reaction

Pyridine (5.46 mL) and pH 8.1 NMM/TFA buffer (3.64 mL) were combined to give the pyr/NMM buffer. PMI('S-**mxy**-S') (3.18 mg, 1.83 µmol) was dissolved in the pyr/NMM buffer (2.57 mL). An aliquot was diluted 10-fold in Solvent A and analyzed by LC-MS (40 µL injection volume, 5 µg). Phenyl isothiocyanate (130 µL) was diluted in acetonitrile (3.77 mL) to give a 3.33% (v/v) PITC solution, which was added (1.1 mL) to the peptide solution. The mixture was then left to react for 90 min, after which it was split into two equal portions, which were quenched with Solvent A (16.5 mL each). The mixtures were centrifuged (4,000 rpm, Lot 1: 30 min; Lot 2: 15 min) and the supernatants were carefully decanted with a syringe. Aliquots of the supernatants were centrifuged until clear (15080 rpm, 5 min) and taken for LC-MS analysis (40 µL injection volume, 3.74 µg). The supernatants were then washed with diethyl ether (3x, approx. 18.3 mL each). Excess ether was evaporated over a stream of inert gas and the resulting solution was aliquoted for LC-MS analysis (40 µL injection volume, 3.74 µg). The solutions were subjected to large-scale solid phase extraction (Page S23) with both lots being loaded onto the same cartridge. Aliquots of the eluates were diluted 10-fold in Solvent A and then subjected to LC-MS analysis (29 µL injection volume, 5 µg, **Figure S56**). Eluates were flash frozen and lyophilized to afford PITC-PMI('S-**mxy**-S') as a white solid (0.29 mg, 0.16 µmol, 8% yield). The reaction was conducted in parallel with PMI('S-**mxy**-S') library.

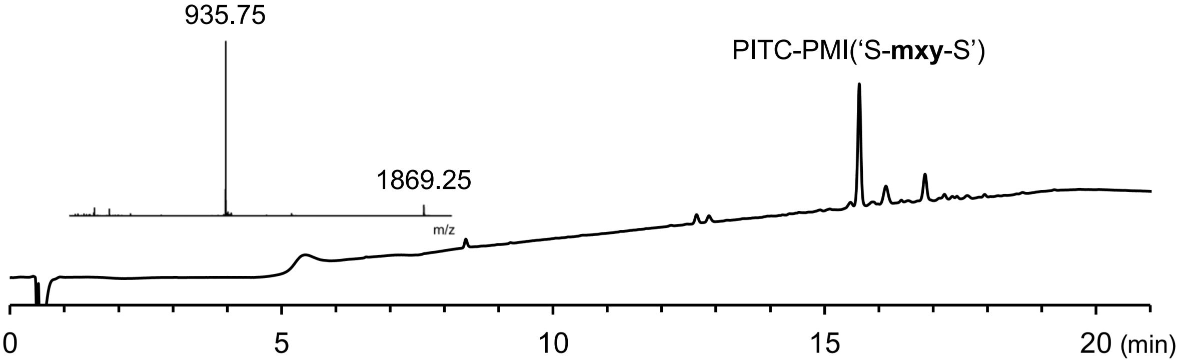

Figure S56. UV chromatogram from LC-MS analysis of the reaction product, with inlaid mass spectrum integrated across the principal UV component. Monoisotopic *m/z* calculated for $[M+H]^+$ 1869.77, found 1869.25.

PITC installation on PMI('S-mxy-S') library

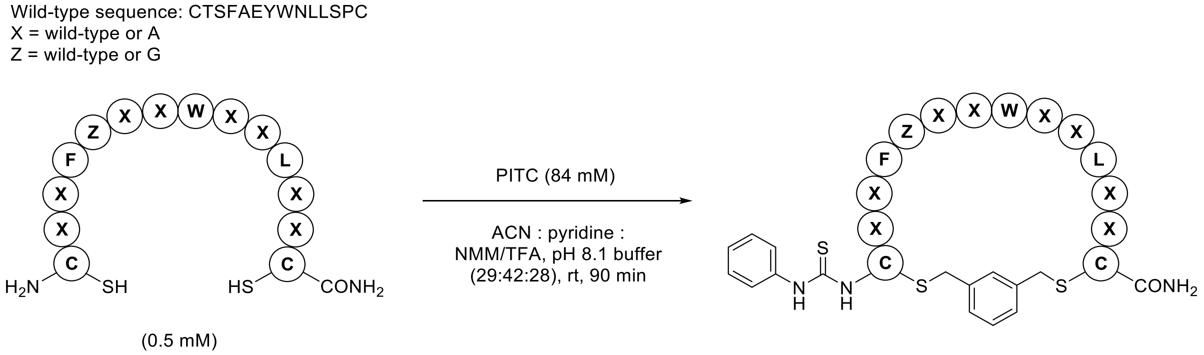

PMI('S-**mxy**-S') library (2.92 mg, 1.86 µmol total; 3.64 nmol/peptide nominal) was dissolved in the pyr/NMM buffer from the preceding entry (Page S80) (2.61 mL). An aliquot was diluted 10-fold in Solvent A and analyzed by LC-MS (40 µL injection volume, 4.5 µg). The 3.33% (v/v) PITC solution from the preceding entry (Page S80) (1.18 mL) was added to the peptide solution. The mixture was then left to react for 90 min, after which it was split into two equal portions, which were quenched with Solvent A (16.8 mL each). The mixtures were centrifuged (4,000 rpm, Lot 1: 30 min; Lot 2: 15 min) and the supernatants were carefully decanted with a syringe. Aliquots of the supernatants were centrifuged until clear (15080 rpm, 5 min) and taken for LC-MS analysis (40 µL injection volume, 3.4 µg). The supernatants were then washed with diethyl ether (3x, approx. 18.6 mL each). Excess ether was evaporated over a stream of inert gas and the resulting solution was aliquoted for LC-MS analysis (40 µL injection volume, 3.4 µg). The solutions were subjected to large-scale solid phase extraction (Page S23) with both lots being loaded onto the same cartridge. Aliquots of the eluates were diluted 10-fold in Solvent A and then subjected to LC-MS analysis (32 µL injection volume, 5 µg, **Figure S57**). Eluates were flash frozen and lyophilized to afford PITC-PMI('S-**mxy**-S') library as a white solid (1.77 mg, 1.04 µmol; 2.03 nmol/peptide nominal, 56% yield). The reaction was conducted in parallel with PMI('S-**mxy**-S').

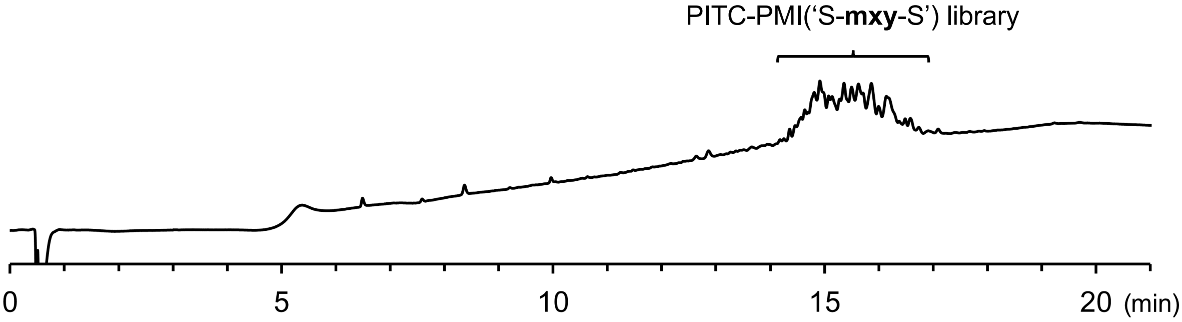

Figure S57. UV chromatogram of the reaction product.

Edman linearization of PITC-PMI('S-mxy-S')

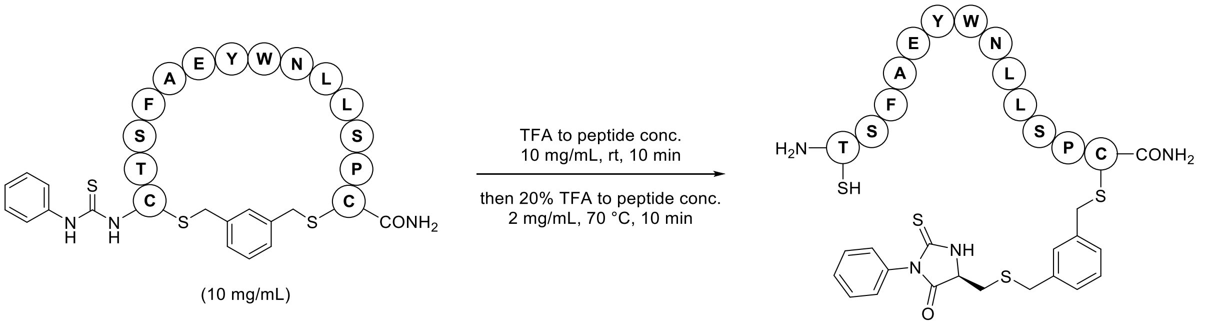

PITC-PMI('S-**mxy**-S') (0.29 mg, 0.16 µmol) was dissolved in trifluoroacetic acid (29 µL) and left to react for 10 min. The solution was diluted 5-fold with deionized water (116 µL) and heated to 70 °C for 10 min. The solution was then diluted with aqueous 6 M guanidinium chloride, 100 mM phosphate, pH 7 buffer (435 µL), and subjected to small-scale solid phase extraction (Page S23). Aliquots of the eluates were diluted 10-fold in Solvent A and subjected to LC-MS analysis (34 µL injection volume, 5 µg, **Figure S58**), and the eluates were flash frozen and lyophilized to afford linearized PMI('S-**mxy**-S') as a white solid (0.18 mg, 0.45 µmol, 62% yield). The reaction was conducted in parallel with PITC-PMI('S-**mxy**-S') library.

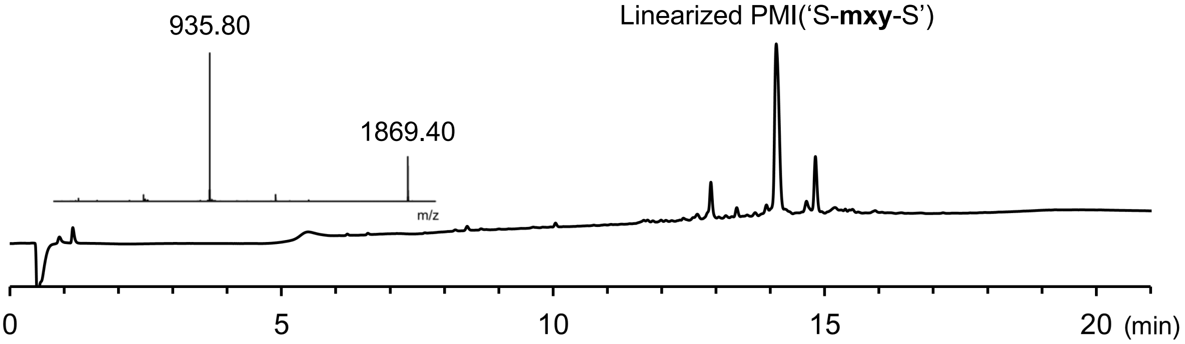

Figure S58. UV chromatogram from LC-MS analysis of the reaction product, with inlaid mass spectrum integrated across the principal UV component. Monoisotopic *m/z* calculated for $[M+H]^+$ 1869.77, found 1869.40.

<u>Edman linearization of PITC-PMI('S-mxy-S') library</u>

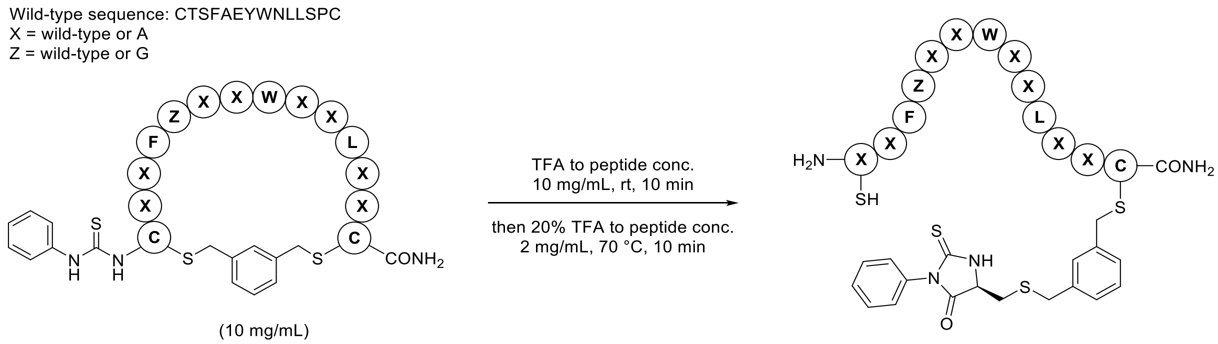

PITC-PMI('S-**mxy**-S') library (0.89 mg, 0.52 µmol total; 1.02 nmol/peptide nominal) was dissolved in trifluoroacetic acid (89 µL) and left to react for 10 min. The solution was diluted 5-fold with deionized water (354 µL) and heated to 70 °C for 10 min. The solution was then diluted with aqueous 6 M guanidinium chloride, 100 mM phosphate, pH 7 buffer (1.33 mL), and subjected to small-scale solid phase extraction (Page S23). Aliquots of the eluates were diluted 10-fold in Solvent A and subjected to LC-MS analysis (11 µL injection volume, 5 µg, **Figure S59**), and the eluates were flash frozen and lyophilized to afford linearized PMI('S-**mxy**-S') library as a white solid (0.76 mg, 0.45 µmol total; 0.88 nmol/peptide nominal, 86% yield). The reaction was conducted in parallel with PITC-PMI('S-**mxy**-S').

Preparation of stock solutions for nLC-MS analysis

The linearized PMI('S-**mxy**-S') library (0.76 mg, 0.45 µmol total; 0.88 nmol/peptide nominal) was dissolved in 50/50 Solvent A/B (179 µL) to give a nominally 2.5 mM solution, which was then centrifuged (15,000 rpm, 3 min). The supernatant (2 µL) was diluted in MS grade water (18 µL), and the peptide concentration of the resulting solution was analyzed with a Thermo Scientific™ NanoDrop™ Eight UV-Vis spectrophotometer. Based on the measured peptide concentration, the supernatant of the nominally 2.5 mM solution was then serially diluted using MS Mobile Phase (2.5% MS grade acetonitrile, 97.5% MS grade water, 0.05% acetic acid) spiked with 2 fmol/peptide/µL Pierce Peptide Retention Time Calibration Mixture to yield 40, 20, 10, and 2 fmol/peptide/µL solutions of the linearized PMI('S-**mxy**-S') library. The solutions were then analyzed by nLC-MS ('extended run', Page S25).

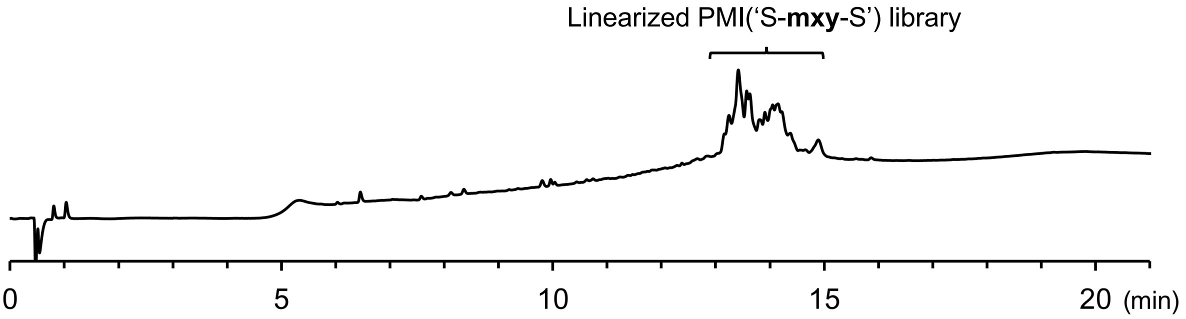

Figure S59. UV chromatogram of the reaction product.
